# Information propagation through enzyme-free catalytic templating of DNA dimerization with weak product inhibition

**DOI:** 10.1038/s41557-025-01831-x

**Published:** 2025-06-05

**Authors:** Javier Cabello-Garcia, Rakesh Mukherjee, Wooli Bae, Guy-Bart V. Stan, Thomas E. Ouldridge

**Affiliations:** 1 Department of Bioengineering, Imperial College London, London, UK; 2 Imperial College Centre for Engineering Biology, Imperial College London, London, UK; 3 School of Mathematics and Physics, University of Surrey, Guildford, UK

**Keywords:** Biophysical chemistry, DNA, Reaction kinetics and dynamics

## Abstract

Information propagation by sequence-specific, template-catalysed molecular assembly is a key process facilitating life’s biochemical complexity, yielding thousands of sequence-defined proteins from only 20 distinct building blocks. However, exploitation of catalytic templating is rare in non-biological contexts, particularly in enzyme-free environments, where even the template-catalysed formation of dimers is challenging. Typically, product inhibition—the tendency of products to bind to templates more strongly than individual monomers—prevents catalytic turnover. Here we present a rationally designed enzyme-free system in which a DNA template catalyses, with weak product inhibition, the production of sequence-specific DNA dimers. We demonstrate selective templating of nine different dimers with high specificity and catalytic turnover, then we show that the products can participate in downstream reactions, and finally that the dimerization can be coupled to covalent bond formation. Most importantly, our mechanism demonstrates a design principle for constructing synthetic molecular templating systems, a first step towards applying this powerful motif in non-biological contexts to construct many complex molecules and materials from a small number of building blocks.

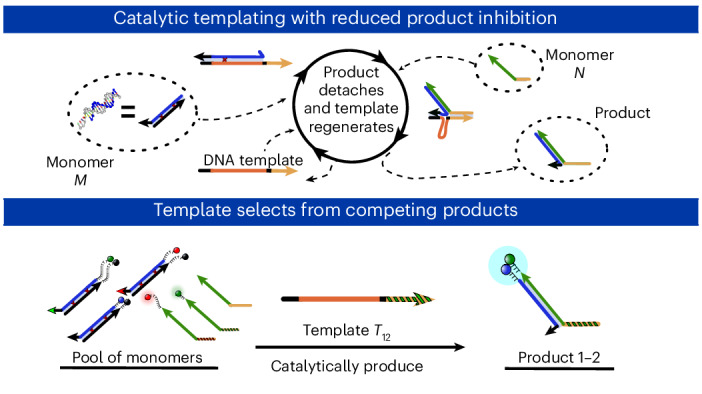

## Abstract (web summary)

Information-bearing templates that catalyse the assembly of complex macromolecules are a central motif of natural biochemistry, but their power remains largely unexplored in synthetic contexts. Enzyme-free templating of DNA dimerization has now been demonstrated, using DNA nanotechnology to ensure that the templates are effective information-propagating catalysts.

## Main

Cells produce tens of thousands of distinct proteins from 20 amino acids^1^. Were these amino acids to polymerize in isolation and then fold, it would result in the formation of a heterogeneous population of products; the amino acid monomers do not encode enough information in their interactions alone to direct the assembly of so many specific proteins from the astronomically large catalogue of possible products^2^. Instead, biology assembles complex macromolecules from simple monomers with high precision templating processes—RNA transcription and protein translation—wherein sequence information is efficiently copied from a copolymer template into a newly produced daughter copolymer^3^. Mechanistically, this copying involves sequence-specific recognition interactions between template and daughter. Equally, however, these interactions must eventually be disrupted so that the daughter dissociates, allowing sequence-directed folding of the daughter^4^ and reuse of the template^5–8^.

Although biological templating relies upon enzyme-catalysed reactions, there has been wide interest in rationally engineering enzyme-free templating mechanisms to assemble specific molecules^9^. Many researchers seek to use templating to enhance reactions that have an otherwise low yield^10,11^. Others pursue templating as a pathway to synthesize new, complex, sequence-controlled polymers^12,13^ or even use biological polymers, such as DNA, as an easily synthesized template for directing combinatorial screenings to discover new materials and molecules with therapeutic potential^14,15^. More ambitiously, biologically relevant polymers are used as templates to understand the origin of life or engineer synthetic life^6,16–19^.

When designing enzyme-free templating systems, one of the biggest challenges, rather than efficient monomer recognition, is producing templates that act effectively as catalysts. To ensure a reliable copying system, the reaction of monomers must be slow in solution but occur rapidly and with high turnover in the presence of the catalyst template. To achieve this high turnover, the assembled products must be efficiently released from the template to ensure its reusability^5,6^. If the templates were not reusable, a new, highly specific template would need to be assembled for each product macromolecule, and creating the template itself would become a self-assembly challenge of similar magnitude to the assembly of the product, defeating the purpose of templating^7,8^.

The tendency of products to remain bound to catalysts is known as product inhibition and occurs for all types of catalyst^20^. If the product–catalyst complex’s lifetime is similar to that of the substrate–catalyst complex, the product will ‘compete’ with the monomers for binding. If product binding is irreversible, it will prevent catalysis altogether. Product inhibition is a particular challenge for templated assembly of dimers and longer molecules. After polymerization of the monomers, the now interconnected monomers typically bind the template more strongly due to cooperativity. Indeed, in simple models, the free-energy change of dissociation increases linearly with polymer length^21^. This cooperative effect results in stronger inhibition as the polymer length increases.

As a result of cooperative product inhibition, the construction of catalysts for enzyme-free templating has seen limited progress. Several dimer templating systems have been demonstrated, with varying degrees of product inhibition and catalytic efficiency^17,19,22,23^. These systems are, however, not generalizable to longer templates. They lack a mechanism for overcoming product inhibition while ensuring that the weakly binding, partially formed products do not prematurely detach from the template^7^. Alternatively, many groups have circumvented product inhibition by cycling external conditions to first favour growth on the template and then separation of product and template^24–28^. Others have created environments with a non-chemical supply of energy—temperature gradients^18^ or mechanical agitation^29,30^—that allow growth and separation. Although early life may have used templating driven by external conditions, such systems lack the versatility and flexibility of the autonomous, chemically driven templating processes, as observed in extant biology.

In this work, we implement, using solely DNA, sequence-specific, autonomous, chemically driven catalytic templating of DNA dimerization with low product inhibition. As is standard in DNA nanotechnology^27,31–34^, our products are held together by DNA base pairing, rather than covalent bonds^19,26,28–30,35^. Modular motifs based on DNA base pairing have been used to demonstrate remarkable functionality^31,32,36^, but common primitives are not well suited to information propagation by catalytic templating of molecular assembly, as defined in Supplementary Note 1, in the absence of non-chemical or non-autonomous driving of dissociation. To demonstrate catalytic templating, we employ a mechanism that diverts free energy from dimerization to weaken the bonds of the reacting monomers with the template. Most previous work that channels the free energy of dimerization in this way has had limited efficacy^37^ or involved templates that are not reusable catalysts^38,39^. Lewandowski et al. have demonstrated effective catalysis of assembly for products of up to four units in length but without the ability to selectively template the assembly of different sequences of monomers and hence propagate information^40^. By contrast, we demonstrate that a DNA-based template can perform sequence-specific catalysis of the formation of one out of nine different dimers in competition, with high turnover and low product inhibition. We then show that the mechanism can be embedded within a multistep network and used to template covalent bond formation. Moreover, the design is, in principle, generalizable to the templated copying of longer templates, further increasing the potential of DNA as a tool to efficiently explore the vast chemical space of sequence-defined polymers.

## Results

### Catalytic mechanism

**Fig. 1:**
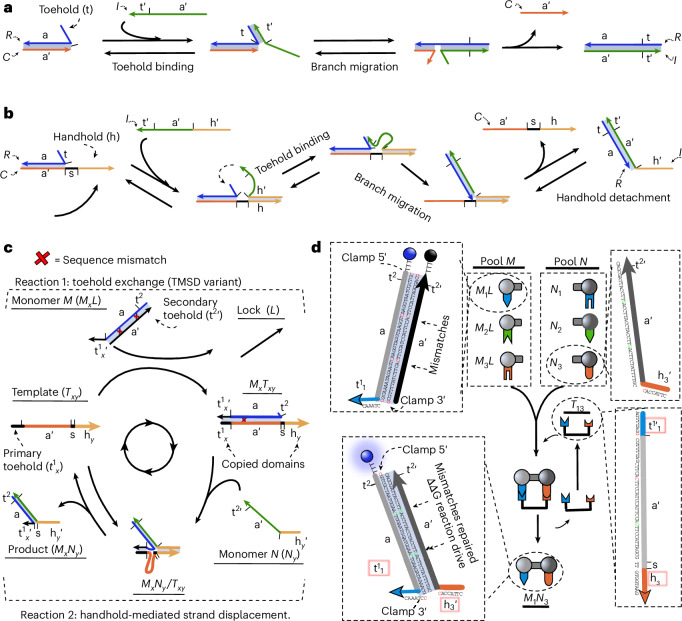
DNA strand displacement topologies, catalysis mechanism of the template and system design. **a**, TMSD. Binding to the toehold (t) domain in the target DNA strand (*R*) mediates displacement of the incumbent (*C*) by the invader (*I*). After displacement, the toehold is cooperatively sequestered in duplex *IR*. **b**, HMSD. When *I* binds to the handhold (h) domain in *C*, the effective concentration of *I* increases in the vicinity of *R*, enhancing displacement. The reversible nature of handhold binding allows *IR* to detach. **c**, The DNA-based catalytic templating system. The DNA monomers (*M*_*x*_*L* and *N*_*y*_) can dimerize after binding to a DNA template (*T*_*xy*_), exploiting first toehold exchange (a TMSD variant) then HMSD. Dimerization between the monomers weakens the interaction with *T*_*xy*_, allowing *M*_*x*_*N*_*y*_ to detach and for *T*_*xy*_ to undergo another dimerization cycle. **d**, The specific-sequence domains of *T*_*xy*_ can trigger the dimerization of a specific *M*_*x*_*L*, *N*_*y*_ pair from pools of monomers in solution. The result is a product distribution enriched in *M*_*x*_*N*_*y*_ dimers with t and h domains (red boxes) complementary to *T*_*xy*_, propagating the sequence information in the template. Any *x*,*y* combination is possible, with the dimerization domain **a** initially hidden by *L*, inhibiting any direct reaction in the absence of *T*_*xy*_. The edges of the *M*_*x*_*L* duplex have additional bases—‘clamps’—suppressing any leak reactions. The two mismatched base pairs in the a domain of *M*_*x*_*L* ensure that dimerization is thermodynamically favoured. The DNA strands are represented by domains (contiguous sequences of nucleotides considered to hybridize as a unit). The domains are labelled with a lowercase letter; a prime symbol indicates complementarity; for example, a′ binds to a.

The proposed system consists of two DNA reactions: toehold-mediated strand displacement (TMSD) and handhold-mediated strand displacement (HMSD). TMSD (Fig. 1a) is central to dynamic DNA nanotechnology^33^. The reaction involves three nucleic acid strands—an invader *I*, an incumbent *C* and a target *R*. Initially, *R* and *C* form a duplex separate from *I*. However, *R* is typically longer than *C* and presents a single-stranded ‘toehold’ overhang. *I* is complementary to the whole of *R* and can thus bind to the toehold and then displace *C* from its binding with *R*. The toeholds act as recognition domains for the displacement, with longer toeholds increasing the probability of successful displacement, resulting in an exponential increase in displacement rate with toehold length up to a plateau at around six to seven nucleotides at room temperature^34^.

HMSD (Fig. 1b) is a recently proposed motif that adds new functionality to strand displacement networks^38,41^. It operates similarly to TMSD, but the initial recognition domain (the ‘handhold’) is in *C* rather than *R*. This change in topology is ideal for templating; the binding of *I* to *R* can be templated by the recognition between *C* and *I*, just as recognition between DNA and RNA nucleotides templates the polymerization of RNA during transcription. Furthermore, the binding of *I* to *R* disrupts the binding of *R* to *C*; the displacement process rips apart the *CR* duplex, allowing the *IR* duplex to spontaneously detach^38^.

By combining TMSD and HMSD, we construct a system in which a template can recognize molecules in solution and catalyses their dimerization. In our proposed templating system, shown mechanistically in Fig. 1c and schematically in Fig. 1d, monomers are drawn from two pools of DNA strands, labelled *M* and *N*. Both *M* and *N* monomers possess a long ‘dimerization’ domain and a short ‘recognition’ domain. These recognition domains (toehold in *M* and handhold in *N*) are variable, and we use *M*_*x*_ and *N*_*y*_ with *x*,*y* = 1, 2, 3 to differentiate the monomers. The ‘dimerization’ domains are independent of *x* and *y* and complementary, so any *M*_*x*_*N*_*y*_ dimer can form with roughly equal stability.

Direct dimerization is suppressed by a ‘lock’ strand *L*. Instead, sequence-specific dimerization is catalysed by a template *T*_*xy*_ containing recognition domains complementary to those on both *M*_*x*_ and *N*_*y*_, and a dimerization domain complementary to the dimerization domain on *M*_*x*_. As a result, as illustrated in Fig. 1c, *T*_*xy*_ can first displace *L* from *M*_*x*_*L* via TMSD, and then *N*_*y*_ can displace *T*_*xy*_ from its duplex with *M*_*x*_ via HMSD, releasing the dimer *M*_*x*_*N*_*y*_ and completing the catalysis. The HMSD process is facilitated by a short ‘secondary’ toehold on *M*_*x*_ revealed during the first TMSD step (which is, therefore, formally a ‘toehold exchange’^34^).

Both the sequence-specific catalysis and the resultant concatenation of monomers are consistent with the definition of autonomous, chemically driven catalytic templating of assembly provided in Supplementary Note 1. Justification for the design at the level of sequences (Fig. 1d), including the use of mismatched base pairs to provide thermodynamic impetus while retaining catalytic control^42^, is provided in Methods.

### Optimization of system design for catalytic turnover

The programmability of DNA-based engineering allows a systematic optimization of the dimerization mechanism. We will consider variation of two key features: the lengths of the primary toehold (4–8 nt) and handhold (6–10 nt). We will use the notation *u*t/*v*h to refer to a system with a primary toehold of *u* nt and a handhold of *v* nt. Further discussion of system design principles is given in the Methods.

**Fig. 2:**
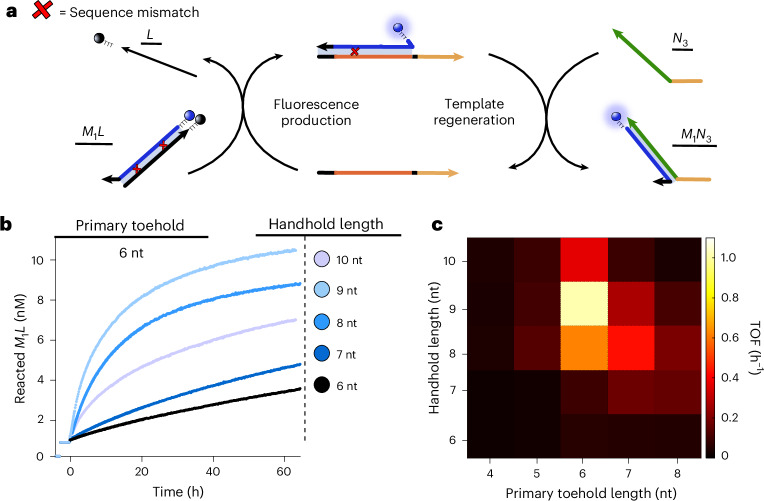
Initial TOF is optimized for toeholds and handholds of moderate length. **a**, The experimental setup. A small concentration of template (1 nM) is combined with a larger pool of *M*_1_*L* and *N*_3_ monomers (10 nM) for a range of primary toehold and handhold lengths. The catalytic turnover of *M*_1_*L* is reported by an increase in fluorescence signal. **b**, The example trajectories showing the concentration of reacted *M*_1_*L* over time, for a range of handhold lengths and a primary toehold of 6 nt. Increasing the handhold length above 9 nt results in a decrease of the *M*_1_*L* catalytic turnover due to increased product inhibition. These results illustrate how the overall reaction rate is a balance between the displacement and *M*_*x*_*N*_*y*_ detachment from *T*_*xy*_. The leak reaction in the absence of template could not be detected. Its magnitude for a monomer concentration of 100 nM is shown for *M*_1_*N*_3_ in Fig. 3c, and for all monomer combinations in Supplementary Fig. 12. The concentration of reacted *M*_1_*L* is inferred from the fluorescence data as outlined in Supplementary Note 5. **c**, The initial rate of reaction per unit of template (TOF) for each primary toehold and handhold condition. An optimum is obtained for a system with a primary toehold of 6 nt and a handhold of 9 nt (6t/9h) (1.01 ± 0.03 h^−1^) followed by condition 6t/8h (0.622 ± 0.009 h^−1^).

To compare the different systems with variable toehold and handhold lengths, we consider their initial turnover frequency (TOF), estimated from the initial rate of their dimerization reaction per the amount of *T*_*xy*_ catalyst present in the reaction solution^43^. The TOF indicates how effective the template catalyst *T* is at binding the substrates—via TMSD—converting them into a product—via HMSD—and then releasing that product. Specifically, we consider the recovery of fluorescence of fluorophore-labelled *M*_1_ as quencher-labelled *L* is displaced by the template *T*_13_ (Fig. 2a). Sustained catalytic turnover is observed when an excess of *N*_3_ is added to the mixture, and we use the resultant reaction rate to calculate the TOF.

As illustrated by the kinetics shown in Fig. 2b, 1 nM of *T*_13_ is capable of catalysing the transformation of high concentrations of *M*_1_*L* relative to the amount of template, indicating multiple rounds of catalytic turnover. The measured TOF reaches its maximum for 6t/9h at 1.01 ± 0.03 molecules turned over per hour per molecule of template. Here, the recognition domains are long enough to encourage binding to the template and high displacement rates but not so long that release of the product is slow, as happens for 6t/10h in Fig. 2b. It is notable from Fig. 2c that an increase in the primary toehold length tends to decrease the optimal handhold length and vice versa, indicating the importance of minimizing the cooperative binding of *MN* to *T*.

### Characterization of resistance to product inhibition

**Fig. 3:**
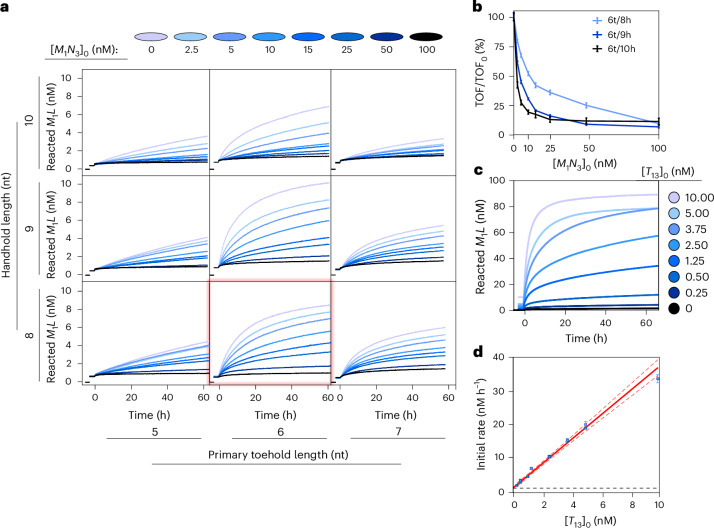
The optimal design of the HMSD-based catalyst experiences only moderate competitive product inhibition and achieves high turnover. **a**, The reacted monomer concentration [*M*_1_*L*] in a system with 10 nM *N*_3_, 10 nM *M*_1_*L*, 1 nM *T* and an initial non-fluorescent pool of products *M*_1_*N*_3_ at a range of concentrations ${[{M}_{1}{N}_{3}]}_{0}$. The condition 6t/8h, considered as optimal, is highlighted in red. **b**, The initial TOF at different ${[{M}_{1}{N}_{3}]}_{0}$ conditions for 6t/8h, 6t/9h and 6t/10h, obtained from the kinetics depicted in **a**. The symbols are centred on the best fit of the TOF to a single trajectory, with the height indicating a 95% confidence interval on that fit. Although 6t/9h has a higher TOF in the absence of [*M*_1_*N*_3_], 6t/8h combines rapid growth with a higher resistance to rate reduction at high ${[{M}_{1}{N}_{3}]}_{0}$. **c**, The turnover of *M*_1_*L* as inferred from fluorescence data, in experiments with 100 nM *M*_1_*L*, 100 nM *N*_3_ and variable concentrations of the template ${[{T}_{13}]}_{0}$ (6t/8h). A large proportion of *M*_1_*L* is observed to react, even for a concentration of ${[{T}_{13}]}_{0}$ 400 times lower than the number of monomers, reaching turnovers above 20 products per template. The template-free leak reactions are essentially negligible (0.32 ± 0.06 M^−1^ s^−1^) even compared with the lowest template concentration regimes. **d**, The initial rates of reactions from **c** and an additional set of replica experiments (Supplementary Note 7.2), as a function of ${[{T}_{13}]}_{0}$. The symbols are centred on the best fit of the rate to a single trajectory, with the error bars giving a 95% confidence interval on that fit. The red line is the linear fit of the system TOF (3.6 ± 0.3 h^−1^) to the 11 independent kinetic measurements, the red dashed line is the 95% confidence interval of that fit and the black dashed line is the untemplated rate for monomers at 100 nM = 0.012 ± 0.002 nM h^−1^.

The initial TOF metric ignores the effect of competitive product inhibition from the rebinding of free-floating products to the template. To test the inhibition resistance of the different designs, we ran the same experiments but with variable concentrations of preannealed *M*_1_*N*_3_ already present in the reaction mix. The results for primary toeholds of length 5–7 nt and handholds of 8–10 nt are plotted in Fig. 3a. From the initial TOFs of the resultant kinetics, we estimated the product concentration at which the reaction’s initial rate is halved (IC_50_) as a metric to compare product inhibition^44^. The results demonstrate the expected inverse correlation between product inhibition resistance and domain lengths, with the estimated IC_50_ of 11 nM for 6t/8h, 4 nM for 6t/9h and 2 nM for 6t/10h (Fig. 3b). We, therefore, select 6t/8h as our optimal design; we prefer 6t/8h relative to 6t/9h specifically due to the emphasis on suppressing product inhibition in this work, and an IC_50_ similar to the monomer concentrations indicates relatively weak, non-cooperative binding to the template by the product. A screening for all *u*t/*v*h conditions and their extracted TOF values are shown in Supplementary Fig. 15 and Supplementary Table 18, respectively.

To test whether the inhibition experienced by 6t/8h is strictly competitive (arising from the competition between *M*_1_*N*_3_ and *M*_1_*L* for binding to the template), we increased the initial concentration of monomers and products by an order of magnitude and catalysed the reaction with 2.5, 5 or 10 nM of template. The three tested conditions show that the IC_50_ is indeed independent of ${[{T}_{xy}]}_{0}$ and remains comparable with the concentration of the monomer (~100 nM) (Supplementary Fig. 16 and Supplementary Table 19).

A catalyst must not only act rapidly but must also be able to complete several catalytic cycles. We report catalytic turnover of the 6t/8h design using a very large monomer:template concentration ratio in Fig. 3c. The single templates can catalyse the assembly of at least 20–25 dimers per template. Moreover, the underlying leak rate in the absence of a template is very slow. The template-free control is consistent with a reaction rate of 0.32 ± 0.06 M^−1^ s^−1^ (Supplementary Table 16 and Supplementary Fig. 12), slower than previous measurements of toehold-free strand displacement^34^. The dimer production signal due to the presence of templates far exceeds this leak reaction, even when templates are only present at a ratio of 1:400 with the monomers. In addition, these experiments confirm that the reaction’s initial rate is proportional to the template concentration (fitted TOF for 100 nM monomers regime of 3.6 ± 0.3 h^−1^) (Fig. 3d).

### Sequence-specific copying by templated dimerization

**Fig. 4:**
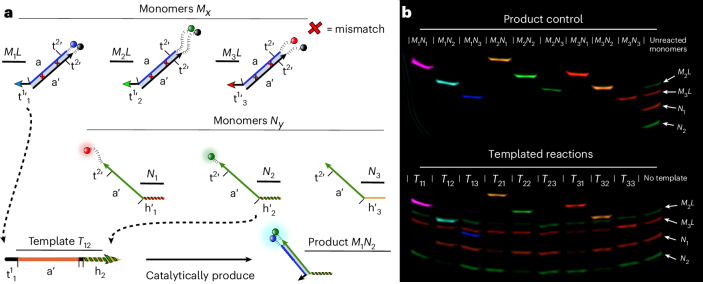
Information propagation by sequence-specific catalytic dimerization. **a**, The design of monomers to demonstrate accurate information propagation in catalytic dimerization. We consider three types of *M*_*x*_, differentiated by their primary toehold, and three types of *N*_*y*_, differentiated by their handholds. Fluorescent labelling, using poly(T) linkers of variable length, allows the identification of all *M*_*x*_*N*_*y*_ complexes through gel electrophoresis. Templates *T*_*xy*_ are intended to selectively template the formation of *M*_*x*_*N*_*y*_ from a pool of all six monomer species. **b**, The fluorescent scan of gel electrophoresis demonstrating sequence-specific templating. Products control: the signal produced by each possible *M*_*x*_*N*_*y*_ dimer produced by annealing 75 nM of each monomer. Templated reactions, the reaction mixture in which a low concentration of a single *T*_*xy*_ (5 nM) is combined with 100 nM of each *M*_*x*_*L* monomer and 75 nM of each *N*_*y*_ monomer. The observed products and signal from unreacted monomers in each well after 40 h of reaction is consistent with the intended *M*_*x*_*N*_*y*_ production. The false colours include: blue, Alexa 488; green, Alexa 546; red, Alexa 647; cyan, FRET 488/546; yellow, FRET 546/648; purple, FRET 488/648.

To demonstrate that the optimized 6t/8h design can perform information propagation by sequence-specific templating, we now consider mixtures with three distinct species per monomer type: *M*_*x*_, *N*_*y*_ with *x*,*y* = 1, 2, 3. The monomers of the same type share the same dimerization domain but have different template recognition domain sequences and different flourophore labels (Fig. 4a and Supplementary Note 2). Thus, there are nine possible *M*_*x*_*N*_*y*_ of similar stability, associated with nine catalytic templates *T*_*xy*_. If each *T*_*xy*_ templates the formation of only the dimer *M*_*x*_*N*_*y*_ from a mixture of monomers, it will have successfully copied its sequence information. The individual characterization of the nine separate templated reactions is given in Supplementary Note 7.2. We evaluate sequence-specific templating both by real-time fluorescence monitoring and by post hoc gel electrophoresis.

**Fig. 5:**
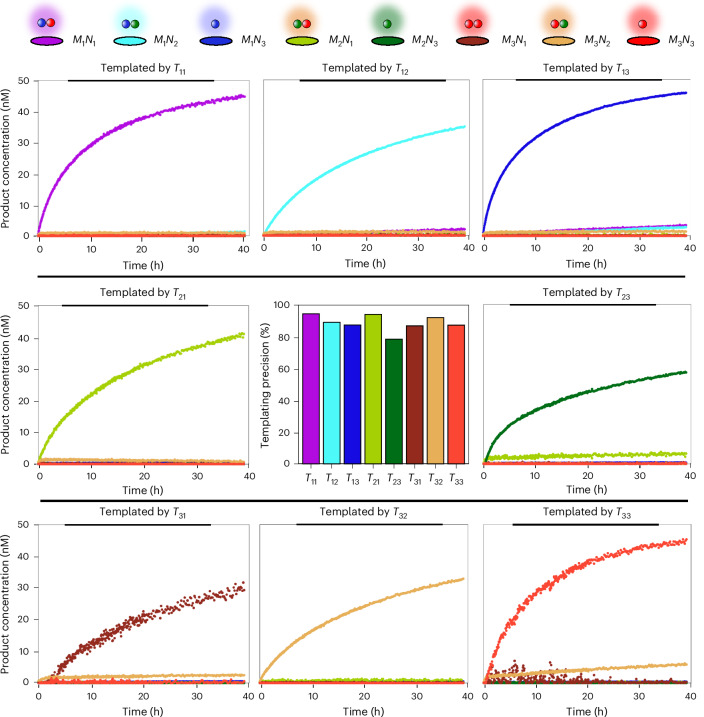
Real-time kinetics of dimer formation for sequence-specific dimerization. A mixture of six monomers and a specific template *T*_*xy*_ were mixed together to react. We plot inferred concentrations of eight of the nine possible dimers for all templates except *T*_22_ (*M*_2_*N*_2_ is not distinguishable from its constituent monomers via fluorescence alone). Note that the PAGE results in Fig. 4b show that *M*_2_*N*_2_ does indeed form as intended. Top: each dimer is represented by the same colour in each plot, indicated by the key. Middle: an estimate of the percentage of correct dimer formation after 24 h for each template.

Figures 4b and 5 illustrate the results of experiments in which a single *T*_*xy*_ is mixed with all six monomer species. In Fig. 4b, we show polyacrylamide gel electrophoresis (PAGE) analysis of the system after 40 h of reaction; the gels demonstrate that information is accurately copied from template to product, with minor variability in speed and specificity probably resulting from differences in recognition sequences and the effects of different fluorescent labels. For each *T*_*xy*_, the expected *M*_*x*_*N*_*y*_ band is visible, and its constituent monomer bands faded, with little evidence of unintended product formation.

The gel results are supported by the inferred concentrations from the real-time kinetics (Fig. 5). We inferred the concentrations of all products—except *M*_2_*N*_2_ due to its low signal-to-noise ratio—by deconvoluting fluorescence signals as described in Supplementary Note 5.2. A quantitative analysis of the kinetic data suggests an accuracy between 80–95% for each template product, despite the challenge of inferring product concentrations through fluorescence alone (Supplementary Results 2 and Supplementary Tables 21–23). Assuming the production of *M*_2_*N*_2_ is similar to other species, as suggested by the PAGE data, we estimate that the mutual information between a uniform distribution of templates and the resultant products would be 2.45 bits, out of a maximum of 3.17 bits for perfect copying. The results for different reactant concentrations are collected in Supplementary Results 2.

**Fig. 6:**
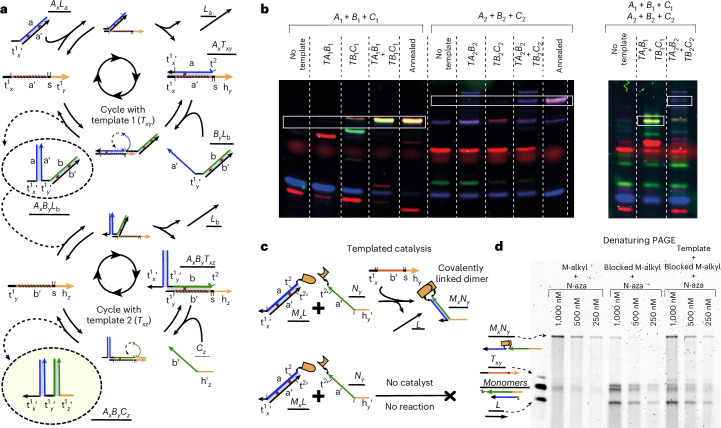
Extension of templating to trimerization and covalent bond formation. **a**, A schematic of a trimerization process *A*_*x*_ + *B*_*y*_ + *C*_*z*_ → *ABC*_*xyz*_ templated by two dimerization catalysts ${T}_{A{B}_{xy}}$ and ${T}_{B{C}_{yz}}$. Each stage is analogous to the catalytic dimerization cycles of Fig. 1. For simplicity, in this diagram, we have assumed template 1 first joins *A*_*x*_ and *B*_*y*_ before template 2 joins *C*_*z*_ to *A*_*x*_*B*_*y*_. **b**, A non-denaturing PAGE analysis of reaction products after mixing either *A*_1_, *B*_1_ and *C*_1_; *A*_2_, *B*_2_ and *C*_2_; or both, with various combinations of templates. The formation of intended products is minimal in the absence of the relevant templates but visible when the templates are present. The bands, including intermediates and unintended products, can be identified by comparing the fluorescence in three channels and migration speed to controls (Supplementary Results 5 and Supplementary Figs. 33–35). **c**, A schematic illustrating the coupling of HMSD-based dimerization to the formation of a covalently linked dimer. The moieties for a click reaction (Cu-catalysed alkyne azide cycloaddition) are conjugated to monomers *M*_1_ and *N*_1_. **d**, Denaturing PAGE, which disrupts the duplex formed by HMSD, is used to detect which systems have formed covalent bonds. The monomers are converted to dimers after hybridization of *M*_1_ an *N*_1_ (‘M-alkyl + N-aza’) but not if binding is inhibited by the presence of lock strands (‘blocked M-alkyl + N-aza’). The action of a template *T*_11_ (at a ratio of 1:10 with *M*_1_*L*) during 24 h restores dimerization. Top: the labels indicate the initial concentrations of *M*_1_*L* and *N*_1_.

The dimerization products are not inert and can participate in downstream processes. The ‘associative toehold’^45^ present in *M*_*x*_*N*_*y*_ allows it to couple to essentially any DNA strand displacement network, as shown when non-catalytically produced *M*_*x*_*N*_*y*_ dimers were used to trigger reporters in ref. ^38^. Here, we show that dimers formed by templating can participate in a subsequent templating reaction, leading to trimer assembly. To build such a system (Fig. 6a), we consider monomers *A*_*x*_, *B*_*y*_ and *C*_*z*_, with *x*,*y*,*z* = 1, 2 giving the recognition sequence identity. *A*_*x*_ and *C*_*z*_ have a similar form to *M*_*x*_ and *N*_*y*_ presented previously, and *B*_*y*_ molecules possess two dimerization domains, allowing the formation of a trimer. *A*_*x*_ and *B*_*y*_ are initially bound to lock strands, inhibiting template-free association. We show in Fig. 6b that two pairs of templates can catalyse the assembly of two distinct species of trimer, *ABC*_111_ and *ABC*_222_, from solutions of (*A*_1_*L*_*A*_, *B*_1_*L*_*B*_, *C*_1_) and (*A*_2_*L*_*A*_, *B*_2_*L*_*B*_, *C*_2_), respectively. Although the system was not optimized to avoid cross-reactivity, we also show that the products *ABC*_111_ and *ABC*_222_ are produced from a pool of all monomers only when the appropriate templates are added, albeit with some yield of intermediates and unintended products.

Biological templating typically uses non-covalent recognition interactions to template the formation of covalent bonds. In our default system, non-covalent interactions template the formation of a non-covalent bond held together by DNA base pairs. However, by adding functional groups that can undergo copper-catalysed alkyne azide cycloaddition to the end of monomers *M*_1_ and *N*_1_ (Fig. 6c), we are able to demonstrate the catalytic dimerization of a covalently linked product. Denaturing PAGE in Fig. 6d shows the formation of covalently linked product is negligible when the monomers are free in solution. However, covalent bond formation occurs once *M*_1_ and *N*_1_ have undergone dimerization via the HMSD method introduced here, even if template *T*_11_ is present at a relatively low concentration.

## Discussion

Using our DNA-based dimerization system, which channels the free energy of dimerization into disrupting binding to a template, we demonstrated: (1) weak competitive product inhibition, (2) a catalytic reaction around 1,500 times faster than its leak rate under the conditions considered (5 nM template, 100 nM monomers), (3) a turnover of at least 20–25 reactions per template, (4) information propagation by highly specific molecular templating, with an accuracy of around 90% when selecting a single product from nine alternatives, (5) the incorporation of a templating reaction into a larger network and (6) templated covalent bond formation.

Comparing the performance of our DNA-based system with other synthetic, sequence-specific templating mechanisms is not straightforward, since the capabilities of those systems are often couched in the language of autocatalysis and self-replication. However, early biomolecular replication experiments^16,22,23,36^ typically show catalytic rates only a few times faster than spontaneous reactions, substantial product inhibition at low product concentrations or limited turnover per catalyst. More recent work^17,19,37^ has demonstrated improvements along various axes of this performance space. However, unlike previous designs, our HMSD-templating system is, in principle, extendable to longer templates. Crucially, the binding of *M*_*x*_ to the template is stable (Supplementary Results 4 and Supplementary Fig. 32) until that binding is disrupted by dimerization. Our template for dimerization can therefore be extended by adding more sites that look like the binding site of *M*_*x*_, and copies could grow while remaining template-attached until they reach a final truncated binding site analogous to the binding site for monomer *N*_*y*_ in this work (Supplementary Note 8 and Supplementary Fig. 19). Recent theoretical work has demonstrated that a system of this kind can produce copies of longer templates^7^.

A major challenge when engineering catalytically controlled systems is that the reaction thermodynamics needs to be finely balanced,^46^ and it is difficult to engineer large thermodynamic driving forces without triggering unwanted leak reactions^42^. The relatively weak thermodynamic driving used here (the elimination of two mismatched base pairs) may limit yield in, for example, Fig. 5. In addition, it is challenging to ensure that the products are sufficiently metastable that slow product interconversion does not occur; we investigate this behaviour for the product *M*_1_*N*_3_ in Supplementary Results 3. Our work emphasises the importance in resolving these challenges when building templating systems.

The fundamental advantage of catalytic molecular templating is that a set of monomers can be combined into a combinatorially large number of products, with only a small amount of template required for a high yield. With dimerization, a small concentration of template can be added to a single master mixture of *N* different ingredients to produce a large amount of any one of *O*(*N*^2^) different products. For a template of length *L*, *O*(*N*^*L*^) products are possible. Catalytic templating therefore offers the potential to reduce the costs of assembling a diverse set of structures^47^ and facilitate combinatorial screening^15,48–50^, as each new product can use the same pool of monomers and requires only the introduction of a small amount of novel template.

The most promising applications of our work exploit these properties. First, the motif introduced here can be immediately coupled to conventional DNA-based strand displacement circuits, which have applications in diagnostics, molecular biophysics and unconventional computing^31–33^. In such circuits, our catalytic templating mechanism provides a simple way to amplify *O*(*N*^2^) different signals using only *O*(*N*) signal-processing components. Generalizing to longer templates—as proposed in Supplementary Note 8– would allow exponentially more signals at a linear cost. A simpler (albeit less powerful) alternative would be to generalize the mechanism in Fig. 6b to allow a series of dimerization catalysts to select specific products from a large ensemble.

Second, the DNA strands whose assembly we have templated are functionalized with fluorophores, resulting in assembly dependent fluorescence. Instead, they could be functionalized with other chemical groups that could perform some downstream task, such as selective binding, catalysis or assembly into large structures such as gels^51^ with properties determined by the templated sequence. A long term vision would be to assemble labelled oligomers that could then fold into a minimal synthetic analogue of a protein.

One can also use DNA to template covalent interactions directly between the functional groups themselves, with a view to generating combinatorial libraries of biologically, medically or chemically functional small molecules^5,10,11,14,15^. Here, we show the ability to template click reactions with high catalytic turnover, typically a major challenge in DNA-based templating of covalent chemistry^37^. The next steps include: incorporating other chemistries and going beyond dimerization to the formation of longer products.

An alternative to using DNA to template the assembly of organic molecules would be to use the organic molecules as templates themselves. Our work illustrates the principle that channelling of free energy into the disruption of template bonds is a viable mechanism for information propagation by catalytic assembly of molecules. An important question is whether this mechanism can be translated into other chemistries. Recent work has promisingly shown that a similar principle can be used to template tetrameric organic molecules, albeit without sequence specificity^40^.

## Methods

### System design principles

Since the product *M*_*x*_*N*_*y*_ must detach from the template *T*_*xy*_, the overall reaction simply replaces a duplex in the *M*_*x*_*L* complex with an identical one in *M*_*x*_*N*_*y*_, with no extra base pairs formed in the process. As stated, the process would not have any thermodynamic drive pushing it towards dimerization. Therefore, to make the *M*_*x*_*N*_*y*_ products more stable than the reactants, without substantially increasing the rate of template-free dimerization, we incorporate two mismatched base pairs in the *M*_*x*_*L* duplexes^42^. Each of these sequence mismatches destabilizes the *M*_*x*_*L* duplex by around 9 *k*_B_*T* relative to the *M*_*x*_*N*_*y*_ product^21^. One of the mismatches is eliminated during the TMSD reaction and the second during HMSD (Fig. 1d).

Based on a previous study^38^, we introduced a secondary toehold of 2 nt, as it ensures fast HMSD while triggering negligible TMSD on its own. In addition, the final design of our system uses a dimerization domain as illustrated in Fig. 1d. This design has ‘clamp’ base pairs in *M*_*x*_*L*, not present in *M*_*x*_*N*_*y*_, to reduce the spontaneous displacement of *L* from *M*_*x*_*L* by *N*_*y*_. We collect the results for an alternative design without the clamps in Supplementary Results 1 (Supplementary Figs. 20 and 21); this alternative design was slightly faster but suffered from a high dimerization rate in the absence of *T*_*xy*_.

To identify the possible *M*_*x*_*N*_*y*_ dimers when performing sequence-specific dimerization, we label both *M*_*x*_ and *N*_*y*_ with fluorophores, as shown in Fig. 4a. As this labelling alone cannot unmistakably distinguish all nine complexes, we also give each monomer different lengths for the poly(T) linkers connecting the fluorophores to the monomers. The different linkers allow dimers to be identified during gel electrophoresis by a combination of their migration speed and strength of Förster resonance energy transfer (FRET).

### DNA sequence design

DNA sequences that satisfy the principles outlined above and minimize undesired interactions during HMSD were designed with bespoke scripts using the NUPACK server (http://www.nupack.org)^52,53^. All strands were purchased from Integrated DNA Technologies with high-performance liquid chromatography purification and normalized at 100 μM in LabReady buffer. All sequences are listed by function in Supplementary Note 2 and Supplementary Tables 1–5.

### Complex preparation

#### *M*_*x*_*L* duplex preparation for non-covalent dimerization

The *M*_*x*_*L* duplexes were annealed at a concentration of 2 μM of *M*_*x*_ with a 10% excess of its corresponding *L* to ensure the sequestration of every *M*_*x*_. Annealing was performed in 100 μL of experimental buffer (Tris/acetate/EDTA 1× and 1 M NaCl, pH 8.3) and annealed by heating to 95 °C for 4 min and cooling to 20 °C at a rate of 1 °C min^−1^.

#### Complex preparation for trimerization

Trimerization monomers *A*_*x*_*L* and *B*_*x*_*L* were annealed at a concentration of 200 nM of each monomer with a twofold excess of its corresponding *L*. The total volume of 100 μl of the strands in experimental buffer was subjected to an annealing program identical to *M*_*x*_*L* for dimerization.

#### Complex preparation for covalent dimerization

The monomer *M*_1-alk_*L* was annealed at a concentration of 2.5 μM with a 50% excess of its corresponding *L*. The complexes were annealed as described for non-covalent dimerization. The buffer used for annealing was 100 mM NaH_2_PO_4_ buffer containing 200 mM NaCl.

### Bulk fluorescence spectroscopy

Bulk fluorescence assays were carried out in a Clariostar Microplate reader (BMG LABTECH) using flat μClear bottom 96-well plates (Greiner) and reading from the bottom. The experimental protocols were based on those previously described in ref. ^38^. Each experiment consisted of the system’s kinetics and a set of complementary measurements. These complementary measurements quantified a fluorescence baseline and estimated the concentration of each species in the system from the fluorescence signal measured after sequentially triggering the reaction of all the species. More detailed protocols for the different experiments performed in this work are provided in Supplementary Note 4 (Supplementary Tables 9–14 and Supplementary Figs. 5–8). The unprocessed fluorescence signals are available in ref. ^54^ (see ‘Data availability’ and ‘Code availability’ sections).

#### Tests for optimizing the dimerization mechanism

The main tests on the kinetics of the dimerization mechanism were performed using the monomers *M*_1_ and *N*_3_ and the template *T*_13_. Unless stated otherwise, the kinetic results were obtained by tracking the fluorescence of the labelled strand *M*_1_ at 25 °C in a 200 μl volume. Typically, this fluorescence would increase due to the displacement of the quencher-bearing lock strand *L* in the presence of *T*_13_ and *N*_3_. Although we do not directly monitor *M*_1_*N*_3_ during these experiments, Fig. 2b demonstrates that *N*_3_ is required, as well as *T*_13_, for sustained *M*_1_*L* turnover, and we provide results for the kinetics of individual TMSD and HMSD steps in Supplementary Note 7.1. Product formation is monitored directly in subsequent experiments assessing catalytic specificity (Figs. 4 and 5).

The kinetics were recorded after injecting 50 μl of the reaction triggering species into 150 μl of experimental buffer containing the rest of the reactant species (pump speed 430 μl s^−1^). The final mixture was shaken for 3 s (double orbital, at 400 rpm). The injected and reacting volumes were previously preheated to the experiment temperature. Simultaneously, we recorded the fluorescence of a positive control (*M*_1_*N*_3_) and a negative control (experimental buffer). These controls were used to correct the measured fluorescence due to temperature and volume changes. The samples were contained in Eppendorf Lobind tubes, and the plate reader’s injector system was passivated by incubating with bovine serum albumin at 5% weight per volume for 30 min to maximize the concentration reproducibility during the assays^55^.

Strand *M*_1_ was labelled with Alexa Fluor 488 (excitation: 488/14 nm, emission: 535/30 nm) and strand *L* with FQ IowaBlack quencher. Every tested system was assayed at least three times, including modifications of either *T*_13_ or *N*_3_ concentrations to extract further information from the reaction kinetics in different regimes. The fluorescence signal was averaged for 100 flashes in a spiral area scan per data point. For experiments that lasted under 1 h, the kinetics were just averaged for 20 flashes per data point. The data from further experiments conducted during optimization, including assays on individual reaction substeps, are reported in Supplementary Note 7 (Supplementary Tables 15–20 and Supplementary Figs. 10–18).

Once the basic design had been optimized through tests of variants of *M*_1_, *N*_3_ and *T*_13_, we also measured the dimerization kinetics of other combinations of *M*_*x*_, *N*_*y*_ and *T*_*xy*_ for this optimized design. The results of these experiments are reported in Supplementary Note 7 (Supplementary Tables 15, 16 and 20 and Supplementary Figs. 11, 12, 17 and 18).

The additional monomer species used during these assays were labelled in the following way: *M*_2_*L*: Alexa Fluor 546 (excitation: 540/20 nm, emission: 590/30 nm) and Black Hole quencher-2; *M*_3_*L*: Alexa Fluor 647 (excitation: 625/30 nm, emission: 680/30 nm) and RQ IowaBlack quencher; *N*_1_: Alexa Fluor 647; *N*_2_: Alexa Fluor 546. To monitor as many species as possible, the experiments recorded these fluorescence signals and FRET resulting from the combinations of Alexa Fluor 488/46 (excitation: 488/14 nm, emission: 590/30 nm), Alexa Fluor 488/47 (excitation: 488/14 nm, emission: 670/30 nm and Alexa Fluor 546/47 (excitation: 540/20 nm, emission: 680/30 nm).

#### Kinetics of templated sequence-specific copying

The templating of specific products required more sophisticated bulk fluorescence assays. The experiment reported in Fig. 5 consisted of ten wells loaded with an intended concentration of 100 nM of each monomer *M* (*M*_1_*L*, *M*_2_*L* and *M*_3_*L*). Nine of these wells also contained 5 nM of one of the nine tested templates, with the tenth containing no template to track the mechanism’s leak reaction. The reaction in each of these wells was triggered with 50 μl of a solution of the *N* monomers (*N*_1_, *N*_2_ and *N*_3_), each of them at an intended concentration of 75 nM. The experiment also included another set of ten wells. Nine of them contained positive controls for each dimer, formed by annealing 75 nM of each combination of *M* and *N* strands. The last well was a buffer-only blank control. The raw fluorescence data for this experiment are present in Supplementary Fig. 8.

The sequence-specific copying experiments were initially performed using different concentrations of the monomers and templates (Supplementary Results 2 (Supplementary Figs. 22–29)). The results are qualitatively similar, though an apparent slow conversion of *M*_1_*N*_3_ into *M*_1_*N*_1_ and *M*_1_*N*_2_ was observed when *M*_*x*_*L* was not initially present in excess of *N*_*y*_. It appears that empty templates can also catalyze this interconversion reaction on slower timescales than the templating of assembly, reducing the apparent accuracy. This mechanism is further discussed in Supplementary Results 3 (Supplementary Figs. 30 and 31).

### PAGE

#### Sequence-specific dimerization

After recording the kinetics of sequence-specific copying by templated dimerization, aliquots from the nine template conditions and the no-template control were loaded into a polyacrylamide gel. A second gel was produced as a reference, using the dimers assembled in the kinetics positive control and a freshly made mixture of all unreacted monomers (*M*_*x*_*L* at 100 nM and *N*_*y*_ at 75 nM) in the experimental buffer. The gel used was a precast Novex 10% 37.5:1 acrylamide:Bis-acrylamide gel in Tris/borate/EDTA buffer (TBE) 1× (Invitrogen). The samples were mixed with native gel loading dye solution 10× (Invitrogen), and 15 μl of the mixture was loaded in each well. The gels were run in an X-Cell SureLock electrophoresis chamber, using TBE 1× + 50 mM NaCl as running buffer and a program of 10 V cm^−1^ for 30 min and 15 V cm^−1^ for 90 min. The tank was kept in an ice bath during the electrophoresis to avoid sample heating. To avoid band distortions due to the difference in ionic strength between buffer (50 mM NaCl) and samples (900 mM NaCl), the loaded samples were left in the wells for at least 30 min before starting the electrophoresis^56^.

The gels were imaged with a Typhoon gel scanner (Amersham). The false colours reported in Fig. 4b correspond to the following fluorescence measurements: blue (excitation: 488 nm, emission: 525/20 nm), green (excitation: 532 nm, emission: 570/20 nm), red (excitation: 635 nm, emission: 670/30 nm), cyan (excitation: 488 nm, emission: 570/20 nm), yellow (excitation: 532 nm, emission: 670/30 nm) and purple (excitation: 488 nm, emission: 670/30 nm). The scanner gain was automatically optimized for each scan before imaging. The results reported in Fig. 4b and Supplementary Figs. 23 and 26 are an overlay of the six scans, represented individually in Supplementary Figs. 24, 27 and 29. The scans were merged using ImageJ after redefining the brightness adjustment range of the image to span from 10–100%, excluding the lower 0–10% range. This adjustment removed the background produced by the loading dye and the gel matrix in some channels, without removing any monomer or correct and incorrect product bands. The raw data for each scan are contained in ref. ^54^ (see ‘Data availability’ and ‘Code availability’ sections).

#### Trimer formation

The experiment reported in Fig. 6b consisted of several aliquots of 200 μl with 10 nM of each monomer (either *A*_1_*L* + *B*_1_*L* + *C*_1_, *A*_2_*L* + *B*_2_*L* + *C*_2_ or both sets). Where specified, the reaction volume also contained 2 nM of up to two different templates. The samples were incubated at 30 °C for 48 h.

Electrophoresis was performed in similar conditions to the sequence-specific dimerization. The main differences were the volume loaded, 5 μl + 2 μl of native gel purple loading dye solution 6× and the electrophoresis program: 11.25 V cm^−1^ for 90 min in TBE 1×. No ice bath was used due to the lower voltage and ionic strength of the running buffer.

The results reported in Fig. 6b are an overlay of three fluorescence scans (blue: 488 nm laser excitation, 525BP20 emission filter; green: 532 nm laser excitation, 570BP20 emission filter; red: 635 nm laser excitation, 670BP20 emission filter). Due to the lower quantity of DNA loaded in the gel, the brightness range had to be adjusted in ImageJ to span from 0–20%, to clearly observe the bands. The individual channels in pseudo-colour along with their composite are shown in Supplementary Figs. 33 and 34, and the raw data for each scan are contained in ref. ^54^ (see ‘Data availability’ and ‘Code availability’ sections).

The identity of the bands is inferred by comparing them to controls (Supplementary Fig. 35). For the controls, the relevant strands were directly annealed at 100 μl volume in experimental buffer (1× Tris/acetate/EDTA, 1 M NaCl, pH 8) with 200–400 nM strand concentrations by heating the solution at 95 °C for 5 min and then cooling it to 20 °C at a rate of 0.5 °C min^−1^.

#### Covalent dimerization

The experiment reported in Fig. 6d consists of annealed *M*_1-alk_*L*, mixed with *N*_1-aza_. Another aliquot was mixed in the absence of *L* strand and another in the presence of *T*_11_. The final concentrations used were: for [*M*_1-alk_*L*], 2,000 nM; [*L*], 1,000 nM; [*N*_1-aza_], 3,000 nM; and [*T*_11_], 200 nM. The three DNA aliquots were each diluted 2×, 4× and 6× ([*M*_1-alk_]: 1,000, 500 and 250 nM) to a total volume of 45 μl in the 100 mM NaH_2_PO_4_ buffer. A total of 1.5 μl of a Cu-premix was added to each of the nine 45 μl DNA aliquots. The Cu-premix was prepared by mixing 100 mM aqueous solutions of CuSO_4_ and tris(3-hydroxypropyltriazolylmethyl)amine (THPTA), with a proportion 1:2 and preincubating for 15 min. Afterwards, 1 μl of a 100 mM solution of ascorbic acid was added to each DNA aliquot, and they were incubated at 25 °C for 24 h.

After incubation, 3 μl of each sample was mixed with 3 μl of 1–2× Gel Loading Buffer II (Invitrogen), and they were incubated at 95 °C for 5 min. Denaturing PAGE was performed by loading 4 μl of the nine samples in a precast Novex 10% 37.5:1 acrylamide:Bis-acrylamide gel in 10% *tert*-butyl (Invitrogen) and running them at 22.5 V cm^−1^ for 40 min in 1× TBE buffer at a temperature of 65 °C. The 10% *tert*-butyl polyacrylamide gel was prerun at 6.25 V cm^−1^ for 30 min. After electrophoresis the gel was stained for 30 min in TBE 1× buffer containing 1× SYBR Gold. The gel was imaged with the Typhoon gel scanner (excitation: 488 nm, emission: 525/20 nm). The raw data for each scan are contained in ref. ^54^ (see ‘Data availability’ and ‘Code availability’ sections).

### Calibration of fluorescent signals for real-time kinetics

Fluorescence calibrations were made for all the fluorescence species used in this work. The calibrations aimed to estimate the units of fluorescence produced per nanomolar of each complex containing a fluorophore-labelled species to quantify its concentration during the experiments. The calibration curves ranged from 15 to 150 nM, in 200 μl volumes, from stock solutions normalized at 100 μM. Additional calibrations tested the variation of fluorescence of *M*_1_ when bound to the template. The calibration protocol and results are given in Supplementary Note 3 (Supplementary Figs. 1–4 and Supplementary Tables 7 and 8).

### Data processing

The data from bulk fluorescence experiments were corrected with each experiment’s positive and negative controls and transformed from fluorescence units to concentrations of the relevant species using fluorescence calibrations. The transformation procedures are described in detail in Supplementary Note 5 (Supplementary Fig. 9), and the scripts are available at ref. ^54^ (see ‘Data availability’ and ‘Code availability’ sections).

### Data fitting

Simple models of reaction kinetics (Supplementary Note 6) were used to fit reaction rate constants to the processed data to give more information about system performance. In particular, we estimated the following quantities: the rate constant for binding of *M*_*x*_*L* to *T*_*xy*_ (or the displacement of *L* from *M*_*x*_ by *T*_*xy*_), the rate constant for spontaneous leak reactions between *M*_*x*_*L* and *N*_*y*_, the rate constant of the HMSD substep for *M*_1_*N*_3_ and the initial reaction rate for catalytic dimerization (TOF) for all templates used.

All fits were performed with MATLAB R2019a Optimization Toolbox. Supplementary Note 7 contains a detailed description of the fitting procedures, with the resultant fits tabulated in Supplementary Tables 15–20 and illustrated in Supplementary Figs. 10–18.

## Online content

Any methods, additional references, Nature Portfolio reporting summaries, source data, extended data, supplementary information, acknowledgements, peer review information; details of author contributions and competing interests; and statements of data and code availability are available at 10.1038/s41557-025-01831-x.

## Supplementary information

Supplementary Information Supplementary Figs. 1–35, Tables 1–23, Notes 1–8 and Results 1–5.

## Data Availability

Further results, and information on experimental methods and data processing, can be obtained from the Supplementary Information. Raw data, figures and the scripts used to process that data and generate the results plotted here are available via Zenodo at 10.5281/zenodo.14556331 (ref. ^54^).

## Code availability

All scripts used to process the data and generate the results plotted here are available via Zenodo at 10.5281/zenodo.14556331 (ref. ^54^).

## References

[1] Brocchieri, L. & Karlin, S. Protein length in eukaryotic and prokaryotic proteomes. *Nucl. Acids Res.* **33**, 3390 (2005). 15951512 10.1093/nar/gki615 PMC1150220

[2] Sartori, P. & Leibler, S. Lessons from equilibrium statistical physics regarding the assembly of protein complexes. *Proc. Natl Acad. Sci. USA* **117**, 114–120 (2020). 31871201 10.1073/pnas.1911028117 PMC6955335

[3] Crick, F. Central dogma of molecular biology. *Nature* **227**, 561–563 (1970). 4913914 10.1038/227561a0

[4] Anfinsen, C. B. Principles that govern the folding of protein chains. *Science* **181**, 223–230 (1973). 4124164 10.1126/science.181.4096.223

[5] Michaelis, J., Roloff, A. & Seitz, O. Amplification by nucleic acid-templated reactions. *Org. Biomol. Chem.* **12**, 2821–2833 (2014). 24671414 10.1039/c4ob00096j

[6] Orgel, L. E. Molecular replication. *Nature* **358**, 203–209 (1992). 1630488 10.1038/358203a0

[7] Juritz, J., Poulton, J. M. & Ouldridge, T. E. Minimal mechanism for cyclic templating of length-controlled copolymers under isothermal conditions. *J. Chem. Phys.* **156**, 074103 (2022). 35183080 10.1063/5.0077865

[8] Poulton, J. M. & Ouldridge, T. E. Edge-effects dominate copying thermodynamics for finite-length molecular oligomers. *New J. Phys.* **23**, 63061 (2021).

[9] Samokhvalova, S. & Lutz, J.-F. Macromolecular information transfer. *Angew. Chem.* **135**, 202300014 (2023). 10.1002/anie.202300014 36696359

[10] Di Pisa, M. & Seitz, O. Nucleic acid templated reactions for chemical biology. *ChemMedChem* **12**, 872–882 (2017). 28480997 10.1002/cmdc.201700266 PMC5488204

[11] Gorska, K. & Winssinger, N. Reactions templated by nucleic acids: more ways to translate oligonucleotide-based instructions into emerging function. *Angew. Chem. Int. Ed.* **52**, 6820–6843 (2013). 10.1002/anie.201208460 23794204

[12] Lutz, J. F., Ouchi, M., Liu, D. R. & Sawamoto, M. Sequence-controlled polymers. *Science* **341**, 628 (2013). 10.1126/science.1238149 23929982

[13] De Neve, J., Haven, J. J., Maes, L. & Junkers, T. Sequence-definition from controlled polymerization: the next generation of materials. *Polym. Chem.* **9**, 4692–4705 (2018).

[14] O’Reilly, R. K., Turberfield, A. J. & Wilks, T. R. The evolution of DNA-templated synthesis as a tool for materials discovery. *Acc. Chem. Res.* **50**, 2496–2509 (2017). 28915003 10.1021/acs.accounts.7b00280 PMC5746846

[15] Usanov, D. L., Chan, A. I., Maianti, J. P. & Liu, D. R. Second-generation DNA-templated macrocycle libraries for the discovery of bioactive small molecules. *Nat. Chem.* **10**, 704–714 (2018). 29610462 10.1038/s41557-018-0033-8 PMC6014893

[16] Vidonne, A. & Philp, D. Making molecules make themselves—the chemistry of artificial replicators. *Eur. J. Organ. Chem.* **2009**, 593–610 (2009).

[17] Robertson, M. P. & Joyce, G. F. Highly efficient self-replicating RNA enzymes. *Chem. Biol.* **21**, 238–245 (2014). 24388759 10.1016/j.chembiol.2013.12.004 PMC3943892

[18] Kreysing, M., Keil, L., Lanzmich, S. & Braun, D. Heat flux across an open pore enables the continuous replication and selection of oligonucleotides towards increasing length. *Nat. Chem.* **7**, 203–208 (2015). 25698328 10.1038/nchem.2155

[19] Kosikova, T. & Philp, D. Two synthetic replicators compete to process a dynamic reagent pool. *J. Am. Chem. Soc.* **141**, 3059–3072 (2019). 30668914 10.1021/jacs.8b12077

[20] Walter, C. *The Use of Product Inhibition and Other Kinetic Methods in the Determination of Mechanisms of Enzyme Action* 645–724 (Wiley, 1964); 10.1002/9780470143537.ch18

[21] SantaLucia, J. & Hicks, D. The thermodynamics of DNA structural motifs. *Annu. Rev. Biophys. Biomol. Struct.* **33**, 415–440 (2004). 15139820 10.1146/annurev.biophys.32.110601.141800

[22] Sievers, D. & Von Kiedrowski, G. Self-replication of complementary nucleotide-based oligomers. *Nature* **369**, 221–224 (1994). 8183342 10.1038/369221a0

[23] Lincoln, T. A. & Joyce, G. F. Self-sustained replication of an RNA enzyme. *Science* **323**, 1229–1232 (2009). 19131595 10.1126/science.1167856 PMC2652413

[24] Rosenberger, J. H. et al. Heat flux across an open pore enables the continuous replication and selection of oligonucleotides towards increasing length. *Phys. Rev. X* **11**, 031055 (2021). 10.1038/nchem.2155 25698328

[25] He, X. et al. Exponential growth and selection in self-replicating materials from DNA origami rafts. *Nat. Mater.* **16**, 993–997 (2017). 28920942 10.1038/nmat4986

[26] Núñez-Villanueva, D. & Hunter, C. A. Molecular replication using covalent base-pairs with traceless linkers. *Org. Biomol. Chem.* **17**, 9660–9665 (2019). 31691702 10.1039/c9ob02336d

[27] Schulman, R., Yurke, B. & Winfree, E. Robust self-replication of combinatorial information via crystal growth and scission. *Proc. Natl Acad. Sci. USA* **109**, 6405–6410 (2012). 22493232 10.1073/pnas.1117813109 PMC3340064

[28] Kuehnlein, A., Lanzmich, S. A. & Braun, D. tRNA sequences can assemble into a replicator. *eLife*10.7554/eLife.63431 (2021). 10.7554/eLife.63431 PMC7924937 33648631

[29] Colomb-Delsuc, M., Mattia, E., Sadownik, J. W. & Otto, S. Exponential self-replication enabled through a fibre elongation/breakage mechanism. *Nat. Commun.* **6**, 7427 (2015). 26081104 10.1038/ncomms8427 PMC4557357

[30] Monreal Santiago, G., Liu, K., Browne, W. R. & Otto, S. Emergence of light-driven protometabolism on recruitment of a photocatalytic cofactor by a self-replicator. *Nat. Chem.* **12**, 603–607 (2020). 32591744 10.1038/s41557-020-0494-4

[31] Soloveichik, D., Seelig, G. & Winfree, E. DNA as a universal substrate for chemical kinetics. *Proc. Natl Acad. Sci. USA* **107**, 5393–5398 (2010). 20203007 10.1073/pnas.0909380107 PMC2851759

[32] Chen, Y.-J. et al. Programmable chemical controllers made from DNA. *Nat. Nanotechnol.* **8**, 755–762 (2013). 24077029 10.1038/nnano.2013.189 PMC4150546

[33] Deluca, M., Shi, Z., Castro, C. E. & Arya, G. Dynamic DNA nanotechnology: toward functional nanoscale devices. *Nanoscale Horiz.* **5**, 182–201 (2020).

[34] Zhang, D. Y. & Winfree, E. Control of DNA strand displacement kinetics using toehold exchange. *J. Am. Chem. Soc.* **131**, 17303–17314 (2009). 19894722 10.1021/ja906987s

[35] Robertson, C. C., Kosikova, T. & Philp, D. Encoding multiple reactivity modes within a single synthetic-replicator. *J. Am. Chem. Soc.* **142**, 11139–11152 (2020). 32414236 10.1021/jacs.0c03527

[36] Robertson, A., Sinclair, A. J. & Philp, D. Minimal self-replicating systems. *Chem. Soc. Rev.* **29**, 141–152 (2000).

[37] Roloff, A. & Seitz, O. Reducing product inhibition in nucleic acid-templated ligation reactions: DNA-templated cycligation. *ChemBioChem* **14**, 2322–2328 (2013). 24243697 10.1002/cbic.201300516

[38] Cabello-Garcia, J., Bae, W., Stan V, G.-B. & Ouldridge, T. E. Handhold-mediated strand displacement: a nucleic acid based mechanism for generating far-from-equilibrium assemblies through templated reactions. *ACS Nano* **15**, 3272–3283 (2021). 33470806 10.1021/acsnano.0c10068

[39] Osuna Gálvez, A. & Bode, J. W. Traceless templated amide-forming ligations. *J. Am. Chem. Soc.* **141**, 8721–8726 (2019). 31117658 10.1021/jacs.9b03597

[40] Lewandowski, B. M. et al. Catalytic length-controlled oligomerization with synthetic programmable templates. *Nat. Synth.* **2**, 331–337 (2023).

[41] Mukherjee, R., Sengar, A., Cabello-García, J. & Ouldridge, T. E. Kinetic proofreading can enhance specificity in a nonenzymatic dna strand displacement network. *J. Am. Chem. Soc.* **146**, 18916–18926 (2024). 38951503 10.1021/jacs.3c14673 PMC11258683

[42] Haley, N. E. C. et al. Design of hidden thermodynamic driving for non-equilibrium systems via mismatch elimination during DNA strand displacement. *Nat. Commun.*10.1038/s41467-020-16353-y (2020). 10.1038/s41467-020-16353-y PMC7244503 32444600

[43] Kozuch, S. & Martin, J. M. L. ‘Turning over’ definitions in catalytic cycles. *ACS Catal.* **2**, 2787–2794 (2012).

[44] Swinney, D. C. in *Annual Reports in Medicinal Chemistry* (ed. Macor, J. E. B. T.) Vol. 46, 301–317 (Academic, 2011); 10.1016/B978-0-12-386009-5.00009-6

[45] Chen, X. Expanding the rule set of DNA circuitry with associative toehold activation. *J. Am. Chem. Soc.* **134**, 263–271 (2012). 22129141 10.1021/ja206690a PMC3260326

[46] Deshpande, A. & Ouldridge, T. E. Optimizing enzymatic catalysts for rapid turnover of substrates with low enzyme sequestration. *Biol. Cybern.* **114**, 653–668 (2020). 33044662 10.1007/s00422-020-00846-6

[47] Deacy, A. C., Gregory, G. L., Sulley, G. S., Chen, T. T. D. & Williams, C. K. Sequence control from mixtures: switchable polymerization catalysis and future materials applications. *J. Am. Chem. Soc.* **143**, 10021–10040 (2021). 34190553 10.1021/jacs.1c03250 PMC8297863

[48] Satz, A. L., Brunschweiger, A. & M. E. Flanagan, et al. DNA-encoded chemical libraries. *Nat. Rev. Methods Primers* **2**, 3 (2022).

[49] Petersen, L. K. et al. Novel p38*α* MAP kinase inhibitors identified from yoctoreactor dna-encoded small molecule library. *Med. Chem. Commun.* **7**, 1332–1339 (2016).

[50] Peterson, A. A. & Liu, D. R. Small-molecule discovery through DNA-encoded libraries. *Nat. Rev. Drug. Discov.* **22**, 699–722 (2023). 10.1038/s41573-023-00713-6 PMC10924799 37328653

[51] Feng, Y. & Philp, D. A molecular replication process drives supramolecular polymerization. *J. Am. Chem. Soc.* 17029–17039 (2021). 10.1021/jacs.1c06404 34617739

[52] Zadeh, J. N. et al. NUPACK: analysis and design of nucleic acid systems. *J. Comput. Chem.* **32**, 170–173 (2011). 20645303 10.1002/jcc.21596

[53] Wolfe, B. R., Porubsky, N. J., Zadeh, J. N., Dirks, R. M. & Pierce, N. A. Constrained multistate sequence design for nucleic acid reaction pathway engineering. *J. Am. Chem. Soc.* **139**, 3134–3144 (2017). 28191938 10.1021/jacs.6b12693

[54] Cabello-Garcia, J., Mukherjee, R., Bae, W., Stan V, G. -B. & Ouldridge, T. E. Information propagation through enzyme-free catalytic templating of DNA dimerization with weak product inhibition. *Zenodo*10.5281/zenodo.14556331 (2023). 10.1038/s41557-025-01831-x PMC12313520 40473818

[55] Kanoatov, M. & Krylov, S. N. DNA adsorption to the reservoir walls causing irreproducibility in studies of protein-DNA interactions by methods of kinetic capillary electrophoresis. *Anal. Chem.* **83**, 8041–8045 (2011). 21923122 10.1021/ac202048y

[56] Ha, W. Y., Shaw, P. C. & Wang, J. Improved electrophoretic resolution of DNA fragments in samples containing high concentrations of salts. *Biotechniques* **26**, 425–426 (1999). 10090977 10.2144/99263bm11

