# Supplementary material for: Information propagation through enzyme-free catalytic templating of DNA dimerization with weak product inhibition
Source: Nat Chem. 2025 Jun 5;17(8):1179–87. doi: 10.1038/s41557-025-01831-x (PMC12313520; doi:10.1038/s41557-025-01831-x)
Supplement: Supplementary file 1 — Supplementary Figs. 1–35, Tables 1–23, Notes 1–8 and Results 1–5. [file 41557_2025_1831_MOESM1_ESM.pdf]

nature chemistry

Supplementary information

https://doi.org/10.1038/s41557-025-01831-x

Information propagation through enzyme-free catalytic templating of DNA dimerization with weak product inhibition

In the format provided by the authors and unedited

Supplementary Information for Information propagation through enzyme-free catalytic templating of DNA dimerization with weak product inhibition

Javier Cabello Garcia,† Rakesh Mukherjee,‡ Wooli Bae,¶ Guy-Bart V. Stan,‡ and Thomas E. Ouldridge*,‡

†*Department of Chemistry, University College London, London,WC1H 0AJ, UK*

‡*Department of Bioengineering and Centre for Synthetic Biology, Imperial College London, London, SW7 2AZ, UK*

¶*Department of Physics, University of Surrey, Guildford, GU2 7XH, UK*

E-mail: t.ouldridge@imperial.ac.uk

Supplementary tables

1 Template strands (T_{xy}) 11

2 Monomer M (M_x) and its lock strands (L_x) 12

3 Monomer N (N_y) strands 12

4 Sequences for trimerization experiments 13

5 Additional strands for covalent bond formation 13

6 Strands used in each figure of this publication 14

7 Calibration matrix 1: multi-channel coefficient of fluorescence (fluorescence emission per nM) of the species present in sequence-specific copying experiments at 25° C 19

8 Calibration matrix 2: multi-channel coefficient of fluorescence (fluorescence emission per nM) of the species present in sequence-specific copying experiments at 25° C 20

9 Detailed experimental procedure for M_x-binding characterisation 24

10 Detailed experimental procedure for 1-fluorescence channel monomers leak reaction characterisation 25

11 Detailed experimental procedure to measure dimerization kinetics 27

12 Detailed experimental procedure to test product inhibition 28

13 Detailed experimental procedure for dimerization kinetics with labeled N_y. . . 30

14 Detailed experimental procedure for sequence-specific copying by templated dimerization. 31

15 Fitted TMSD rate constant k_t. 46

16 Fitted leak reaction rate k_{leak}. 49

17 Fitted k_h. 51

18 Initial rates of monomer turnover by T_{13} in the presence of M_1N_3 (10 nM-scale). 54

19 Initial rates of monomer turnover by T_{13} in the presence of M_1N_3 (100 nM-scale). 56

20 Initial rates for monomer turnover by T_{xy} used during specific dimerization assays. 58
21 Templating precision of first specific-dimerization iteration. 74
22 Templating precision of second specific-dimerization iteration. 74
23 Templating precision of third specific-dimerization iteration. 74

Supplementary figures

1 Calibration: M_1 strand alone (Alexa Fluor® 488) at 25° C. Gain 1261 17
2 Calibration: M_1L complex. (Quenched Alexa Fluor® 488) at 25° C. Detector Gain 1261 17
3 Calibration: $T_{13}M_1$ complex (Alexa Fluor® 488) at 25° C. Detector Gain 1261 18
4 Calibration: M_1N_3 complex (Alexa Fluor® 488) at 25° C. Detector Gain 1261 18
5 Example of M_xL binding characterisation. 24
6 Example of dimerization experiments with N_3 (single fluorescent channel). . 27
7 Example of dimerization experiments with labeled N_y (multichannel fluorescence experiment). 30
8 Raw data showing the kinetics of the sequence-specific copying by templated dimerization. 32
9 Example of the result of fluorescence data processing for single fluorophore experiments. 38
10 Fits of k_t for different primary toehold lengths t. 47
11 Fits of k_t for different competing templates. 48
12 Fits of k_{leak} for different combination of monomers. 50
13 Fits of k_h for different handhold lengths. 51
14 Primary toehold length doesn't affect the HMSD kinetics. 52
15 Kinetics of 10 nM-scale inhibition tests. 55
16 Kinetics of 100 nM-scale inhibition tests. 57
17 Kinetics of the T_{xy}-mediated reaction in isolation. 59
18 Linear fits of sequenze-specific dimerization templates' initial rates to obtain their TOF. 60
19 Outline design of a system that exploits HMSD to copy a longer template . . 62
20 Performance of the clamp-free version of the dimerization system 65
21 Exploring reaction kinetics for the original version of the displacement domain 66

22 Kinetics of first iteration of sequence-specific dimerization experiment 68
23 PAGE of first iteration of sequence-specific dimerization experiment 69
24 Individual scans of the first iteration PAGE of sequence-specific dimerization experiment. 69
25 Kinetics of second iteration of sequence-specific dimerization experiment . . 70
26 PAGE of second iteration of sequence-specific dimerization experiment . . . 71
27 Individual scans of the second iteration PAGE of sequence-specific dimerization experiment. 71
28 Negative controls for specific dimerization experiments. 72
29 Individual scans of the third iteration PAGE of sequence-specific dimerization experiment. 72
30 Template-mediated interconversion of products 76
31 Secondary toehold mediated TMSD reaction without handhold complmentarity 78
32 Evidence of the effective irreversibility of the displacement of L from M_1L by T_{13} 79
33 Identification of bands in the gel in Figure 6b of the main text (separate monomer pools) 82
34 Identification of bands in the gel in Figure 6b of the main text (combined monomer pools) 83
35 Polyacrylamide gel electrophoresis of trimerisation controls 84

1 Supplementary Note 1: A Definition of Information Propagation by the Catalytic Templating of Molecular Assembly

At a formal level, a system that exhibits information propagation via templating is described in the language of chemical reaction networks (CRNs) as a system that possesses a set of "monomer" species $Q = \{Q_1, Q_2, ...\}$ that can undergo a series of reactions with the overall stoichiometry $\sum_{i=1}^{N_{S^j}} S_i^j + T_{S^j} \rightarrow S^j + T_{S^j}$. Here, S^j is a product specified by a "sequence" $\vec{S^j} = (S_1^j, S_2^j, ...S_{N_{S^j}}^j)$ of monomers each drawn from the set Q, and T_{S^j} is a template that catalyzes the overall process. Additional fuel or catalyst molecules – implicit here – may also contribute to the reaction.

To propagate information, it is necessary that multiple distinct templates $\{T_{S^1}, T_{S^2}...\}$ can catalyze the formation of multiple distinct products $\{S^1, S^2...\}$ with a high degree of specificity (each template predominantly catalyzes the formation of one product) and orthogonality (different templates catalyze different products). Further, each monomer Q_i should appear in multiple possible sequences when catalyzed by the appropriate templates, allowing the number of accessible products to grow non-linearly with the number of monomer species.

In principle, one can formally approximate any finite CRN using synthetic DNA and TMSD reactions involving multi-stranded gates,[1,2] including schemes that would obey the above definition. In these schemes, molecules of the species representing T_{S^j} and $\vec{S^j}$ would interact with one or more multistranded "gate" complexes, which would then release T_{S^j} and a molecule representing species S^j. At a physical level, however, these gate-mediated reactions would be pseudo-catalytic,[3] in that they would actually consume one catalyst molecule and then release a distinct one, and the catalyst would not directly interact with the monomers or product. Moreover, the product would not be formed by directly concatenating (moieties from) the monomer molecules consumed.

Biologically, templating is used to assemble complex macromolecules from simple monomers. To be useful in this regard, the product must be assembled by the templating reaction, rather than simply released from a pre-assembled gate complex by the appropriate triggers. It is thus necessary to augment the CRN-level description of templating above with a physical requirement that the product consists of a concatenation of (moieties from) the input monomer molecules.

2 Supplementary Note 2: DNA sequences

Strands used for each figure are listed in Supplementary Table 6.

2.1 Sequences for characterisation of the basic motif

All sequences used are collected in Supplementary Tables 1 to 3. Strands are named after the sequence and length of their primary toehold (t^1) and handhold domain (h). Template strands (T_{xy} (ut/vh) can bind by the t^1 domain to the pool of monomer M duplexes (M_xL), and by the h domain the pool of complementary monomer N strands (N_y).

T_{xy}, M_x and N_y strands have, by default, toehold domains of length 6nt and handhold domains of length 8nt. To test variants of t and h, we used the following:

- To test toeholds longer than 6, we used M_1 with toeholds of length 8 and T_{13} with with toeholds of length 7 and 8.
- To test handholds longer than 8, we used N_3 with handholds of length 10 and T_{13} with with handholds of length 9 and 10.
- To test shorter handholds or toeholds, we used M_x and N_y strands with toehold domains of length 6nt and handhold domains of length 8nt, and truncated versions of T_{xy}.

Sequences are colored by domain using the following colors:

Red: Mismatches whose repair drives the dimerisation reaction.

Grey: L_x dimerization domain that binds to M_x. Complementary to blue. The same sequence as orange and green, but contains two mismatches with M_x.

Orange: T_{xy} dimerization domain that binds to M_x, and is displaced during HMSD by N_y's green dimerization domain. Same sequence as gray and green, but contains one mismatch with M_x.

Green: N_y dimerization domain that binds to M_x in order to create the dimer. Same sequence as gray and orange, but it does not contain any mismatch with M_x.

Blue: M_x strands' dimerization domain. Complementary to gray, orange, and green.

Gold: Handhold domain of T_{xy} strands and handhold-complementary domains in N_y strands. Variable length and sequence in T_{xy} and N_y strands .

Purple: Toehold recognition domain of T_{xy} strands and recognition-complementary toehold domains in M_x strands. These domains constitute the primary toehold. Variable length and sequence in T_{xy} and M_x strands.

Light blue: Secondary toehold (t^2) domain and its complement. Always the same 2 nt sequence. Present in M_x; the complement is present in L_x and N_y strands

Black: 2 nt spacer domains between orange and gold domains in T_{xy} strands; poly-T spacers; and clamp nucleotides in M_x, L_x and T_{xy} strands.

2.2 Strands for trimerization via the action of two catalysts

Strands used in the trimerization experiments are listed in Supplementary Table 4. We attempt to generalise the colour convention from the basic characterization. Note that the recognition domain of monomer B acts as both a toehold and a handhold, depending on the reaction; we have coloured it as a toehold.

The strand design is not directly adapted from the dimerization systems of Supplementary Tables 1 to 3, but from strands used to investigate trimerization via a single template. Although the underlying design principles are similar, therefore, the strand designs are slightly different from the designs optimized for dimerization in Supplementary Tables 1 to 3.

2.3 Strands for templating of covalent bond formation

These sequences were adapted directly from the templating of M_1N_1 via T_{11}. We simply replaced fluorophores and quenchers with the necessary moieties for the CuAAC reaction.

Additional strands are listed in Supplementary Table 5. We use identical color coding of domains to strands for the basic characterization.

Supplementary Table 1: Template strands (T_{xy}).

	Sequence	Size (nt)
T_{11}	5’ - GTT TAG GCG TTT TAT CTT CAC TTC CAT CCA TTC CAG TTC CAT TAG CGT TGA TGA GGA - 3’	57
T_{12}	5’ - GTT TAG GCG TTT TAT CTT CAC TTC CAT CCA TTC CAG TTC CAT TAG CGT TGA GTA CTG - 3’	57
T_{13} (6t/8h)	5’ - GTT TAG GCG TTT TAT CTT CAC TTC CAT CCA TTC CAG TTC CAT TAG CGT TGT GGT AAG - 3’	57
T_{21}	5’ - TTG TCA GCG TTT TAT CTT CAC TTC CAT CCA TTC CAG TTC CAT TAG CGT TGA TGA GGA - 3’	57
T_{22}	5’ - TTG TCA GCG TTT TAT CTT CAC TTC CAT CCA TTC CAG TTC CAT TAG CGT TGA GTA CTG - 3’	57
T_{23}	5’ - TTG TCA GCG TTT TAT CTT CAC TTC CAT CCA TTC CAG TTC CAT TAG CGT TGT GGT AAG - 3’	57
T_{31}	5’ - ATT CTT GCG TTT TAT CTT CAC TTC CAT CCA TTC CAG TTC CAT TAG CGT TGA TGA GGA - 3’	57
T_{32}	5’ - ATT CTT GCG TTT TAT CTT CAC TTC CAT CCA TTC CAG TTC CAT TAG CGT TGA GTA CTG - 3’	57
T_{33}	5’ - ATT CTT GCG TTT TAT CTT CAC TTC CAT CCA TTC CAG TTC CAT TAG CGT TGT GGT AAG - 3’	57
T_{13} (4t/6h)	5’ - TTA GGC GTT TTA TCT TCA CTT CCA TCC ATT CCA GTT CCA TTA GCG TTG TGG TA - 3’	53
T_{13} (4t/7h)	5’ - TTA GGC GTT TTA TCT TCA CTT CCA TCC ATT CCA GTT CCA TTA GCG TTG TGG TAA - 3’	54
T_{13} (4t/8h)	5’ - TTA GGC GTT TTA TCT TCA CTT CCA TCC ATT CCA GTT CCA TTA GCG TTG TGG TAA G - 3’	55
T_{13} (4t/9h)	5’ - TTA GGC GTT TTA TCT TCA CTT CCA TCC ATT CCA GTT CCA TTA GCG TTG TGG TAA GA - 3’	56
T_{13} (4t/10h)	5’ - TTA GGC GTT TTA TCT TCA CTT CCA TCC ATT CCA GTT CCA TTA GCG TTG TGG TAA GAG - 3’	57
T_{13} (5t/6h)	5’ - TTT AGG CGT TTT ATC TTC ACT TCC ATC CAT TCC AGT TCC ATT AGC GTT GTG GTA - 3’	54
T_{13} (5t/7h)	5’ - TTT AGG CGT TTT ATC TTC ACT TCC ATC CAT TCC AGT TCC ATT AGC GTT GTG GTA A - 3’	55
T_{13} (5t/8h)	5’ - TTT AGG CGT TTT ATC TTC ACT TCC ATC CAT TCC AGT TCC ATT AGC GTT GTG GTA AG - 3’	56
T_{13} (5t/9h)	5’ - TTT AGG CGT TTT ATC TTC ACT TCC ATC CAT TCC AGT TCC ATT AGC GTT GTG GTA AGA - 3’	57
T_{13} (5t/10h)	5’ - TTT AGG CGT TTT ATC TTC ACT TCC ATC CAT TCC AGT TCC ATT AGC GTT GTG GTA AGA G - 3’	58
T_{13} (6t/6h)	5’ - GTT TAG GCG TTT TAT CTT CAC TTC CAT CCA TTC CAG TTC CAT TAG CGT TGT GGT A - 3’	55
T_{13} (6t/7h)	5’ - GTT TAG GCG TTT TAT CTT CAC TTC CAT CCA TTC CAG TTC CAT TAG CGT TGT GGT AA - 3’	56
T_{13} (6t/9h)	5’ - GTT TAG GCG TTT TAT CTT CAC TTC CAT CCA TTC CAG TTC CAT TAG CGT TGT GGT AAG A - 3’	57
T_{13} (6t/10h)	5’ - GTT TAG GCG TTT TAT CTT CAC TTC CAT CCA TTC CAG TTC CAT TAG CGT TGT GGT AAG AG - 3’	58
T_{13} (7t/6h)	5’ - TGT TTA GGC GTT TTA TCT TCA CTT CCA TCC ATT CCA GTT CCA TTA GCG TTG TGG TA - 3’	56
T_{13} (7t/7h)	5’ - TGT TTA GGC GTT TTA TCT TCA CTT CCA TCC ATT CCA GTT CCA TTA GCG TTG TGG TAA - 3’	57
T_{13} (7t/8h)	5’ - TGT TTA GGC GTT TTA TCT TCA CTT CCA TCC ATT CCA GTT CCA TTA GCG TTG TGG TAA G - 3’	58
T_{13} (7t/9h)	5’ - TGT TTA GGC GTT TTA TCT TCA CTT CCA TCC ATT CCA GTT CCA TTA GCG TTG TGG TAA GA - 3’	59
T_{13} (7t/10h)	5’ - TGT TTA GGC GTT TTA TCT TCA CTT CCA TCC ATT CCA GTT CCA TTA GCG TTG TGG TAA GAG- 3’	60
T_{13} (8t/6h)	5’ - TTG TTT AGG CGT TTT ATC TTC ACT TCC ATC CAT TCC AGT TCC ATT AGC GTT GTG GTA - 3’	57
T_{13} (8t/7h)	5’ - TTG TTT AGG CGT TTT ATC TTC ACT TCC ATC CAT TCC AGT TCC ATT AGC GTT GTG GTA A - 3’	58
T_{13} (8t/8h)	5’ - TTG TTT AGG CGT TTT ATC TTC ACT TCC ATC CAT TCC AGT TCC ATT AGC GTT GTG GTA AG - 3’	59
T_{13} (8t/9h)	5’ - TTG TTT AGG CGT TTT ATC TTC ACT TCC ATC CAT TCC AGT TCC ATT AGC GTT GTG GTA AGA - 3’	60
T_{13} (8t/10h)	5’ - TTG TTT AGG CGT TTT ATC TTC ACT TCC ATC CAT TCC AGT TCC ATT AGC GTT GTG GTA AGA G - 3’	61
T_{13} v.1	5’ - GGA AAC A CG CAT CAT A C T CAA GTC AAA GTC AAG TCA TAT TCA GGG - 3’	45

Supplementary Table 2: Monomer M (M_x) and its lock strands (L_x).

	Sequence	Size (nt)
M_1	5' - **AlexaFluor®488** – ttt **C**GT CGC TAA TGG AAC TGG AAT GGA TGG AAC TGA AGA TAA AAC G**C**C TAA AC - 3'	53
M_2	5' - **AlexaFluor®546** – ttt ttt ttt ttt **C**GT CGC TAA TGG AAC TGG AAT GGA TGG AAC TGA AGA TAA AAC G**C**T GAC AA - 3'	62
M_3	5' - **AlexaFluor®647** – ttt ttt **C**GT CGC TAA TGG AAC TGG AAT GGA TGG AAC TGA AGA TAA AAC G**C**A AGA AT - 3'	56
M_1 (Unlab.)	5' - ttt **C**GT CGC TAA TGG AAC TGG AAT GGA TGG AAC TGA AGA TAA AAC G**C**C TAA AC - 3'	53
M_1 (8t)	5' - **AlexaFluor®488** – ttt **C**GT CGC TAA TGG AAC TGG AAT GGA TGG AAC TGA AGA TAA AAC G **C**C TAA ACA A - 3'	55
M_1 (8t Unlab.)	5' - ttt **C**GT CGC TAA TGG AAC TGG AAT GGA TGG AAC TGA AGA TAA AAC G**C**C TAA ACA A - 3'	55
M_1 v.1	5' - **AlexaFluor®488** – ttt CCG ACT TGA CTT TGA CTT GAC TAT GAT GCGTGT TTC - 3'	38
L_1	5' - **G**CG TTT TAT CTT CA**C** TTC CAT CCA TTC CA**C** TTC CAT TAG CGA C**G** ttt – **IowaBlack®FQ** - 3'	47
L_2	5' - **G**CG TTT TAT CTT CA**C** TTC CAT CCA TTC CA**C** TTC CAT TAG CGA C**G** ttt ttt ttt ttt – **BlackHoleQuencher®2** - 3'	53
L_3	5' - **G**CG TTT TAT CTT CA**C** TTC CAT CCA TTC CAC TTC CAT TAG CGA C**G** ttt ttt – **IowaBlack®RQ** - 3'	50
L_1 v.1	5' - CGC ATC ATA**C** TC AAG TCA AA**C** TCA AGT CGG ttt – **BlackHoleQuencher®1** - 3'	33

Supplementary Table 3: Monomer N (N_y) strands.

	Sequence	Size (nt)
N_1	5' - TCC TCA TCC GTT TTA TCT TCA GTT CCA TCC ATT CCA GTT CCA TTA GCG AC ttt ttt ttt – **AlexaFluor®647** - 3'	59
N_2	5' - CAG TAC TCC GTT TTA TCT TCA GTT CCA TCC ATT CCA GTT CCA TTA GCG AC ttt – **AlexaFluor®546** - 3'	53
N_3	5' - CTT ACC ACC GTT TTA TCT TCA GTT CCA TCC ATT CCA GTT CCA TTA GCG AC - 3'	50
N_3 (10h)	5' - CTC TTA CCA CCG TTT TAT CTT CAG TTC CAT CCA TTC CAG TTC CAT TAG CGA C - 3'	52
N_3 v.1	5' - CCC TGA ATC GCA TCA TAG TCA AGT CAA AGT CAA GTCGG - 3'	38
N v.1 (0h)	5' - C GCA TCA TAG TCA AGT CAA AGT CAA GTCGG - 3'	30

Supplementary Table 4: Sequences for trimerization experiments.

	Sequence	Size (nt)
A_1	5' - GTA AGA GCG CAA CTC CTC ATC ACC ACC TTC CCA ATT ACG CCG ttt- AlexaFluor®647 -3'	45
B_1	5' - GGC GTA ATT GGG AAG GTG GTG ATG AGG AGT TGC GGA AAG ACG CAA CCA CCA AAC AAC AAT AAC ACA TCA TCG CGG ttt - AlexaFluor®546 - 3'	78
C_1	5' - AlexaFluor®488 - ttt CGC GAT GAT GTG TTA TTG TTG TTT GGT GGT TGC ATG ATG GGA C - 3'	46
A_2	5' - ttt tt ACC ATT TCG CAA CTC CTC ATC ACC ACC TTC CCA ATT ACG CCG ttt- AlexaFluor®488 -3'	45
B_2	5' - tt GGC GTA ATT GGG AAG GTG GTG ATG AGG AGT TGC CGG CGT TCG CAA CCA CCA AAC AAC AAT AAC ACA TCA TCG CGG ttt - AlexaFluor®647 - 3'	78
C_2	5' - AlexaFluor®546 - ttt CGC GAT GAT GTG TTA TTG TTG TTT GGT GGT TGC GTG TCC TAT T ttt tt - 3'	46
$T_{AB_{11}}$	5' - TCT TTC CCG TAA TTC GGA AGG TCG TGA TGA GGA GTT GCG CTC TTA C - 3'	46
$T_{BC_{11}}$	5' - CCA TCA TCG ATG ATC TGT TAT TCT TGT TTG GTG GTT GC GTCT TTC C - 3'	46
$T_{AB_{22}}$	5' - AAC GCC GCG TAA TTC GGA AGG TCG TGA TGA GGA GTT GC G AAA TGG T - 3'	46
$T_{BC_{22}}$	5' - AGG ACA CCG ATG ATC TGT TAT TCT TGT TTG GTG GTT GC G AAC GCC G - 3'	46
L_A	5' - IowaBlack®RQ - ttt CGG CGT AAT TCG GAA GGT CGT GAT GAC GAG TTG CG - 3'	38
L_B	5' - IowaBlack®RQ - ttt CCG CGA TGA TCT GTT ATT CTT GTT TGC TGG TTG CG - 3'	38

Supplementary Table 5: Additional strands for covalent bond formation.

	Sequence	Size (n
$M_{1-\mathrm{alk}}$	5' - Hexynyl - CGT CGC TAA TGG AAC TGG AAT GGA TGG AAC TGA AGA TAA AAC GCC TAA AC - 3'	50
$N_{1-\mathrm{aza}}$	5' - TCC TCA TCC GTT TTA TCT TCA GTT CCA TCC ATT CCA GTT CCA TTA GCG AC - Azide (NHS ester, C6 linker, C6 amino modifier) - 3'	50
$L_{1-\mathrm{alk}}$	5' - GCG TTT TAT CTT CAC TTC CAT CCA TTC CAC TTC CAT TAG CGA CG - 3'	44

Supplementary Table 6: Strands used in each figure of this publication.

Figure	Strands used
**2**	T_{13} (4-8t/6-10h), M_1, M_1 (8t), L_1, N_3, N_3 (10h)
**3a**	T_{13} (5-7t/8-10h), M_1, M_1 (Unlab.), M_1 (8t), M_1 (8t Unlab.), L_1, N_3, N_3 (10h)
**3c**	T_{13} (6t/8h), M_1, L_1, N_3
**4b**	T_{11}, T_{12}, T_{13} (6t/8h), T_{21}, T_{22}, T_{23}, T_{31}, T_{32}, T_{33}, M_1, M_2, M_3, L_1, L_2, L_3, N_1, N_2, N_3
**5**	T_{11}, T_{12}, T_{13} (6t/8h), T_{21}, T_{22}, T_{23}, T_{31}, T_{32}, T_{33}, M_1, M_2, M_3, L_1, L_2, L_3, N_1, N_2, N_3
**6b**	A_1, B_1, C_1, A_2, B_2, C_2, $T_{AB_{11}}$, $T_{BC_{11}}$, $T_{AB_{22}}$, $T_{BC_{22}}$, L_A, L_B
**6d**	$M_{1-\mathrm{alk}}$, $N_{1-\mathrm{aza}}$, $L_{1-\mathrm{alk}}$, T_{11}
**Sup.1**	M_1
**Sup.2**	M_1, L_1
**Sup.3**	M_1, L_1, T_{13}(6t/8h)
**Sup.4**	M_1, L_1, T_{13}(6t/8h), N_3
**Sup.5**	M_1, L_1, T_{12}
**Sup.6**	M_2, L_2, T_{23}, N_3
**Sup.7**	M_1, L_1, T_{12}, N_2
**Sup.8**	T_{11}, T_{12}, T_{13} (6t/8h), T_{21}, T_{22}, T_{23}, T_{31}, T_{32}, T_{33}, M_1, M_2, M_3, L_1, L_2, L_3, N_1, N_2, N_3
**Sup.9**	M_3, L_3, T_{33}, N_3
**Sup.10**	M_1, L_1, T_{13}(4t/8h), T_{13}(5t/8h), T_{13}(6t/8h), T_{13}(7t/8h), T_{13}(8t/8h)
**Sup.11**	M_1, M_2, M_3, L_1, L_2, L_3, T_{11}, T_{12}, T_{13}(6t/8h), T_{21}, T_{22}, T_{23}, T_{31}, T_{32}, T_{33}
**Sup.12**	M_1, M_2, M_3, L_1, L_2, L_3, N_1, N_2, N_3
**Sup.13**	M_1, L_1, N_3, T_{13}(6t/6h), T_{13}(6t/7h), T_{13}(6t/9h), T_{13}(6t/10h)
**Sup.14**	M_1, L_1, N_3, T_{13}(4t/8h), T_{13}(5t/8h), T_{13}(6t/8h), T_{13}(7t/8h), T_{13}(8t/8h)
**Sup.15**	T_{13} (4-8t/6-10h), M_1, M_1 (Unlab.), M_1 (8t), M_1 (8t Unlab.), L_1, N_3, N_3 (10h)
**Sup.16**	T_{13} (6t/8h), M_1, M_1 (Unlab.), L_1, N_3
**Sup.17**	T_{11}, T_{12}, T_{13} (6t/8h), T_{21}, T_{22}, T_{23}, T_{31}, T_{32}, T_{33}, M_1, M_2, M_3, L_1, L_2, L_3, N_1, N_2, N_3
**Sup.20**	T_{13} v.1, M_1 v.1, L_1 v.1, N_3 v.1
**Sup.21a**	T_{13} v.1, M_1 v.1, L_1 v.1
**Sup.21b**	T_{13}, T_{13} v.1, M_1, M_1 v.1, L_1, L_1 v.1, N_3, N_3 v.1, N_3 v.1 (0h)
**Sup.22-27**	T_{11}, T_{12}, T_{13} (6t/8h), T_{21}, T_{22}, T_{23}, T_{31}, T_{32}, T_{33}, M_1, M_2, M_3, L_1, L_2, L_3, N_1, N_2, N_3
**Sup.28**	M_1, M_2, M_3, L_1, L_2, L_3, N_1, N_2, N_3
**Sup.29**	T_{11}, T_{12}, T_{13} (6t/8h), T_{21}, T_{22}, T_{23}, T_{31}, T_{32}, T_{33}, M_1, M_2, M_3, L_1, L_2, L_3, N_1, N_2, N_3
**Sup.30a**	T_{11}, M_1, L_1, N_1,
**Sup.30b**	T_{12}, T_{33}, M_1, N_1, N_3
**Sup.31a**	T_{12},T_{22}, T_{32}, M_1, M_2, M_3, L_1, L_2, L_3, N_3
**Sup.31b**	T_{12}, M_1, L_1, N_1
**Sup.32**	T_{13} (6t/8h), M_1, L_1, N_3
**Sup.33**	A_1, B_1, C_1, A_2, B_2, C_2, $T_{AB_{11}}$, $T_{BC_{11}}$, $T_{AB_{22}}$, $T_{BC_{22}}$, L_A, L_B

3 Supplementary Note 3: Fluorescent species calibration

This section describes the methods used to obtain calibration curves for converting the raw fluorescence data from the experiments into concentration estimates. It also presents the results of calibration.

In our experiments, we measure fluorescence in a plate reader from the bottom of the plate. Reactions are triggered injecting liquid into the plate, meaning that the volume changes (by up to 50% over the full course of the measurement). Typically, the change in fluorescence measured in response to such a dilution is small, because the path length of fluid in the illumination/detection region increases to compensate for a decrease in concentration.[4] Moreover, adjustment of the focusing height of the plate reader can provide fine adjustment to the relative fluorescence before and after dilution. We selected a focusing height such that the dilution didn't produce a significant fluorescence difference between the volumes of 150 µL and 200 µL most commonly used in our experiments. As a result, the fluorescence signals effectively report the molecular count of certain species in the well. For convenience, however, we will refer to these molecular counts through a multi-step experiment in terms of their concentration as if they were solvated in a 200 µL volume (the volume at which reaction kinetics were measured).

For our experimental setup, the signal measured by the plate reader is assumed to be a linear function of this effective molecular concentration of fluorescent species. The constant term is assumed to be a background offset equivalent to the fluorescence of a buffer-only control. Therefore, after subtracting the median fluorescence of the negative control from all values, a weighted linear regression model was used to fit a proportional expression for the median fluorescence F of each well as a function of molecular concentration x, $F(x) = ax$. Here, a is the fluorescent count per nM of the labelled species for the measurement conditions; we describe this quantity as a "coefficient of fluorescence". The only exception to the above

was the weakly-fluorescent species M_1L, which required an offset to account for a background signal.

Fitting was performed with the MATLAB R2019a Optimization Toolbox.[5] The residuals were weighted with the inverse of the fluorescence reading to account for the Poisson distribution of the photomultiplier counts .[6] The regression coefficient value is reported with 95% confidence bounds. Fitting results and their residuals for the fluorescence of M_1 in various complexes are shown in Supplementary Figures 1 to 4.

3.1 Single channel calibration of Alexa Fluor® 488 for complexes involved in M_1N_3 formation

To a first approximation, the M_1L complex has very low fluorescence, and all other complexes containing M_1 have high fluorescence. However, the fluorescence of M_1L is not zero, and other complexes of M_1 show variation in the fluorescence signal due to changes in the sequence environment of the fluorophores. Calibration curves were used to estimate the signal variation between the different hybridization states of M_1, and the fluorescence of M_1L.

3.1.1 Protocol

1. Three M_1 sets of serial dilutions with concentrations ranging from 0 to 150 nM were prepared in a volume of 150 µL. Fluorescence was measured and fitted using linear regression (Figure 1), giving the coefficient of fluorescence of M_1.

2. One of the M_1 sets of serial dilutions was brought to a volume of 195 µL, with an injection of L_1 sufficient to achieve a $[L_1] = 500$ nM. The fluorescence data (Figure 2) allows for the calculation of the coefficient of fluorescence of M_1L.

3. That same serial dilution was brought to a volume of 200 µL with an injection of T_{13} sufficient to give $[T_{13}] = 200$ nM. Another set of serial dilutions containing just M_1 was brought to the same volume using T_{13} ($[T_{13}] = 200$ nM) for comparison and to

demonstrate that the displacement of L_1 from M_1L by the added T_{13} was complete. The fluorescence data of both replicas (Figure 3) allows for the calculation of the coefficient of fluorescence of M_1T_{13}.

4. All three sets of serial dilutions were then brought to 205 µL with an injection of N_3, sufficient to give a concentration of $[N_3] = 500$ nM. The three curves (Figure 4) were used to calculate the coefficient of fluorescence of M_1N_3. Their consistency is indicative of the fact that a large excess of N_3 is sufficient to convert all M_1 into M_1N_3 complexes, regardless of the presence of excess concentrations of L or T_{13}.

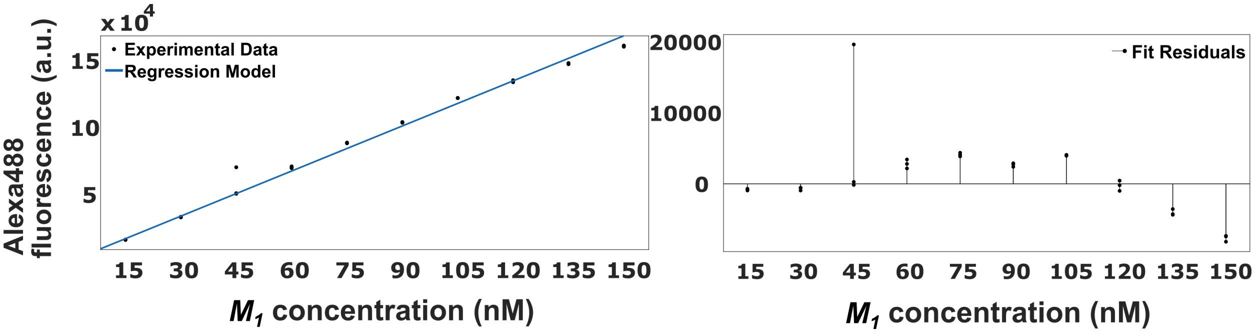

Supplementary Figure 1: Calibration: M_1 strand (Alexa Fluor® 488) at 25° C. Detector Gain 1261. 3 replicas per point. Left: Regression line. Right: Fit residuals. a = 1123 fluo a.u. nM^{-1} (1106, 1140). Goodness of fit: SSE = 4425. R-square = 0.9959. RMSE = 12.36.

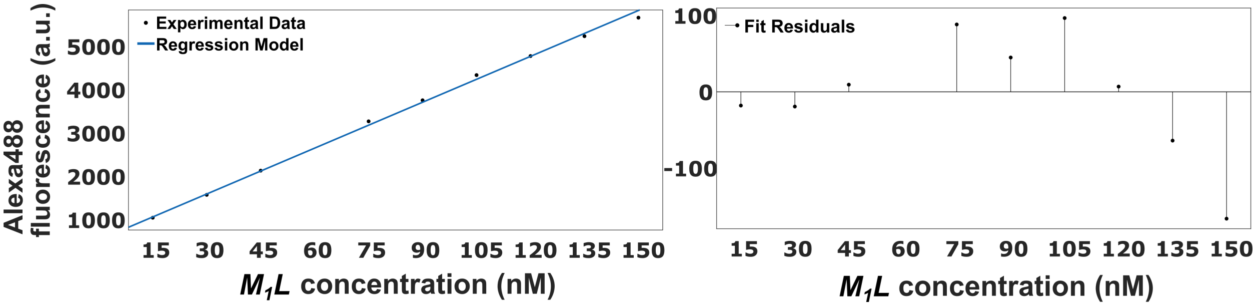

Supplementary Figure 2: Calibration: M_1L complex (Quenched Alexa Fluor® 488) at 25° C. Detector Gain 1261. Added L in excess. Only one replica per point. Left: Regression line. Right: Fit residuals. a = 35.24 fluo a.u. nM^{-1} (33.86, 36.63). The calibration line had a non-negligible background (b = 550.2 (449.6, 650.8)). This term is probably due to wrongly annealed duplexes, as it doesn't appear in other calibrations using thermocycler-annealed strands. Therefore, it was ignored in subsequent analysis. Goodness of fit: SSE = 19.28. R-square = 0.9977. Adjusted R-square = 0.9974. RMSE = 1.552.

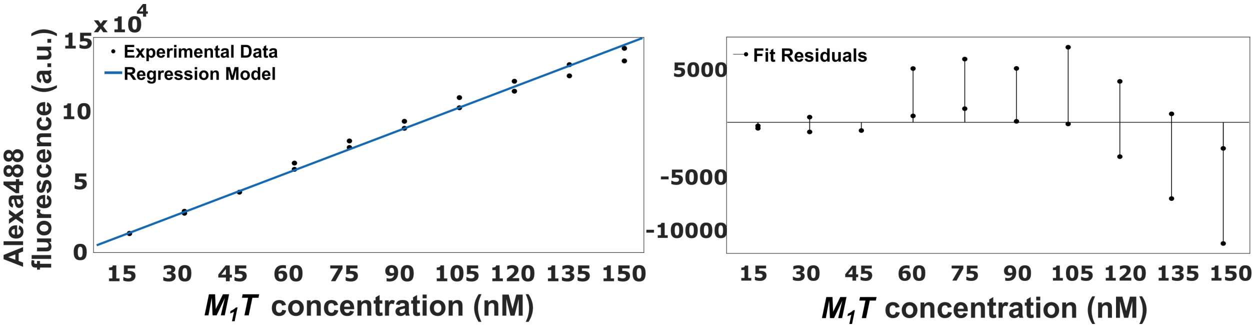

Supplementary Figure 3: Calibration: $T_{13}M_1$ complex (Alexa Fluor® 488) at 25° C. Detector Gain 1261. Triggered M_1L with excess of T_{13}. Only one replica per point. Left: Regression line. Right: Fit residuals. a = 979 fluo a.u. nM^{-1} (957.6, 1000). Goodness of fit: SSE = 3241. R-square = 0.995. RMSE = 13.06.

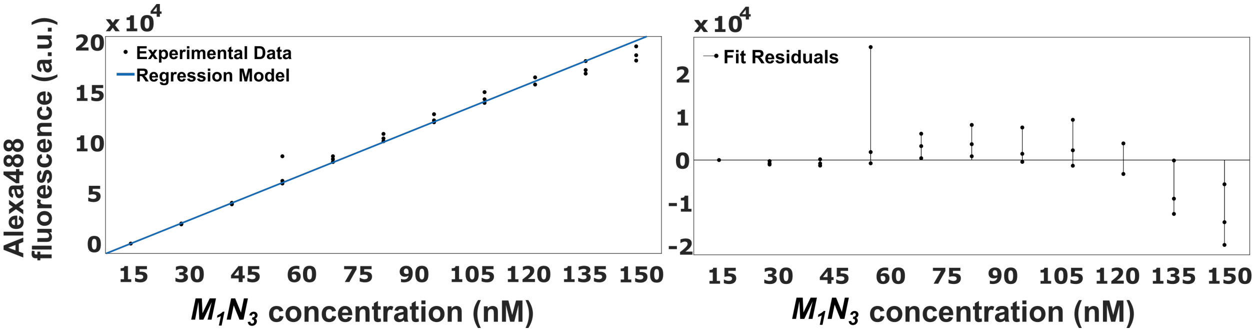

Supplementary Figure 4: Calibration: M_1N_3 complex (Alexa Fluor® 488) at 25° C. Detector Gain 1261. Three replicas; consistency shows that saturating N_3 is able to bind to all M_1. Left: Regression line. Right: Fit residuals. a = 1342 fluo a.u. nM^{-1} (1317, 1367). Goodness of fit: SSE = 7893. R-square = 0.9935. RMSE = 16.5.

Multi-channel fluorescence calibration

Sequence-specific copying experiments involved the formation of several M_xN_y dimers. Each M_xN_y had a different combination of fluorophores that produced a specific fluorescence emission that was measured in six different fluorescent channels. These species had a non-negligible bleed-through in the different fluorescence channels that had to be quantified for a correct deconvolution of the measured fluorescence signal. The data collected in Supplementary Tables 7 and 8 shows, for monomers and dimer products, the coefficient of fluorescence of each species in each channel. Each entry into this calibration matrix was obtained from a single set of three concentrations of the relevant species (25, 50, and 100 nM); these data

were fit assuming that fluorescence was proportional to the molecular concentration after accounting for background. We report two versions of the calibration matrix in Suppleementary Tables 7 and 8, since experimetns were performed using two different settings on the plate reader.

These experiments additionally confirmed two things. First, the quenching efficiency of each M_xL complex: $M_1L_1 = 97.1 \pm 0.3\,\%$; $M_2L_2 = 89.3 \pm 0.7\,\%$; $M_3L_3 = 89.4 \pm 1\%$. Second, the strands labeled with Alexa Fluor® 546 or 647 had essentially identical fluorescence in any complex that contained no other fluorophores (to within approximately 2%). In other words, M_3N_3 and N_1 could be described with a single set of parameters, as could M_2N_3 and N_2. The data presented in Supplementary Tables 7 and 8 for these complexes uses an average over these pairs.

Supplementary Table 7: Calibration matrix 1: multi-channel coefficient of fluorescence of the species present in sequence-specific copying experiments at 25° C. Calculated values of the coefficient of fluorescence of M_xL, M_xN_y and N_y (a if $f(x) = ax$, where f is fluorescence and x is concentration) for 6 different fluorescence channels. These values were used for all multichannel experiments, except iteration 1 of the sequence-specific dimerization experiments. Unlabelled N_3 showed no fluorescence above the background. **Bold:** Main channel used to quantify the species in each row. Blue = Alexa Fluor® 488 (EX: 488/14 nm; EM: 535/30 nm), Gain = 1261; Green = Alexa Fluor® 546 (EX: 540/20 nm; EM: 590/30 nm) Gain = 1304; Red = Alexa Fluor® 647 (EX: 625/30 nm; EM: 680/30 nm) Gain = 1896; B/G = FRET signal of Alexa Fluor® 488 and 546 (EX: 488/14 nm; EM: 590/30 nm), Gain = 1461; G/R = FRET signal of Alexa Fluor® 488 and 647 (EX: 488/14 nm; EM: 670/30 nm), Gain = 2259 and B/R = FRET signal of Alexa Fluor® 546 and 647 (EX: 540/20 nm; EM: 680/30 nm), Gain = 2257. Fitted data is collected in the data repository accompanying this publication.

Species	Blue	Green	Red	B/G	G/R	B/R
M_1N_1	410	0.71	625	88	198	**1363**
M_1N_2	200	679	0.17	**1229**	131	251
M_1N_3	**1332**	1.19	0.05	287	0.89	40
M_2N_1	4.1	260	684	63	**1289**	246
M_2N_2	10	**965**	0.04	235	193	54
M_2N_3 **or** N_2	11	**924**	0.003	224	174	49
M_3N_1	1.3	0.28	**1020**	0.54	310	48
M_3N_2	1.9	88	599	23	**1327**	250
M_3N_3 **or** N_1	0.72	0.29	**680**	0.35	200	29
M_1L_1	**39**	0.08	0.11	8.8	0.23	1.4
M_2L_2	1.5	**97**	0.03	24	19	5.3
M_3L_3	0.52	0.05	**73**	0.15	23	3.7

Supplementary Table 8: Calibration matrix 2: multi-channel coefficient of fluorescence (fluorescence emission per nM) of the species present in sequence-specific copying experiments at 25° C. Calculated values of the coefficient of fluorescence of M_xL, M_xN_y and N_y (a if $F(x) = ax$, where F is fluorescence and x is concentration) for 6 different fluorescence channels. This calibration is used for the 1st iteration of the sequence-specific dimerization experiment (Supplementary Figure 22. Unlabelled N_3 showed no fluorescence above the background. **Bold:** Main channel used to quantify the species in each row. Blue = Alexa Fluor® 488 (EX: 488/14 nm; EM: 535/30 nm), Gain = 1508; Green = Alexa Fluor® 546 (EX: 540/20 nm; EM: 590/30 nm) Gain = 1552; Red = Alexa Fluor® 647 (EX: 625/30 nm; EM: 680/30 nm) Gain = 2169; B/G = FRET signal of Alexa Fluor® 488 and 546 (EX: 488/14 nm; EM: 590/30 nm), Gain = 1733; G/R = FRET signal of Alexa Fluor® 488 and 647 (EX: 488/14 nm; EM: 680/30 nm), Gain = 2554 and B/R = FRET signal of Alexa Fluor® 546 and 647 (EX: 540/20 nm; EM: 680/30 nm), Gain = 2607. Fitted data is collected in the data repository accompanying this publication

Species	Blue	Green	Red	B/G	G/R	B/R
M_1N_1	372	0.96	820	77.7	205	**1300**
M_1N_2	128	929	2.97	**1315**	144	255
M_1N_3	**1338**	1.65	2.85	280	1.14	37.4
M_2N_1	4.75	360	847	73.4	**1281**	244
M_2N_2	13	**1353**	0.15	277	213	58.3
M_2N_3 **or** N_2	12.8	**1197**	0.09	243	182	50
M_3N_1	1.09	0.43	**1358**	0	323	47.9
M_3N_2	2.16	153	781	32.6	**1395**	264
M_3N_3 **or** N_1	0.61	0.43	**900**	0	211	29.8
M_1L_1	**39.9**	0.1	0.13	9.57	0.14	1.68
M_2L_2	1.65	**140**	0.16	22.4	19.6	5.8
M_3L_3	0.88	0.11	**97**	0.13	22.9	3.84

4 Supplementary Note 4: Experimental procedures

Variations in both the concentration of stock oligonucleotides and pipetting and injection procedures mean that the actual concentration of strands in solution will differ from that intended in the experimental design. In some cases, we attempted to infer the actual concentration of strands in the experiment, as detailed below. Where a distinction is necessary, we refer to the “intended” concentration of the experimental design, and the “inferred” concentration apparent in experiments.

The designed experiments consisted of the measurement of reaction kinetics, alongside a set of ancillary measurements to determine reactant concentrations.[7] All experiments contained at least one negative (experimental buffer only) and one positive control per fluorescence channel measured. these controls were subjected to the same volume changes as the measured kinetics. Positive controls contained a variable concentration of fluorophore-labeled strands in the experimental buffer. Controls allowed the correction of changes in fluorescence due to environmental conditions. Data correction using the controls’ signal is further described in Supplementary Note 5.

All ancillary measurements contained at least 10 data points and quantified the fluorescence baseline of a steady state in the experiment. These measurements were used to estimate the concentration of each species in the system from the fluorescence signal steady states and were obtained either before the start of the reaction or by sequentially triggering the reaction of all the species (Supplementary Figure 5). These ancillary measurements helped to diminish the uncertainty in concentrations that can arise from a range of factors, including pipetting errors. The number and nature of the ancillary measurements were experiment-contingent; however, all the different experiments followed a similar structure, outlined below. In each case, the median value obtained during an ancillary measurement was used as “the” value of the measurement.

<u>Measurement 1. Initial fluorescence:</u> All experiments started by mixing a quenched complex (usually M_xL) in experimental buffer, for a total volume of 140 µL. The measurement

was made after 30 minutes of incubation at the experiment's temperature when the fluorescence value of the positive controls became stable.

Measurement 2. Additional species: For catalytic dimerization experiments that required the addition of template, 10 µL of a solution containing T_{xy} was added to the well. The addition of a template made an equimolar amount of complex M_xL react, increasing the fluorescence baseline.

Measurement 3. Experimental kinetics: This step involves the addition of 50 µL of a solution containing a reaction trigger species, usually N_y, resulting in a final reaction volume of 200 µL. In most cases, rather than perform replica experiments at identical concentrations, we used variants in which a single input species had a different concentration. These "replicas" allow a qualitative test for consistency and provide insight into the nature of the underlying reaction kinetics. We use the notation $[X, Y]$ to denote a set of concentrations in the range X to Y (inclusive).

The system kinetics were measured after the injection until the reaction reached a steady state. Kinetics that took more than 2 hours to reach a steady state were measured continuously for at least 50 hours after completely sealing the plate to prevent evaporation. After these measurements, the plate was incubated for up to a month to try to reach a steady state, sometimes after the addition of a low concentration of a reaction catalytic species, such as T_{xy}, to accelerate the approach to a steady state. The incubation was done at the experimental temperature and in dark conditions to avoid fluorophore photobleaching.

Measurement 4. Estimation of reaction trigger concentration: After the reaction reached a steady state, the resultant fluorescence plateau was recorded to infer the total amount of reaction trigger added in reactions (under the assumption that the reaction is irreversible).

Measurement 5. Saturation and quantification of the remaining species: To finish the assay, experiments were saturated with a combination of N_y and T_{xy}. This way, all the M_x monomer will be converted into M_xN_y complex, facilitating its quantification. Note that the fluorescence of the different reaction intermediaries can have variations up to 30% when

compared to M_xN_y (Supplementary Note 3.1).

The specific procedures for each type of experiment are discussed and collected in tables in the following subsections. As explained in Supplementary Note 3, species concentration is reported at any time as the concentration if the same amount of solute was dissolved in a reaction volume of 200 µL. This volume was chosen for being the one at which kinetics were always measured.

4.1 Single fluorescence channel experiments

4.1.1 Simple 2nd order reactions (M_x binding and monomer leak reactions)

The protocols, given in Supplementary Tables 9 and 10, do not include 'Measurement 2' as no initial T_{xy} is present in these experiments.

Binding rate of M_xL to T_{xy} (displacement of L from M_xL by T_{xy}). We measured the kinetics of displacement of L from M_xL by T_{xy} for every T_{xy} strand used during this work and fitted a TMSD rate constant k_t to the results. Experiments consisted of a solution of M_xL ($[M_xL]_0 = 100$ nM) triggered by a variable concentration of a complementary T_{xy} ($[T_{xy}]_0$ between 20 and 112.5 nM). The aim of testing some experimental replicas with $[T_{xy}]_0 > [M_x]_0$ was to confirm that T_{xy} reacts with a 1:1 stoichiometry with M_xL and discard a potential equilibration with its reversible reaction. Protocol steps are contained in Supplementary Table 9 and illustrated in Supplementary Figure 5. Additionally, Supplementary Figure 5 demonstrates that the fluorescence signal is proportional to the amount of T_{xy} injected.

M_xL and N_3 leak reactions. The kinetics of template-free leak reaction was obtained for the reaction between M_x and N_3 using single-channel fluorescence experiments. These data were used to fit a leak reaction rate constant, k_{leak}. Experiments consisted of a solution of M_xL ($[M_xL]_0 = 100$ nM) triggered by N_3 ($[N_3]_0 = [40, 90]$ nM). During 'Measurement 4', 5 nM of T_{x3} was injected for the reaction to reach completion. The estimated signal produced from the extra T_{x3} was later removed to estimate $[N_3]_0$. Protocol steps are contained in Supplementary Table 10.

Supplementary Table 9: Detailed experimental procedure for M_x-binding characterisation. Several trajectories for each T_{xy} condition and their controls were triggered by mechanical injection and measured simultaneously. The triggering species was a solution of T_{xy} with a complementary primary toehold. Since no T_{xy} was initially present in the experiment, no 'Measurement 2' was needed.

Steps	Addition	Purpose	Expected concentrations in 200 µL (nm)	Total volume (µL)
**Measurement 1**	10 µL M_xL (2 µM) + 140 µL Buffer	Estimate residual fluorescence baseline	$M_xL = 100$	150
**Measurement 3**	[50,10] µL T_{xy} (450 nM) + [0,40] µL Buffer	Experimental kinetics	Evolve with time	200
**Measurement 4**	-	Estimate $[T_{xy}]_0$	$M_xL =$ [77.5,0]; $M_1T_{xy} =$ [22.5,100]; Lock:[22.5,100]	200
**Measurement 5**	5 µL each of N_3 and T_{x3} (10 µM)	Estimate $[M_xL]_0$	$MN_{x3} = 100$; $L = 100$; T_{xy} and $N_3 \approx 200$	210

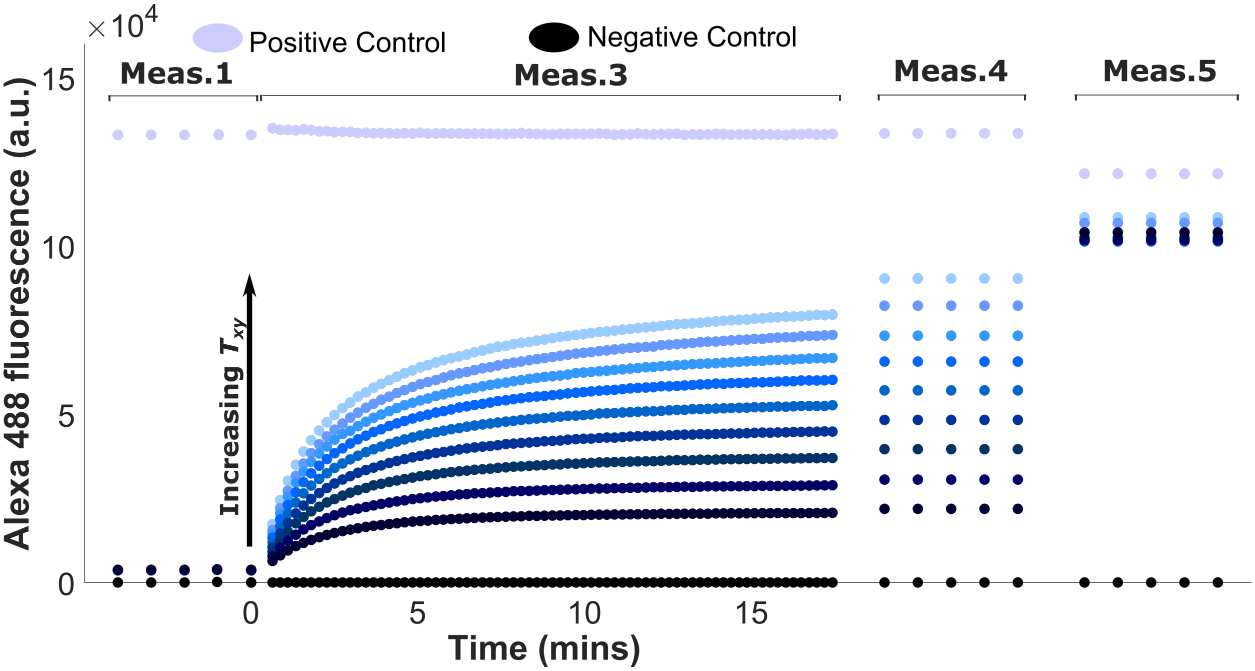

Supplementary Figure 5: Example of M_xL binding characterisation. Raw data from the displacement of L from the M_1L complex by adding strand T_{12} at 25° C. 'Measurement 1' contains the baseline of the experiments, considered as 0 nM of reacted M_1L. During 'Measurement 3', increasing amounts of T_{12} demonstrate that the signal produced is proportional to the concentration of T_{12}. After the reaction reached a steady state, $[T_{12}]$ was estimated from 'Measurement 4'. Afterwards, the solution was saturated with N_3 and T_{13} to transform all M_1L into M_1N_3 (the large excess of N_3 was sufficient to displace T_{12} from M_1T_{12} by the secondary toehold within a day). Fluorescence signal increased even for saturated conditions due to the transformation of M_1T_{12} into the complex M_1N_3. Concentrations: $[M_1L]_0 = 100$ nM, $[T_{12}]_0 =$ [101.25, 22.5] nM in increments of 11.25 nM. The positive control contained around 100 nM of M_1N_3. The negative controls consisted of only the experimental buffer.

Supplementary Table 10: Detailed experimental procedure for leak reaction characterisation for single-fluorescence channel monomers. Several trajectories were triggered by mechanical injection and measured simultaneously. The triggering species was a solution of N_3. Since no T_{xy} was initially present in the experiment, no 'Measurement 2' was needed.

Steps	Addition	Purpose	Expected concentrations in 200 µL (nm)	Total volume (µL)
**Measurement 1**	10 µL M_xL (2 µM) + 140 µL Buffer	Estimate residual fluorescence baseline	$M_xL = 100$	150
**Measurement 3**	[45,10] µL N_3 (400 nM) + [5,40] µL Buffer	Experimental kinetics	Evolve with time	200
**Measurement 4**	5 µL T_{x3} (200 nM)	Estimate $[N_3 + T_{x3}]$	M_xL = [75, 5]; M_xN_3 = [20, 90]; L = [25, 95]; $T_{x3}M_x = 5$	205
**Measurement 5**	5 µL each of N_3 and T_{x3} (10 µM)	Estimate $[M_xL]_0$	$M_xN_3 = 100$; $L = 100$; T_{x3} & $N_3 \approx 200$	215

4.1.2 Handhold-mediated reaction of M_1T_{13} with N_3

For a subset of the T_{13} variants, we studied the reaction between M_1T_{13} and N_3. The kinetics of this reaction could be measured by harnessing the significant increase in fluorescence resulting from the formation of M_1N_3 from M_1T_{13}. Experiments consisted of a solution of preannealed M_1T_{13}b(ut/vh) ($[M_1T_{13}]_0$ = 100 nM) triggered by N_3 ($[N_3]_0$ = [40, 90] nM). Protocol steps for handhold experiments are identical to the ones followed in Supplementary Table 10 except for no addition of T_{xy} in 'Measurement 4', no 'Measurement 5', and exchanging M_xL for M_1T_{13}.

4.1.3 Dimerization experiments using M_x, T_{x3} and N_3

These experiments were performed to explore system kinetics and product inhibition as a function of the length of primary toehold and handhold domains. We considered two regimes of monomer concentration for these experiments:

1. High monomer concentration experiments consisted of a solution of M_xL ($[M_xL]_0 = 100$ nM) triggered by N_3 ($[N_3]_0$ = 100 nM), 24 hours after adding a variable amount of T_{x3} ($[T_{x3}]_0$ = [10 - 0.25] nM) into the M_xL solution. These conditions were used to obtain the total turnover assays for all nine M_xN_y products (Figure 3c and Supplementary Figures 16 to 18).

2. Low monomer concentration experiments consisted of a solution of M_1L ($[M_1L]_0 = 10$ nM) triggered by N_3 ($[N_3]_0$ = 10 nM), 24 hours after adding 1 nM of T_{13} into the M_1L solution. These conditions were used during the turnover rate and inhibition assays for the formation of M_1N_3 catalyzed by T_{13} (Figures 2, 3a, and Supplementary Figure 15).

Protocol steps are summarised in Supplementary Table 11 and illustrated in Supplementary Figure 6.

Supplementary Table 11: Detailed experimental procedure to measure dimerization kinetics. Several trajectories with different T_{x3} concentrations and their controls were triggered by mechanical injection and measured simultaneously. The trigger species was N_3. 'Measurement 2' and 'Measurement 4' aimed to estimate the amount of T_{x3} and N_3 in the solution, respectively. However, the estimated concentrations were highly imprecise due to the reactions never completely reaching steady-state.

Steps	Addition	Purpose	Expected concentrations in 200 µL (nm)	Total volume (µL)
**Measurement 1**	10 µL M_xL (2 µM) + 130 µL Buffer	Estimate residual fluorescence baseline	$M_xL = 100$	140
**Measurement 2**	[10,1] µL T_{x3} (100 nM) + [0, 9] µL Buffer	Estimate $[T_{x3}]_0$	$M_xL = [95, 99.75]$; $L = [5, 0.25]$; $M_xT_{x3} = [5, 0.25]$	150
**Measurement 3**	50 µL N_3 (400 nM)	Experimental kinetics	Evolve with time	200
**Measurement 4**	5 µL T_{x3} (200 nM)	Estimate $[N_3 + T_{x3}]$	Equilibrium between M_XN_Y M_xT_{x3} and T_{x3}	205
**Measurement 5**	5 µL each of N_3 and T_{x3} (10 µM)	Estimate $[M_xL]_0$	$M_xN_3 = 100$; $L = 100$; T_{x3} and $N_3 \approx 200$	215

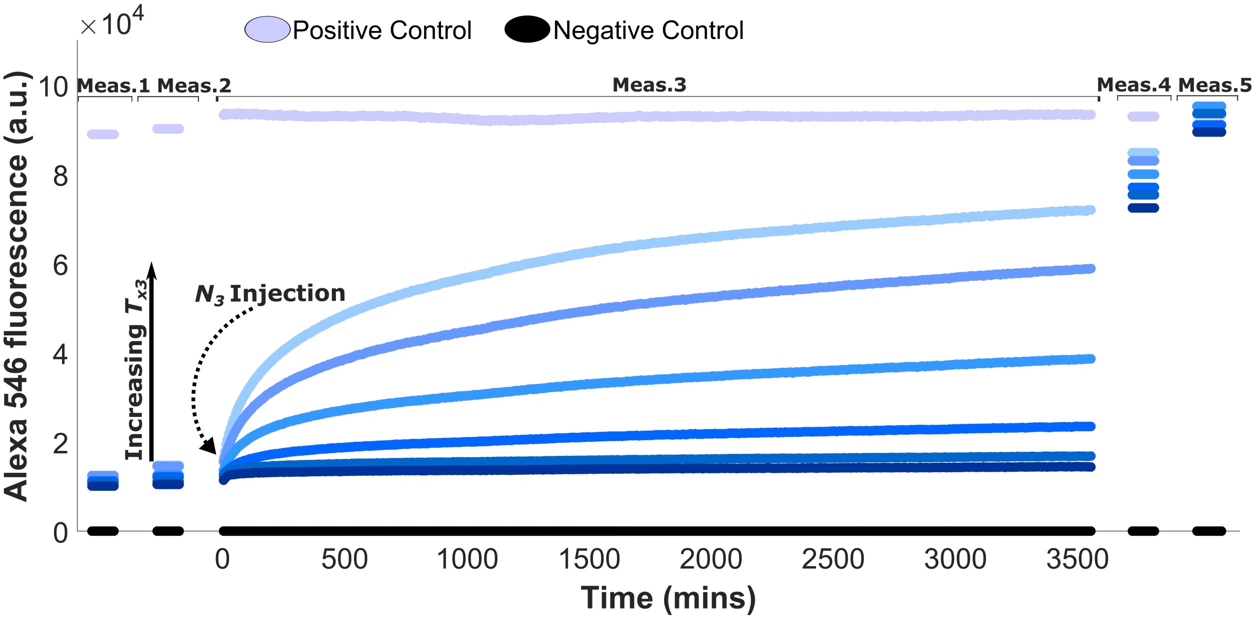

Supplementary Figure 6: Example of dimerization experiments with N_3 (single fluorescent channel). Raw data showing the displacement of L from a M_2L complex by addition of the strands N_3 and T_{23} at 25° C. 'Measurement 1' contains the baseline of the experiments, which are considered as 0 nM of reacted M_2. During 'Measurement 2', variable concentrations of T_{23} produced an increment of the baseline. After that, the complete catalytic cycle was closed by the addition of N_3. After measuring the kinetics for enough time, a small quantity of T_{23} (5 nM) was added to all wells to promote the consumption of all N_3. After one day of further incubation, the new baseline wass measured in 'Measurement 4'. Afterwards the solution was saturated with N_3 and T_{23} to transform all M_2L and quantify the total amount of M_2L in 'Measurement 5'. Concentrations: $[M_2L]_0 = 100$ nM, $[T_{23}]_0 = [0.25, 0.5, 1.25, 2.5, 3.75$ and 5 nM$]$, $[N_3]_0 = 100$ nM. The positive control contained around 100 nM of M_2N_3. The negative controls consisted of only the experimental buffer.

Product inhibition testing. The step-by-step protocol for experiments testing product inhibition are given in Supplementary Table 12. When studying product inhibition, the

experiment included a variable initial concentration of annealed M_1(Unlab.)N_3. This unlabeled product was added before triggering the reaction kinetics with N_3 during 'Measurement 3'. The function of the non-fluorescent M_1N_3 dimer was to inhibit the consumption of the fluorescent M_1L complex via product inhibition, without producing a high fluorescent background that would have hindered the interpretation of the kinetics. The non-fluorescent M_1N_3 was annealed with a 10% excess of N_3 over M_1 (Unlab.) to avoid sequestration of the N_3 strands intended to drive the reaction. Due to the high concentration of inhibitory species in the experiment, N_3 quantification during 'Measurement 4' was unreliable. $[N_3]_0$ was instead assumed to be the intended concentration plus the 10% excess resultant from M_1(Unlab.)N_3 annealing.

Inhibition experiments tested the kinetics with a low monomer concentration ($[M_1L]_0 =$ 10 nM; $[N_3]_0 =$ 10 nM; $[T_{13}\ (ut/vh)]_0 =$ 1 nM). For each (ut/vh) for the template $T_{1,3}$, eight different unlabeled $[M_1N_3]_0$ conditions were tested: 0, 2.5, 5, 10, 15, 25, 50 and 100 nM. A subset of ut/vh was also tested at higher concentrations ($[M_1L]_0 =$ 100 nM; $[N_3]_0 =$ 100 nM; $[T_{13}\ (ut/vh)]_0 =$ 2,5, 5 and 10 nM; $[M_1N_3]_0 =$ 0, 50, 100, 200 and 300 nM).

Supplementary Table 12: Detailed experimental procedure to test product inhibition. Several trajectories with different unlabeled $[M_1N_3]_0$ concentrations and their controls were triggered by mechanical injection and measured simultaneously. The trigger species was N_3. 'Measurement 2' aimed to estimate the amount of T_{x3} in the solution respectively. Due to the excess of product in the solution, no 'Measurement 4' could be included. This table describes experiments at a 10 nM-scale.

**Steps**	**Addition**	**Purpose**	**Expected concentrations in 200 µL (nm)**	**Total volume (µL)**
**Measurement 1**	10 µL M_xL (200 nM) + 130 µL Buffer	Estimate residual fluorescence baseline	$M_xL = 10$	140
**Measurement 2**	10 µL T_{x3} (20 nM)	Estimate $[T_{x3}]_0$	$M_xL = 9$; $L = 1$; $M_xT_{x3} = 1$	150
**Measurement 3**	40 µL N_3 (50 nM) 10 µL M_1N_3 ([0, 2] µM)	Experimental kinetics	Evolve with time	200
**Measurement 5**	5 µL each of N_3 and T_{x3} (10 µM)	Estimate $[M_xL]_0$	$M_xN_3 = 10$; $L = 10$; T_{x3} and $N_3 \approx 200$	210

4.2 Multichannel fluorescence experiments

This section describes experiments that require measuring the direct fluorescence of, and FRET between, several fluorophores. The experiments in question are those in which both M_x and N_y are labeled.

4.2.1 Leak and dimerization experiments with labeled N_1 or N_2

The experiments explored the kinetics of systems consuming monomers N_1 and N_2. The objective, sequence of measurements, and other DNA strands involved in these experiments were similar to their single-fluorophore version involving N_3; the main difference is that fluorescence measurements were performed in multiple channels. Measurements 1 to 3 included measurements in up to three fluorescent channels simultaneously, depending on which fluorophores were present: a channel for M_xL, a channel for N_y, and a channel to follow the FRET signal of the resultant M_xN_y product.

'Measurements 4 and 5' were removed from the experimental protocol. The inferred $[N_y]_0$ could be quantified from its signal just after injection in 'Measurement 3'. 'Measurement 5' was removed due to the difficulty of estimating M_xL through saturation of the system with labeled N_y. If labeled N_y were used to saturate the experiment, the significant increase of signal would interfere with the quantification of the product FRET signal. On the other hand, adding N_3 would produce, besides the assay product, an undetermined amount of product M_xN_3, impeding the quantification of M_xL. Instead, since all experiments were performed at high M_xL concentration, the quenched signal of the complex in 'Measurement 1' was used to infer its initial concentration.

Protocol steps are summarised in Supplementary Table 13 and a multifluorophore dimerization experiment is illustrated in Supplementary Figure 7.

Supplementary Table 13: Detailed experimental procedure for dimerization kinetics with labeled N_y. Several trajectories with different T_{xy} concentrations and their controls were triggered by mechanical injection and measured simultaneously. The trigger species was a labeled N_y strand. During these experiments, 'Measurement 1' was used to estimate the amount of M_xL, while the concentration of N_y was extracted from the initial values of 'Measurement 3'.

Steps	Addition	Purpose	Expected concentrations in 200 µL (nm)	Total volume (µL)
**Measurement 1**	10 µL M_xL (2 µM) + 130 µL Buffer	Estimate $[M_xL]_0$	$M_xL = 100$	140
**Measurement 2**	[10,0.5] µL T_{xy} (100 nM)	Estimate $[T_{xy}]_0$ This step is not used for leak reactions	$M_xL =$ [95, 99.75]; $L =$ [5,0.25]; $M_xT_{xy} =$ [5,0.25]	150
**Measurement 3**	50 µL N_y ([400] nM)	Experimental kinetics and estimate $[N_y]_0$	Evolve with time	200

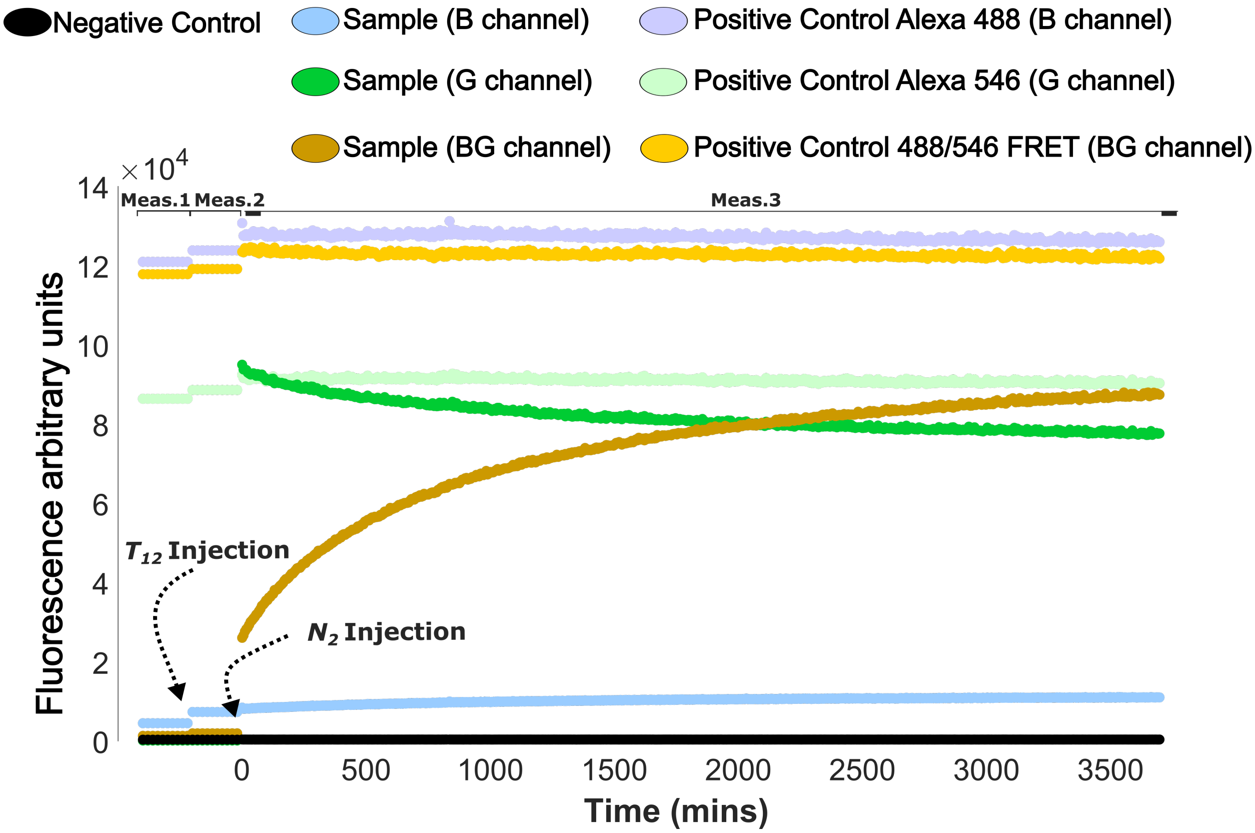

Supplementary Figure 7: Example of dimerization experiments with labeled N_y (multichannel fluorescence experiment). Raw data from the reaction resulting from triggering the release of L from M_1L_1 complexes by adding N_2 and T_{12} at 25° C. 'Measurement 1' contains the baseline of the experiments, considered as 0 nM of reacted M_1. This baseline used to estimate $[M_1L]_0$. During 'Measurement 2', adding T_{12} produces an increment of the baseline that can be quantified. After that, the complete catalytic cycle is closed by adding N_2. The resultant product M_1N_2 produces a FRET signal used to monitor the kinetics. $[N_2]_0$ is estimated from the green signal at the beginning of this kinetic trace. Concentrations: $[M_1L]_0 =$ 100 nM, $[T_{12}]_0 = 5$ nM], $[N_2]_0 = 100$ nM. The positive controls contained around 100 nM of M_1N_3, N_2, or an equimolar mix of both for FRET positive control. The negative controls consisted of only the experimental buffer.

4.2.2 Sequence-specific copying by templated dimerization

The objective of these experiments was both to qualitatively confirm the specificity of templated catalytic assembly and to provide a quantitative estimate of yields. The experiments started with 140 µL containing the three M_xL complexes ($M_{1-3}L$) at a concentration of 100 nM to which 10 µL of solution containing T_{xy} was added, for a final concentration of either 5 nM or 10 nM. The reaction was triggered by adding 50 µL of a solution containing the three species of N_y (N_{1-3}) for a final concentration of 100 nM or 75 nM. Three sets of experiments with slightly different initial concentrations were used but in all cases $[M_xL]_0 \sim [N_y]_0 \gg [T_{xy}]_0$ (refer to Figure 5 and Supplementary Figures 22 and 25 for specific conditions). Each set of experiments contained simultaneously nine systems of interest (one per template), a leak control with a solution containing only the monomers at the same concentration (Supplementary Figure 28), and nine positive controls each containing approximately 75 nM of one of the possible M_XN_y products. For each experiment, fluorescence was monitored in the 6 channels listed in Supplementary Table 8; we discuss the deconvolution of this data in Supplementary Note 5.2. The detailed protocol is described in Supplementary Table 14. An example raw data set of is shown in Supplementary Figure 8.

Supplementary Table 14: Detailed experimental procedure for sequence-specific copying by templated dimerization. For each set, ten wells, each containing all M_xL and a single T_{xy} (or none, in negative control) were triggered by mechanical injection of a solution with all N_y and measured simultaneously in six fluorescence channels. During these experiments, 'Measurement 1' was used to estimate the amount of M_xL, while the concentration of N_y was extracted from the initial values of 'Measurement 3'.

**Steps**	**Addition**	**Purpose**	**Expected concentrations in 200** µL (nm)	**Total volume** (µL)
**Measurement 1**	10 µL $M_{1-3}L$ (2 µM) + 110 µL Buffer	Estimate $[M_xL]_0$	$M_{1-3}L = 100$	140
**Measurement 2**	[10] µL T_{xy} ([100-200] nM)	Estimate $[T_{xy}]_0$	M_xL = [90, 95] (non-complementary $M_xL = 100$); L = [10, 5]; M_xT_{xy} = [5, 0.5]	150
**Measurement 3**	50 µL N_{1-3} ([400-300] nM)	Experimental kinetics and estimate $[N_{1-3}]_0$	Evolve with time	200

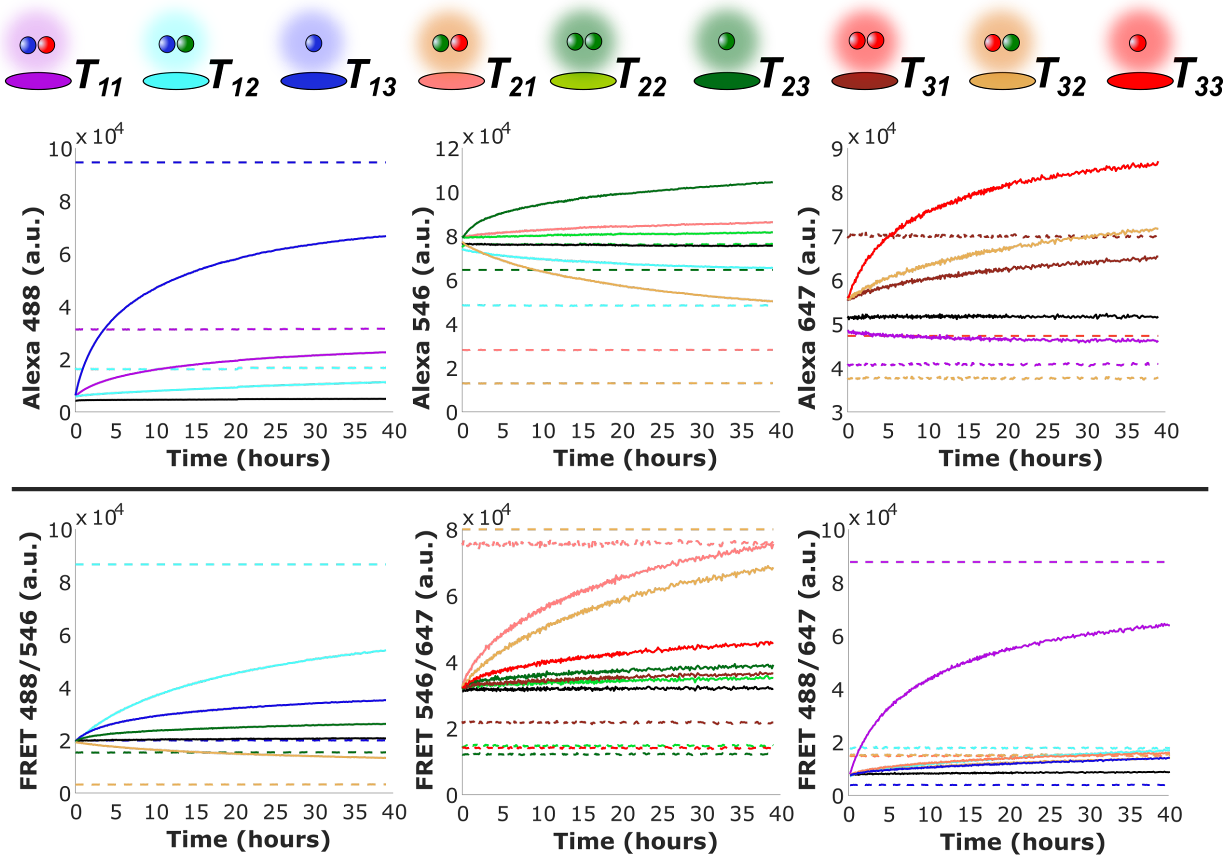

Supplementary Figure 8: Raw data showing the kinetics of the sequence-specific copying by templated dimerization. Fluorescence signal produced in the six measured fluorescence channels for the nine reactions catalyzed by different templates T_{xy}. Only data showing a significant deviation from the leak control is plotted, for clarity. This data is deconvoluted (Supplementary Note 5.2) using the fluorescence calibration matrix in Supplementary Table 7 to obtain estimates of the concentrations of products. This raw data corresponds to the 3rd iteration, with processed data shown in Figure 5 of the main text. Legend: The kinetics of each T_{xy} experiment at the different fluorescence channels are represented by the colors shown in the ovals. The 'halo' of each fluorophore dimer above the ovals indicates the channel at which higher fluorescence contribution is expected for their intended product M_xN_y (Blue, Alexa 488; green, Alexa 546; red, Alexa 647; cyan, FRET 488/546; orange, FRET 546/648; purple, FRET 488/648). Dashed lines: Fluorescence produced by the M_xN_y control corresponding to each template. This control may appear below the kinetics signal due to the background resulting from unconsumed N_y in the kinetic measurements. Black lines: Fluorescence value of the template-free leak control. Trajectories not represented followed the same trend as the leak control. Intended concentrations: Each $[M_xL]_0 = 100$ nM, each $[N_y]_0 = 75$ nM, $[T_{xy}]_0 = 5$ nM.

5 Supplementary Note 5: Data processing

We now discuss how raw fluorescent signals are converted into inferred molecular concentration. Fluorescence signals were processed following a method similar to Cabello *et al.*[7] Subsequent subsections describe how the signal is transformed in each particular set of experiments.

5.1 Single fluorescence channel experiments

5.1.1 Fluorescence coefficient variability

During the recording of the single fluorophore experiments, the bulb of the plate reader had irregular behavior and had to be replaced. Therefore, the absolute calibration reported in Supplementary Note 3 couldn't be used directly to infer concentrations in all cases. Instead, where necessary, we assumed that the initial concentration of M_x (present either in M_xL of M_xT_{xy} complexes) was exactly equal to the intended concentration when averaged over all experiments performed in parallel. We then used the ancillary measurement of total M_x concentration to estimate a coefficient of fluorescence for that complex in the experiment in question. In general, this coefficient differed slightly from the one estimated in Supplementary Note 3. Our second assumption was that all fluorescent species had their coefficient of fluorescence scaled by the same factor relative to the values reported in Supplementary Note 3. In practice, the implied deviation in fluorescence coefficient was typically 10% or less, and the later results (performed with the new bulb) had coefficients consistent with the calibration in Supplementary Note 3 (performed with the new bulb).

5.1.2 Inferring molecular concentrations during displacement of L from M_xL by T_{x3}

Let F_i be the fluorescence at timepoint i during 'measurement 3' for a given reaction, having subtracted the fluorescence of the negative control (buffer only). We first rescale this fluo-

rescence to remove environmental fluctuations evident in the positive control. We do so by rescaling by F_1^+/F_i^+, where F_i^+ is the positive control signal at time i and F_1^+ is the positive control recorded during measurement 1. This $F_i' = (F_1^+/F_i^+)F_i$ is then rescaled again to give a fractional conversion into M_xT_{x3}

$$\frac{[M_xT_{x3}]_i}{[M_xL]_0} = \frac{F_i' - F_1}{\left(F'_{\text{T,max}} - F_1\right)}. \tag{1}$$

Here F_1 is the residual fluorescence during 'Measurement 1' due to the quenched monomers, and $F'_{\text{T,max}}$ is a putative maximal fluorescence if all of the M_x strands were bound to the template. In practice, such a state is not obtained in the experiment; instead, in 'Measurement 5' all monomers are converted to M_xN_3. Let $F'_{\text{N,max}}$ be the (positive control rescaled) fluorescence recorded during 'Measurement 5'. Then

$$[M_xT_{x3}]_i = [M_xL]_0 \frac{F_i' - F_1}{\left(F'_{\text{N,max}}\frac{a_{M_xT_{x3}}}{a_{M_xN_3}} - F_1\right)} = \frac{a_{M_xN_3}}{a_{M_xT_{x3}}}[M_xL]_0 \frac{F_i' - F_1}{\left(F'_{\text{N,max}} - F_1\frac{a_{M_xN_3}}{a_{M_xT_{x3}}}\right)}, \tag{2}$$

where $\frac{a_{M_xN_3}}{a_{M_xT_{x3}}}$ is the ratio of fluorescence coefficients for the two complexes. As outlined above, we assume $\frac{a_{M_1N_3}}{a_{M_1T_{13}}}$ has the value given in Supplementary Section 3.1 (1.37). $\frac{a_{M_xN_3}}{a_{M_xT_{x3}}}$ for $x = 2, 3$ are assumed to be 1, as in those cases the fluorophores showed no variation due to binding with unlabeled strands.

It is assumed that the mean of $F'_{\text{N,max}}$ for all reactions involving the same monomer M_x corresponds to the fluorescence of the intended 100 nM of M_xN_3. The inferred value of $[M_xL]_0$ for each reaction was then calculated by multiplying 100 nM by the value of $F'_{\text{N,max}}$ recorded in that reaction, divided by this mean fluorescence. The total concentration of template, $[T_{x3}]_0$, was inferred from the value recorded during measurement 4, F_4', by replacing F_i' with F_4' in Supplementary Equation 2, and assuming $[T_{x3}]_0 = [M_xT_{x3}]_4$.

5.1.3 Inferring molecular concentrations during the leak reaction between M_xL and N_3

Let F_i be the fluorescence at timepoint i during 'measurement 3' for a given reaction, having subtracted the fluorescence of the negative control (buffer only). We first rescale this fluo-

rescence to remove environmental fluctuations evident in the positive control. We do so by rescaling by F_1^+/F_i^+, where F_i^+ is the positive control signal at time i and F_1^+ is the positive control recorded during measurement 1. This $F_i' = (F_1^+/F_i^+)F_i$ is then rescaled again to give a fractional conversion into M_xN_3

$$\frac{[M_xN_3]_i}{[M_xL]_0} = \frac{F_i' - F_1}{(F_{\rm max}' - F_1)}. \tag{3}$$

Here F_1 is the residual fluorescence during 'Measurement 1' due to the quenched monomers, and $F_{\rm max}'$ is the maximal fluorescence observed during 'Measurement 5', when all monomers M_xL are converted into M_xN_3.

It is assumed that the mean of $F_{\rm max}'$ for all reactions involving the same variant of monomer M_x corresponds to the fluorescence of the intended 100 nM of M_xN_3. The inferred value of $[M_xL]_0$ for each reaction was then calculated by multiplying 100 nM by the value of $F_{\rm max}'$ in that reaction, divided by this mean fluorescence. The inferred value of $[N_3]_0$ was extracted from 'Measurement 4'.

5.1.4 Inferring molecular concentrations during HMSD between M_1T_{13} and N_3

Let F_i be the fluorescence at timepoint i during 'Measurement 3' for a given reaction, having subtracted the fluorescence of the negative control (buffer only). In a slight abuse of notation, we will use F_1, F_2 etc. to refer to specific values of fluorescence obtained in 'Measurement 1', 'Measurement 2' etc., and use F_i for arbitrary time points during 'Measurement 3'.

We first rescale F_i to remove environmental fluctuations evident in the positive control. We do so by rescaling by F_1^+/F_i^+, where F_i^+ is the positive control signal at time i and F_1^+ is the value of the positive control recorded during 'Measurement 1'. This $F_i' = (F_1^+/F_i^+)F_i$ is then rescaled again to give a fractional conversion into M_xN_3

$$\frac{[M_1N_3]_i}{[M_1T_{13}]_0} = \frac{F_i' - F_1}{\left(F_{\rm N,max}' - F_1\right)}. \tag{4}$$

Here F_1 is the residual fluorescence during 'Measurement 1' due to the template-bound monomers, and $F_{\rm N,max}'$ is the putative maximal fluorescence if all of the M_x strands were

bound to N_3. In practice, we infer $F'_{\mathrm{N,max}}$ from F_1, using $F'_{\mathrm{N,max}} = \frac{a_{M_1N_3}}{a_{M_1T_{13}}} F_1$ and the value of $\frac{a_{M_1N_3}}{a_{M_1T_{13}}} = 1.37$ from Supplementary Section 3.1. Thus

$$[M_1N_3]_i = [M_1T_{13}]_0 \frac{F'_i - F_1}{\left(F_1 \frac{a_{M_1N_3}}{a_{M_1T_{13}}} - F_1\right)}. \tag{5}$$

It is assumed that the mean of F_1 for all reactions involving the same variant of M_1 corresponds to the fluorescence of the intended 100 nM of M_1T_{13}. The inferred value of $[M_1T_{13}]_0$ for each reaction was then calculated by multiplying 100 nM by the value of F_1 for that reaction, divided by this mean fluorescence. The total concentration of N_3, $[N_3]_0$, was calculated assuming $[N_3]_0 = [M_1N_3]_4$, the inferred concentration during measurement 4. $[M_1N_3]_4$ was inferred from Supplementary Equation 5 with the fluorescence recorded in measurement 4, F'_4, replacing F'_i.

5.1.5 Inferring molecular concentrations for templated dimerization of M_xL and N_3

The processing of the fluorescence to obtain inferred concentration time series is illustrated in Supplementary Figure 9. Let F_i be the fluorescence at timepoint i during 'measurement 3' for a given reaction, having subtracted the fluorescence of the negative control (buffer only). We first rescale this fluorescence to remove environmental fluctuations evident in the positive control. We do so by rescaling by F_1^+/F_i^+, where F_i^+ is the positive control signal at time i and F_1^+ is the positive control recorded during measurement 1. This $F'_i = (F_1^+/F_i^+)F_i$ is assumed to be the sum of the fluorescence from three species: the product M_xN_3 with fluorescence F_i^*, the template-bound monomer M_xT_{x3} and the initial monomer M_xL.

For simplicity, we assume that during the reaction kinetics (measurement 3) all template strands are bound to M_x. Thus the contribution of M_xT_{x3} to the fluorescent signal should be constant, F'_T. Finally, the contribution of M_xL will be equal to the initial fluorescence recorded in measurement 1, F_1, minus the contribution to that total of the monomers that

have been converted into M_xT_{xy} or M_xN_3.

$$F'_i = F^*_i + F'_T + \left(F_1 - \frac{a_{M_xL}}{a_{M_xN_3}}F^*_i - \frac{a_{M_xL}}{a_{M_xT_{x3}}}F'_T\right). \tag{6}$$

In this expression, $\frac{a_{M_xL}}{a_{M_xN_3}}F^*_i$ estimates the fluorescence contribution of the M_xL complexes that have been converted into M_xN_3, and $\frac{a_{M_xL}}{a_{M_xT_{x3}}}F'_T$ the fluorescence of M_xL complexes that have been converted into M_xT_{xy}. Rearranging,

$$[M_xN_3]_i = \frac{F^*_i}{a_{M_xN_3}} = \frac{F'_i - F'_T\left(1 - \frac{a_{M_xL}}{a_{M_xT_{x3}}}\right) - F_1}{a_{M_xN_3} - a_{M_xL}}. \tag{7}$$

This expression can be simplified using the ancillary measurements. Taking $F'_{\max}$ as the value recorded in the final measurement 5, when all M_x is in M_xN_3 complexes and hence $[M_xN_3] = [M_xL]_0$,

$$[M_xN_3]_i = [M_xL]_0 \frac{F'_i - F'_T\left(1 - \frac{a_{M_xL}}{a_{M_xT_{x3}}}\right) - F_1}{F'_{\max} - F_1}. \tag{8}$$

Continuing in this vein, the value recorded during measurement 2 (F'_2) is assumed to arise from a concentration of monomer-bound templates equal to the injected concentration, $[T_{xy}]_0$, plus the contribution of the remaining M_xL complexes. Thus

$$F'_2 = F'_T + F_1\left(1 - \frac{a_{M_xL}}{a_{M_xT_{x3}}}F'_T\right), \tag{9}$$

or

$$F'_T\left(1 - \frac{a_{M_xL}}{a_{M_xT_{x3}}}\right) = F_1 - F'_2. \tag{10}$$

We therefore infer a product yield of

$$[M_xN_3]_i = [M_xL]_0 \frac{F'_i - F'_2}{F'_{\max} - F_1}. \tag{11}$$

The inferred value of $[M_xL]_0$ for each reaction was calculated by multiplying 100 nM by the value of F_1 for that reaction, divided by this mean of all values of F_1 for the same input (effectively assuming that the mean concentration of $[M_xL]$ over all reactions involving that variant of M_x is equal to the intended value). The injected template concentration, $[T_{xy}]_0$, was inferred as

$$[T_{xy}]_0 = \frac{F'_2 - F_1}{a_{M_xT_{x3}} - a_{M_xL}} = [M_xL]_0 \frac{F'_2 - F_1}{F'_{\max}\frac{a_{M_xT_{x3}}}{a_{M_xN_3}} - F_1}, \tag{12}$$

Using the same assumptions as in the initial displacement of L from M_xL by T_{xy}, $\frac{a_{M_1T_{13}}}{a_{M_1N_3}} = 1/1.37$ and $\frac{a_{M_xT_3}}{a_{M_xN_3}} = 1$ for $x = 2, 3$. However, 'Measurement 2', from which T_{x3} is estimated was highly imprecise, forcing the use of the intended $[T_{x3}]$, rather than a value obtained from 'Measurement 2', during subsequent analysis. Results from 'Measurement 2' were only considered as quality control to check the signal increase as increasing amounts of $[T_{x3}]_0$ were included in the experiment. The low precision during $[T_{x3}]_0$ quantification may have resulted from slow reaction rates and the large relative concentration of M_xL, potentially including some wrongly annealed complexes that mask the signal induced by T_{x3} addition.

$[N_3]_0$ was estimated from transforming the signal in 'Measurement 5' using Supplementary Equation 11 and subtracting the signal expected from 5 nM of T_{x3}.

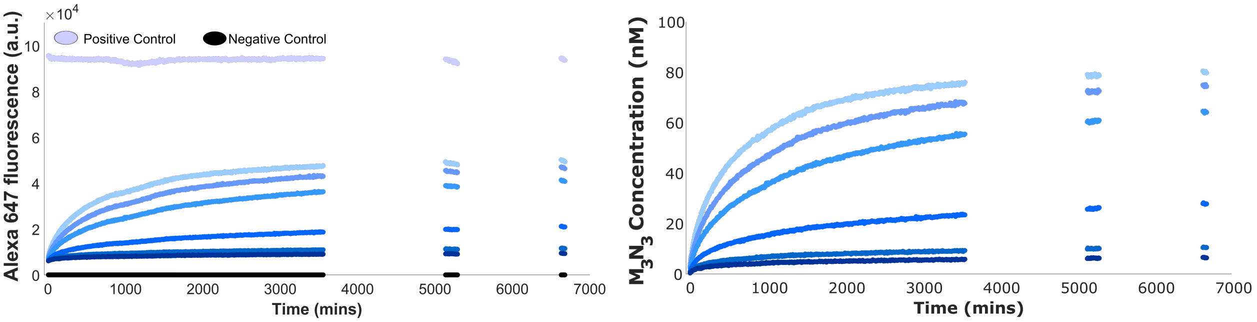

Supplementary Figure 9: Example of the result of fluorescence data processing for single fluorophore experiments. The kinetics contain six traces of the catalytic dimerization experiment of M_3L and N_3 mediated by T_{33}. This case had a particularly non-uniform positive control signal. **Left)** Raw data. During long experiments, plate misalignment can produce bumps in the fluorescence that tampers the fitting process. These artifacts were corrected using the positive control, which has fluorescence intensity fluctuations proportional to those in the samples. **Right)** After the fluorescence processing pipeline. After correcting the fluorescence data it was transformed to nM. $[T_{33}]_0 = [0.25, 0.5, 1.25, 2.5, 3.75, 5]$ nM, $[N_3]_0 = 100$ nM.

5.2 Multichannel fluorescence experiments

Multichannel fluorescent experiments include both assays of a single pair of dye-labeled monomers and their complementary template and assays of sequence-specific dimerization (Supplementary Tables 13 and 14). The data from both classes of experiments were processed in the same way, with only the final fitting to infer concentrations being distinct. In all cases, fittings were performed with the MATLAB R2019a Optimization Toolbox.[5]

In each experiment, a number of fluorescence/FRET channels (ranging from 3 to 6) were monitored. Let F_i^a be the signal observed in channel a at timepoint i of 'Measurement 3', having subtracted the buffer-only negative control. As in Supplementary Section 5.1, we rescale according to $F_i'^{\alpha} = (F_1^{\alpha+}/F_i^{\alpha+})F_i^{\alpha}$, where $F_1^{\alpha+}$ is the positive control signal recorded during 'Measurement 1' in channel a and $F_i^{\alpha+}$ being the positive control at timepoint i during 'Measurement 3'.

In all cases, we consider the increase in fluorescence relative to the value measured during 'Measurement 2', $F_2'^{\alpha}$, as entirely resultant from the injection of labeled N_y and conversion of M_xL and N_y into products M_xN_y; as in Supplementary Note 5.1 the contribution of complexes involving the template is assumed to be constant during 'Measurement 3' (and equal to the value obtained during 'Measurement 2'). Thus within channel α

$$\Delta F_i^{\prime,\alpha} = \sum_y \left([N_y]_0 - \sum_x [M_xN_y]_i \right) a_N^{\alpha} + \sum_{x,y} [M_xN_y]_i \left(a^{\alpha}_{M_xN_y} - \alpha^{a}_{M_xL} \right), \qquad (13)$$

where $\Delta F_i^{\prime,\alpha} = F_i^{\prime,\alpha} - F_2^{\prime,\alpha}$, a_Z^{α} is the coefficient of fluorescence of species Z in channel α, as collected in Supplementary Table 8. We estimate $[M_xN_y]_i$ at each timepoint i by fitting Supplementary Equation 13 to the data.

5.2.1 Leak and dimerization experiments with labeled N_1 or N_2

During these assays, up to three fluorescent channels were monitored, corresponding to the fluorophore on M_x, the fluorophore on N_y, and the FRET channel for M_xN_y (for M_2N_2 and M_3N_1, the FRET channel doesn't exist as the fluorophores are identical). With a single monomer of each type, Supplementary Equation 13 reduces to

$$\Delta F_i^{\prime,\alpha} = ([N_y]_0 - [M_xN_y]_i) \, a_N^{\alpha} + [M_xN_y]_i \left(a^{\alpha}_{M_xN_y} - \alpha^{a}_{M_xL} \right). \qquad (14)$$

$[M_xL]_0$ was inferred by dividing the signal obtained in its dominant channel during 'Measurement 1' by the fluorescence coefficient: $[M_xL]_0 = F_1^{\alpha}/a^{\alpha}_{M_xL}$. $[N_y]_0$ was inferred by assuming $[M_xN_y] = 0$ immediately after the injection that starts 'Measurement 3'. The increase in fluorescence of the first timepoint of 'Measurement 3' relative to 'Measurement 2' ($\Delta F_3^{\prime,\alpha}$)

in the channel most relevant to N_3 was then used to infer $[N_y]_0$ via $[N_y]_0 = (\Delta F_3^{\prime,\alpha})/a_{N_y}^{\alpha}$.

$[M_xN_y]$ at subsequent timepoints was inferred by fitting $[M_xN_y]_i$ to the observed values of $\Delta F_i^{\prime,\alpha}$ by minimizing the base-10 logarithm of the mean square error:

$$\log_{10}\left(\frac{\sum_{\alpha=1}^{3}(\Delta F_{i,\mathrm{meas}}^{\prime,\alpha} - \Delta F_{i,\mathrm{pred}}^{\prime,\alpha})^2}{w_\alpha}\right), \tag{15}$$

which quantifies the difference between the observed signal and the signal predicted using Supplementary Equation 14 in the various channels. Here, $\Delta F_{i,\mathrm{meas}}^{\prime,\alpha}$ is the measured increase in fluorescence in channel α at time step i relative to 'Measurement 2', and $\Delta F_{i,\mathrm{pred}}^{\prime,\alpha}$ is the increase predicted using Supplementary Equation 14. To reduce the impact of calibration and assumption-originated errors, the fluorescence channels were weighted differently using a factor w_α to prioritize error reduction in the FRET-measuring channel signal. The fit was performed chronologically through the timepoints, with the initial condition for each timepoint being $[M_xN_y]_{i-1}$, the value obtained at the previous timepoint.

5.2.2 Sequence-specific copying by templated dimerization

The procedure is similar to inferring the concentrations of a single product, except that all six FRET/fluorescence channels were monitored in each experiment, and the aim is to extract concentrations for all nine products M_xN_y. In practice, it is essentially impossible to infer M_2N_2 because $\alpha_{N_2} + \alpha_{M_2} \approx \alpha_{M_2N_2}$ in the channel in which it generates a strong signal. Formation of M_3N_1, which also contains two identical fluorophores (Alexa Fluor 647), however, results in a detectable change in fluorescence, albeit with a low signal-to-noise ratio. We therefore neglect the $[M_2N_2]$ terms in Supplementary Equation 13, and fit only the remaining eight values of $[M_xN_y]$ to the observed data.

Fitting proceeded as follows:

1. From the signal of 'Measurement 1' we estimated $[M_1L]_0$, $[M_2L]_0$ and $[M_3L]_0$ by fitting the signal of the 6 fluorescence channels, using as $a_{M_xL}^{\alpha}$ their corresponding values from the fluorescent coefficient calibration matrix (Supplementary Table 7 or 8). We fit

initial concentrations to the fluorescence data by minimizing:

$$\log_{10}\left(\frac{\sum_{\alpha=1}^{6}(F^{\alpha}_{1,\mathrm{meas}} - F^{\alpha}_{1,\mathrm{pred}})^2}{6}\right), \tag{16}$$

given $F^{\alpha}_{1,\mathrm{pred}} = \sum_x [M_xL]_0 a^{\alpha}_{M_xL}$, with initial estimates for $[M_xL]_0$ given by their intended concentration. Due to the low signal of the quenched $[M_xL]_0$, the quantification of this species was more prone to error than other fluorescent species.

2. We inferred $[N_1]_0$ and $[N_2]_0$ from the first timepoint of 'Measurement 3' by minimizing

$$\log_{10}\left(\frac{\sum_{\alpha=1}^{6}(F'^{\alpha}_{3,\mathrm{meas}} - \Delta F'^{\alpha}_{3,\mathrm{pred}})^2}{6}\right), \tag{17}$$

given $\Delta F'^{,\alpha}_{3\mathrm{pred}} = \sum_{y=1,2}[N_y]_0 a^{\alpha}_{N_y}$, and using the intended concentrations as initial conditions of the fit. Since $[N_3]_0$ is not labeled, but it was injected in a mix along the other two monomers, its concentration for each well was assumed to be the average of the fitted $[N_1]_0$ and $[N_2]_0$.

3. We iteratively fitted $[M_xN_y]_i$ at each successive timepoint i to minimise

$$\log_{10}\left(\frac{\sum_{\alpha=1}^{6}(\Delta F'^{\alpha}_{i,\mathrm{meas}} - \Delta F'^{\alpha}_{i,\mathrm{pred}})^2}{6} + 5000\frac{\sum_{x,y\neq 2,2}([M_xN_y]_i - [M_xN_y]_{i-1})^2}{8}\right). \tag{18}$$

Here, $\Delta F'^{,\alpha}_{i,\mathrm{meas}}$ is the measured increase in fluorescence in channel α at time step i of 'Measurement 3' relative to 'Measurement 2', and $\Delta F'^{,\alpha}_{i,\mathrm{pred}}$ is the increase predicted using Supplementary Equation 13, given the estimated $[M_xN_y]_i$ at that timepoint. $[M_xN_y]_{i-1}$ are the concentrations estimated at the previous time step. $[M_xN_y]_{i-1}$ was also used as the initial condition for the fitting of $[M_xN_y]_i$, except for the dimer that matches the template. The initial condition for that matching dimer was chosen by incrementing $[M_xN_y]_i$ sufficiently to match the increase in fluorescence in the most relevant fluorescence/FRET channel.

Since there are eight variables to fit six signals, a unique optimal fit cannot be guaranteed. However, using the iterative procedure outlined above that penalizes unphysical rapid concentration fluctuations, it was possible to obtain a well-behaved fit to the fluorescent signals.

6 Supplementary Note 6: Fitted models

We fit the molecular concentrations, inferred as described in Supplementary Note 5, to simple kinetic models.

Binding of M_xL to T_{xy}. Binding of M_xL to T_{xy} (or displacement of the lock strand L from M_xL by T_{xy}) is fitted using an irreversible reaction:

$$\mathrm{M}_x\mathrm{L} + \mathrm{T_{xy}} \xrightarrow{\mathrm{k}_t} \mathrm{M}_x\mathrm{T_{xy}}. \tag{19}$$

The corresponding ODE is

$$\frac{d[M_xT_{xy}]}{dt} = k_t[M_xL][T_{xy}], \tag{20}$$

using initial conditions $[M_xT_{xy}]_0 = 0$ and $[M_xL]_0$ and $[T_{xy}]_0$ inferred from the data as outlined in Supplementary Note 5.

Leak reaction between free-in-solution M_xL and N_y. Reaction between monomers in the absence of T_{xy} is fitted using an irreversible reaction:

$$\mathrm{M}_x\mathrm{L} + \mathrm{N}_y \xrightarrow{\mathrm{k_{leak}}} \mathrm{M}_x\mathrm{N}_y. \tag{21}$$

The corresponding ODE is

$$\frac{d[M_xN_y]}{dt} = k_{\text{leak}}[M_xL][N_y], \tag{22}$$

with initial conditions $[M_xN_y]_0 = 0$ and $[M_xL]_0$ and $[N_y]_0$ inferred from the data as outlined in Supplementary Note 5.

Handhold-mediated strand displacement of T_{xy} by N_y. The displacement of T_{xy} from the M_xT_{xy} complex is fitted using an irreversible reaction:

$$\mathrm{M}_x\mathrm{T_{xy}} + \mathrm{N}_y \xrightarrow{\mathrm{k}_h} \mathrm{M}_x\mathrm{N}_y/\mathrm{T_{xy}}. \tag{23}$$

With '/' denoting the handhold-mediated binding of M_xN_y to T_{xy}. The corresponding ODE is

$$\frac{d[M_xN_y/T_{xy}]}{dt} = k_h[M_xT_{xy}][N_y], \tag{24}$$

with initial conditions $[M_xN_y/T_{xy}]_0 = 0$ and $[M_xT_{xy}]_0$ and $[N_y]_0$ inferred from the data as outlined in Supplementary Note 5.

Initial turnover frequency for catalytic dimerisation of M_xL and N_y by T_{xy}. We assume that at short times (less than two hours for 10 nM M_xL-scale reactions and thirty minutes for 100 nM M_xL-scale reaction) the concentration of reacted M_xL molecules (inferred as outlined in Supplementary Note 5) is linear with time

$$[M_xL]_{\text{reacted}} = V_0 t + c. \tag{25}$$

Here, V_0 is the initial rate of the reaction, which is divided by the intended amount of template in the reaction to calculate the turnover frequency (TOF).

7 Supplementary Note 7: General fitting procedure for the estimation of rate constants

All fits were performed, using MATLAB® R2022b 'Optimization Toolbox', on the data transformed as described in Supplementary Section 5.

7.1 Fits to obtain k_t, k_h and k_{leak}

Data from individual reactions were fitted to the analytical solutions of the ODE models (Supplementary Equations 19, 23 and 21),

$$[X]_t = [\text{Trig}]_0 \left(\frac{1 - e^{k(t+t_0)([\text{Trig}]_0 - [X^*]_0)}}{1 - \frac{[Trig.]_0}{[X^*]_0} e^{k(t+t_0)([\text{Trig}]_0 - [X^*]_0)}} \right), \tag{26}$$

with X being the reaction-produced fluorescence species, X^* the initially quenched species, k the 2nd order kinetic rate of the reaction and Trig the species at the lower concentration used to trigger the reaction, e.g., N_{xy} in HMSD reactions. We used the minimization function *fminsearch* to minimize the base-10 logarithm of the weighted mean squared errors of the fitting

$$\log_{10}(\text{ mean weighted error }) = \log_{10} \frac{\sum_t \left([X]_{\text{exp}}(t) - [X]_{\text{model}}(t)\right)^2}{[X]_{\text{model}}(t)}, \tag{27}$$

where $[X]_{\text{exp}}(t)$ is the inferred concentration of product at time t of the fluorescent trace, and $[X]_{\text{model}}(t)$ is the total concentration predicted by the model at time t. The error was weighted at each point with $[X]_{\text{model}}(t)$ to account for the Poisson distribution of the photomultiplier detection error.

We treated the rate constant k as the main fitting parameter. The initial guess for k (k_{init}) was inferred from the time at which the reaction reached 90% completion:

$$k_{\text{init}} = \frac{\ln\left(10 - 9\frac{[\text{Trig.}]_0}{[X]_0}\right)}{t_{90\%}\left([X]_0 - [\text{Trig}]_0\right)}. \tag{28}$$

In addition, in the fits to Supplementary Equation 26, the initial measurement time (t_0) and $[\text{Trig}]_0$ were treated as secondary parameters to be fitted. The initial guess for t_0 (t_{0_init})

was obtained from the cutting point with the x-axis of projection of the initial rate of the reaction. This projection was a linear fit calculated using initial data from before the reaction had reached 50% completion, where the trajectories are in a pseudo-linear regime.

Approximate values of $[\text{Trig}]_0$ were inferred as side products from the processing of experimental data outlined in Supplementary Notes 4 and 5, typically using the steady-state plateau in 'Measurement 4'. These approximate values were then used as initial conditions for fitting $[\text{Trig}]_0$ as part of identifying rate constants k. For kinetics that did not reach at least 90% completion, the trigger's concentration was only allowed to fluctuate $\pm$ 10% from the value inferred from 'Measurement 4'. The percentage of reaction completion was determined by comparing the last value of the recorded kinetics in 'Measurement 3' with 'Measurement 4'. Consequently, for leak reactions, which never reach completion in the measured time, $[N_y]_0$ is always only allowed to fluctuate $\pm$ 10%.

7.1.1 Results for M_xL binding to T_{xy} (estimating k_t)

The fitted k_t values are reported in Supplementary Table 15. Fitted trajectories are shown in Supplementary Figures 10 and 11. The inferred rate constants presented some variability between different tested T_{xy} strands that shared a t sequence. This difference could perhaps be explained by experimental error or the partial secondary structure of the T_{xy} strands.

Supplementary Table 15: Fitted TMSD rate constant k_t. Data obtained using procedures described in Supplementary Table 9 and Supplementary Note 7. The reactant strands used, at 25° C, were all T_{xy} strands with 8 nt h and 6 nt primary toehold unless stated otherwise, (Supplementary Table 1) with their complementary M_x (Supplementary Table 2). Mean of all fitted k_t for six-nucleotide toehold variants of $M_1 = 9.1\pm 0.3$ x 10^4 M^{-1} s^{-1}; for $M_2 = 4.3\pm 0.3$ x 10^4 M^{-1} s^{-1}; for $M_3 = 2.33\pm 0.12$ x 10^4 $M^{-1}s.^{-1}$

**Condition (x/y) (ut/vh)**	**Individual experiments k_t x 10^5 (M^{-1} s^{-1})**									**Mean k_t x 10^5 ± SEM (M^{-1} s^{-1})**
T_{11}	0.99	0.9	0.84	0.79	0.75	0.75	0.74	0.75	-	0.81 ± 0.03
T_{12}	0.885	0.9	0.979	0.995	0.985	0.943	0.921	0.916	0.858	0.931 ± 0.016
T_{13}	1.1	1.1	1.12	1.21	0.92	1.03	0.6	0.71	0.92	0.97 ± 0.07
T_{21}	0.24	0.233	0.235	0.234	0.24	0.245	0.256	0.278	0.347	0.256 ± 0.012
T_{22}	0.501	0.473	0.486	0.486	0.492	0.503	0.5	0.526	0.57	0.504 ± 0.01
T_{23}	0.671	0.545	0.553	0.519	0.497	0.52	0.535	0.508	0.586	0.548 ± 0.018
T_{31}	0.1674	0.1699	0.1729	0.1741	0.1756	0.1739	0.1758	0.1785	0.1829	0.1746 ± 0.0015
T_{32}	0.3165	0.3082	0.3196	0.3105	0.3147	0.3151	0.3127	0.3144	0.3172	0.3143 ± 0.0012
T_{33}	0.222	0.209	0.207	0.208	0.21	0.209	0.221	0.217	0.207	0.212 ± 0.002
T_{13} **(4t/8h)**	0.0113	0.011	0.01148	0.0111	-	-	-	-	-	0.01122 ± 0.00011
T_{13} **(5t/8h)**	0.0449	0.0444	0.0442	0.0456	-	-	-	-	-	0.0448 ± 0.0003
T_{13} **(7t/8h)**	9.6	10.7	11.2	10.8	10.5	10.4	-	-	-	10.5 ± 0.2
T_{13} **(8t/8h)**	13.7	15.4	18.5	16.4	16.1	16.1	-	-	-	16 ± 0.6

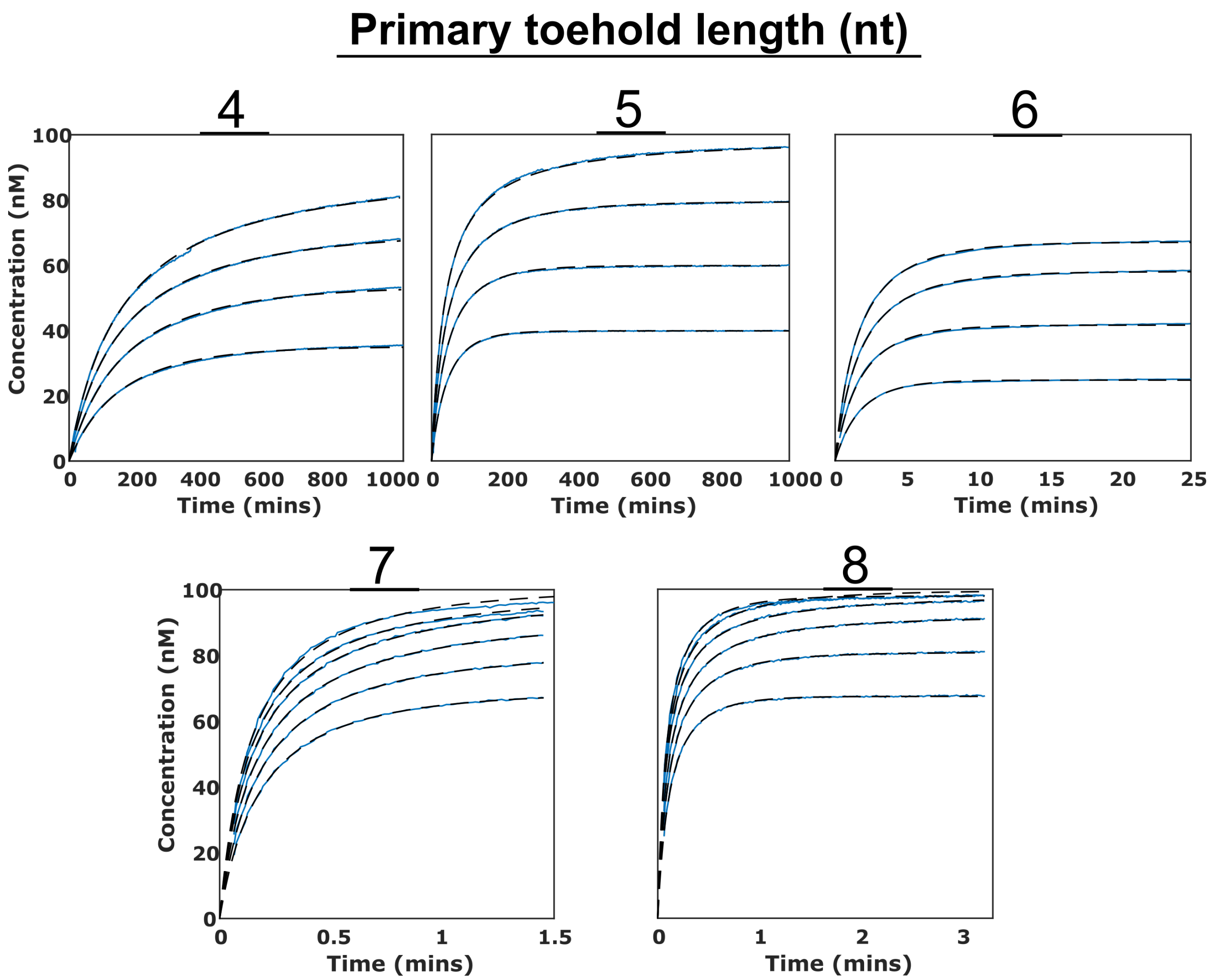

Supplementary Figure 10: Fits of k_t for different primary toehold lengths t. The kinetics display the concentration of displaced M_1L with time. Blue: Experimental data. Dashed line: Fitted model. Intended initial concentrations: $[M_1L]_0 = 100$ nM, $[T_{xy}]_0 =$ variable.

7.1.2 Results for leak reaction rates k_{leak} for M_xL and N_y

Of these systems, only M_1L with N_3 or N_1 were assayed with replicas as independent experiments. The rest of monomer combinations were individual controls accompanying their corresponding templated reactions. Due to the subtle fluorescence changes when producing M_2N_2 and M_3N_1 from their constituents, no leak signal could be detected and hence no k_{leak} could be fitted. For fitting k_{leak}, the initial 1000 or 2000 minutes of the reaction were ignored to fit the representative kinetics at later time, and thus neglecting the initial leak due to malformed structures.

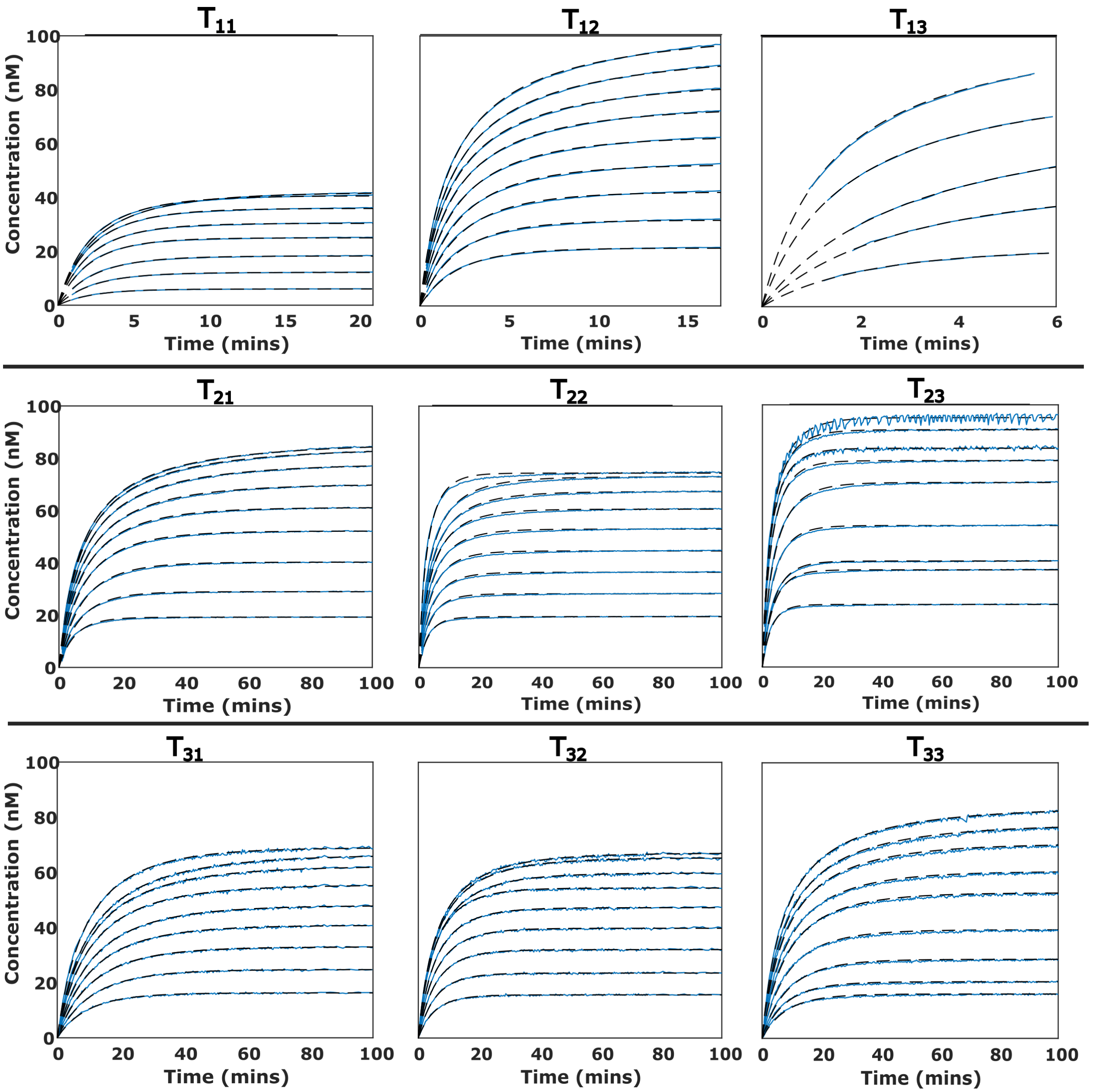

Supplementary Figure 11: Fits of k_t for different competing templates. The kinetics display the concentration of displaced M_xL with time. Blue: Experimental data. Dashed line: Fitted model. Intended concentrations: $[M_xL]_0$ = 100 nM, $[T_{xy}]_0$ = variable.

All fitted k_{leak} are collected in Supplementary Table 16. Fitted trajectories are shown in Supplementary Figure 12. The obtained k_{leak} values are very slow in comparison with the overall templated reaction, having a value that ranges from the 10^{-1} to 1 M^{-1} s^{-1}. Additionally, k_{leak} seems more dependent on M_xL than N_y, with averages for each M_x being: $M_1 = 0.32$ ± 0.05 M^{-1} s^{-1}; $M_2 = 1.1$ ± 0.2 M^{-1} s^{-1}; $M_3 = 0.56$ ± 0.013 M^{-1} s^{-1}.

Supplementary Table 16: Fitted leak reaction rate k_{leak}. Data obtained using procedures described in Supplementary Table 10 and Supplementary Note 7. The reactant complexes used were M_xL (Supplementary Table 2) triggered by different N_y strands (Supplementary Table 3). All reported experiments were performed at 25^{0}C. Mean of fitted k_{leak} for $M_1L = 0.32 \pm 0.05$ M^{-1} s^{-1}; $M_2L = 1.1 \pm 0.2$ M^{-1} s^{-1}; $M_3L = 0.56 \pm 0.013$ M^{-1} s^{-1} .

Condition (x/y)	Individual experiments k_{leak} (M^{-1} s^{-1})					Mean k_{leak} ± SEM (M^{-1} s^{-1})
M_1N_1	0.17	0.31	0.65	0.22	-	0.34 ± 0.1
M_1N_2	0.25	-	-	-	-	0.25
M_1N_3	0.22	0.25	0.32	0.56	0.27	0.32 ± 0.06
M_2N_1	1.2	-	-	-	-	1.2
M_2N_3	0.9	-	-	-	-	0.9
M_3N_2	0.55	-	-	-	-	0.55
M_3N_3	0.57	-	-	-	-	0.57

7.1.3 Results for handhold-mediated strand displacement reaction rate k_h for the reaction of M_1T_{13} with N_3

All fitted k_h are collected in Supplementary Table 17. Fitted trajectories are shown in Supplementary Figures 13 and 14. The fitted experiments had a lower signal-to-noise ratio and were faster than experiments that started with fully quenched monomers, leading to k_h values with significant uncertainty. However, the results show a clear trend of increasing k_h with handhold length. Additionally, several primary toehold lengths were tested with handhold 8h, to test if the fluorescence recovery was correlated with the detachment of the product from T_{xy}. No importantt differences in fluorescence recovery were observed between experiments.

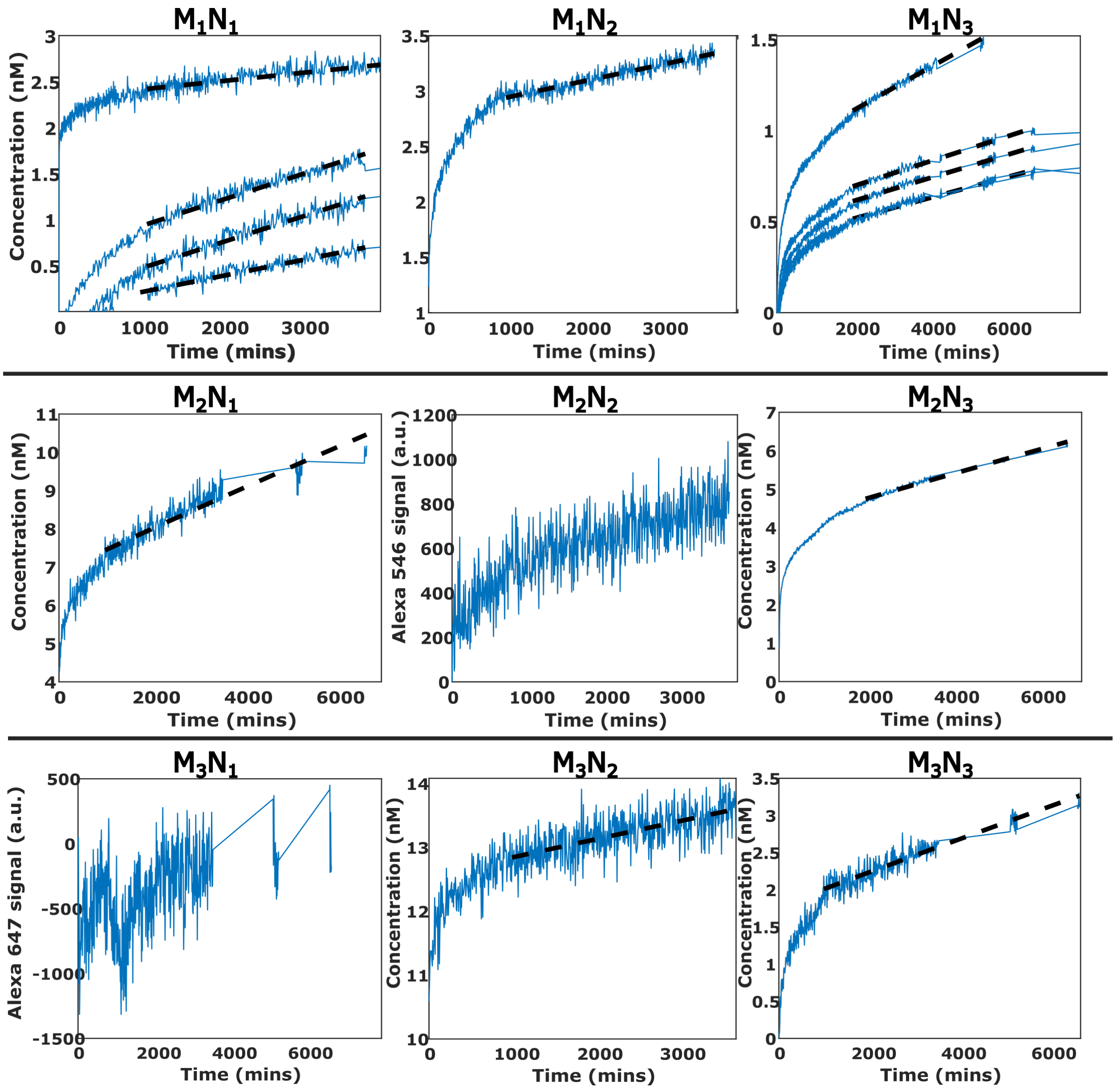

Supplementary Figure 12: Fits of k_{leak} for different combination of monomers. Kinetics display the concentration of displaced M_xL with time, except in M_2N_2 and M_3N_1 where the processed values of fluorescence are reported. Blue: Experimental data. Dashed line: Fitted model. Intended concentrations: $[M_xL]_0 = 100$ nM, $[N_y]_0 = 100$ nM (or 100, 80, 60 and 40 nM for M_1N_3). M_1N_1 concentrations were: 100/100, 100/100, 150/150 and 50/150 (M_x/N_y nM).

Supplementary Table 17: Fitted k_h. Data obtained using procedures described in Supplementary Table 10 and Supplementary Note 7. The reactant complexes used were M_1T_{13} (Supplementary Tables 1 and 2) triggered by N_3 strands (Supplementary Table 3). Fitting results with excessive error, which were not considered for the mean calculation in the final solumn, are shown in italics. Mean of fitted k_h for the values considered reliable: 6 nt handhold: 5.9 ± 0.6 x 10^4 M^{-1} s^{-1}; 7 nt: 5.2 ± 0.4 x 10^4 M^{-1} s^{-1}; 8 nt: 8 ± 0.5 x 10^4 M^{-1} s^{-1}; 9 nt: 2.26 ± 0.1 x 10^5 M^{-1} s^{-1}; 10 nt: 5.6 ± 0.8 x 10^5 M^{-1} s^{-1}

**Condition (*ut*/*vh*)**	**Individual experiments k_h x 10^4 (M^{-1} s^{-1})**						**Mean k_h x 10^4 ± SEM (M^{-1} s^{-1})**
**6/6**	*16.3*	*15*	*12.3*	*10.3*	6.5	5.3	5.9 ± 0.6
**6/7**	*17.3*	*15.7*	*12.4*	5.5	4.3	5.9	5.2 ± 0.4
**4/8**	3.2	3.58	3.3	3.75	4.06	3.86	3.63 ± 0.13
**5/8**	10.4	8.8	8.6	8.1	8.5	7.9	8.7 ± 0.4
**6/8**	7.14	7.28	6.74	6.82	7.41	7.86	7.21 ± 0.17
**7/8**	9.8	9.5	9.5	10.2	11.3	12.3	10.4 ± 0.5
**8/8**	9.7	9.3	10.2	11.3	11.2	6.9	9.8 ± 0.7
**6/9**	22.2	23.5	21.1	26.3	19	23.4	22.6 ± 1
**6/10**	64	66	*10*	*17*	*26*	40	56 ± 8

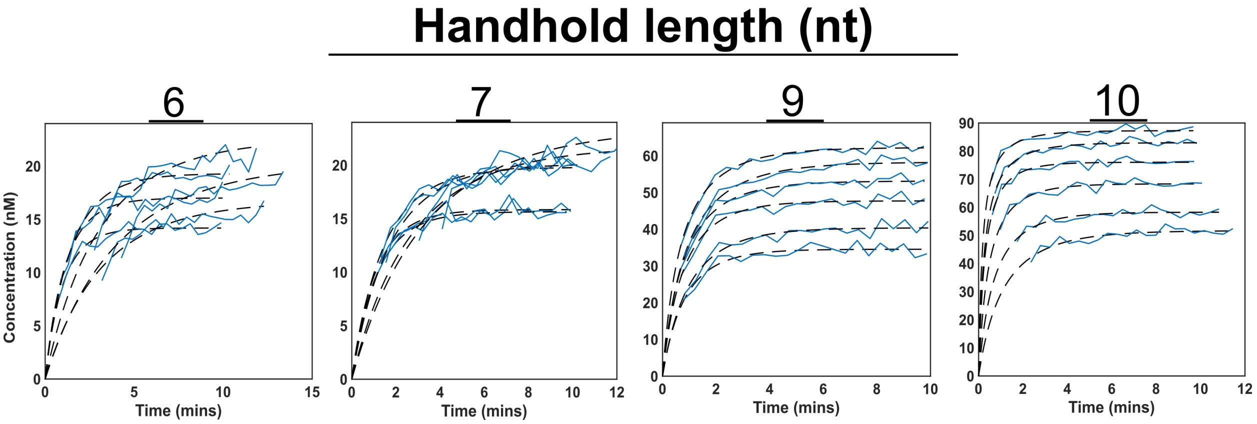

Supplementary Figure 13: Fits of k_h for different handhold lengths. Kinetics display the concentration of formed M_1N_3 with time. Due to the low signal-to-noise ratio and swiftness of the reaction, most of the kinetics were almost completed when starting to read. Blue: Experimental data. Dashed line: Fitted model. Intended concentrations: $[M_1T_{13}]_0 = 100$ nM, variable concentration for N_3.

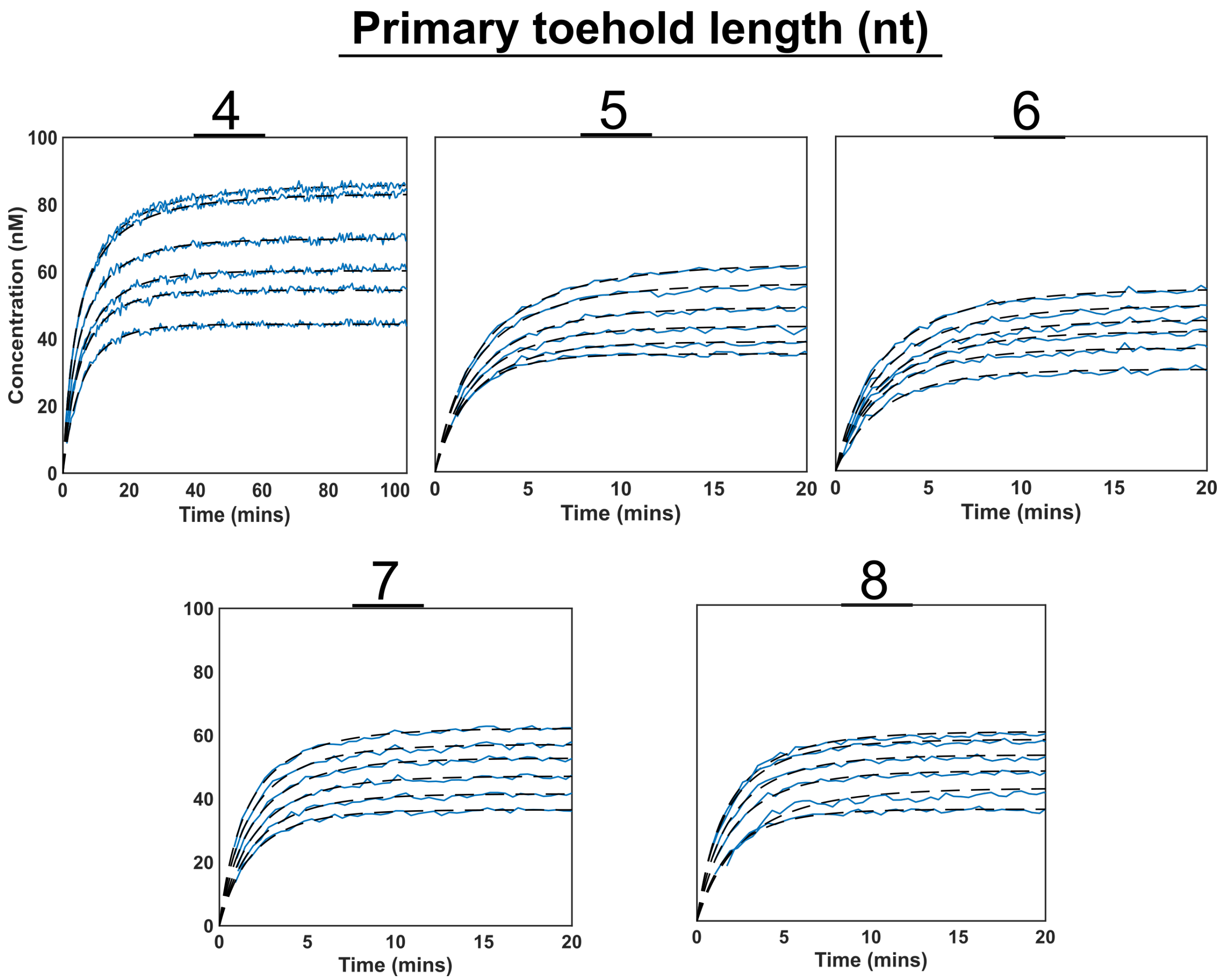

Supplementary Figure 14: Primary toehold length doesn't affect the HMSD kinetics. Fitting of 8 nt handhold experiments with different t lengths. Kinetics display the concentration of formed MN_xL with time. Blue: Experimental data. Dashed line: Fitted model. Intended concentrations: $[M_1T_{13}]_0 = 100$ nM, variable concentration for N_3.

7.2 Fitting of turnover frequency for catalytic dimerization of $\boldsymbol{M_1L}$ and $\boldsymbol{N_3}$ mediated by $\boldsymbol{T_{13}}$ with and without product inhibition

The first minutes of each individual reaction were fitted to Supplementary Equation 25, weighting each timepoint by the inverse of its fluorescence intensity. In this section, we report the slope of the regression line, which corresponds to the reactions' initial rate, with a confidence interval of 95%.

All fitted initial rates are collected in Supplementary Table 18. Complete trajectories are shown in Supplementary Figure 15. From the reported results, an IC_{50} was estimated for each condition by simple linear interpolation between the two points nearest to half the initial rate of the reactions at $[M_1N_3]_0$=0. For example, IC_{50}(6t/6h) ≈ 70 nM, IC_{50}(6t7h) ≈ 50 nM, IC_{50}(6t8h) ≈ 11 nM, IC_{50}(6t9h) ≈ 4 nM, and IC_{50}(6t10h) ≈ 2 nM.

Supplementary Table 18: Initial rates of monomer turnover by T_{13} in the presence of M_1N_3 (10 nM-scale). Data obtained using procedures described in Supplementary Table 18 and Supplementary Note 7. The reactant complexes used were M_1L (Supplementary Table 1) triggered by N_3 strands (Supplementary Table 3) in the presence of different T_{13} strands (Supplementary Table 1). Intended concentrations: $[M_1L]_0$ = 10 nM, $[N_3]_0$ 10 nM, $[T_{13}]_0$ = 1 nM, $[M_1N_3]_0$ = variable. Fitting results are reported in nM hour^{-1}, with a 95% confidence interval.

**Condition** (*ut*/*vh*)	$[M_1N_3]_0$ **0 nM**	$[M_1N_3]_0$ **2.5 nM**	$[M_1N_3]_0$ **5 nM**	$[M_1N_3]_0$ **10 nM**	$[M_1N_3]_0$ **15 nM**	$[M_1N_3]_0$ **25 nM**	$[M_1N_3]_0$ **50 nM**	$[M_1N_3]_0$ **100 nM**
T_{13} **(4t/6h)**	0.024±0.004	0.017±0.007	0.016±0.005	0.021±0.005	0.023±0.004	0.018±0.004	0.021±0.005	0.021±0.005
T_{13} **(4t/7h)**	0.025±0.005	0.024±0.004	0.021±0.005	0.021±0.003	0.02±0.004	0.025±0.005	0.021±0.005	0.02±0.006
T_{13} **(4t/8h)**	0.046±0.006	0.049±0.008	0.056±0.008	0.043±0.005	0.054±0.009	0.05±0.008	0.051±0.008	0.036±0.007
T_{13} **(4t/9h)**	0.043±0.007	0.037±0.009	0.035±0.008	0.033±0.006	0.029±0.007	0.022±0.006	0.061±0.014	0.014±0.006
T_{13} **(4t/10h)**	0.05±0.007	0.037±0.007	0.035±0.007	0.032±0.005	0.033±0.006	0.028±0.005	0.017±0.009	0.024±0.005
T_{13} **(5t/6h)**	0.027±0.004	0.027±0.005	0.026±0.005	0.022±0.006	0.018±0.005	0.021±0.004	0.021±0.004	0.02±0.006
T_{13} **(5t/7h)**	0.028±0.005	0.026±0.004	0.029±0.005	0.028±0.003	0.028±0.005	0.024±0.004	0.026±0.005	0.018±0.004
T_{13} **(5t/8h)**	0.134±0.012	0.119±0.012	0.129±0.014	0.108±0.013	0.099±0.01	0.099±0.013	0.089±0.012	0.081±0.011
T_{13} **(5t/9h)**	0.105±0.008	0.081±0.01	0.082±0.01	0.069±0.009	0.049±0.009	0.058±0.009	0.031±0.008	0.029±0.012
T_{13} **(5t/10h)**	0.092±0.006	0.065±0.005	0.062±0.006	0.042±0.007	0.043±0.006	0.038±0.007	0.032±0.007	0.034±0.01
T_{13} **(6t/6h)**	0.063±0.004	0.063±0.005	0.056±0.004	0.05±0.005	0.049±0.006	0.044±0.005	0.037±0.005	0.022±0.006
T_{13} **(6t/7h)**	0.096±0.005	0.082±0.004	0.08±0.003	0.074±0.005	0.066±0.004	0.063±0.005	0.046±0.005	0.019±0.005
T_{13} **(6t/8h)**	0.622±0.009	0.493±0.008	0.417±0.009	0.322±0.011	0.261±0.013	0.223±0.012	0.155±0.014	0.058±0.007
T_{13} **(6t/9h)**	1.01±0.02	0.615±0.015	0.453±0.013	0.307±0.008	0.208±0.006	0.161±0.009	0.09±0.009	0.067±0.009
T_{13} **(6t/10h)**	0.369±0.013	0.156±0.01	0.101±0.007	0.071±0.006	0.064±0.011	0.048±0.008	0.043±0.01	0.041±0.01
T_{13} **(7t/6h)**	0.058±0.003	0.056±0.003	0.056±0.004	0.051±0.003	0.048±0.003	0.052±0.005	0.045±0.003	0.04±0.003
T_{13} **(7t/7h)**	0.172±0.004	0.146±0.003	0.129±0.004	0.114±0.003	0.098±0.004	0.091±0.004	0.075±0.003	0.065±0.003
T_{13} **(7t/8h)**	0.432±0.006	0.338±0.005	0.281±0.005	0.231±0.004	0.19±0.004	0.168±0.003	0.11±0.003	0.094±0.004
T_{13} **(7t/9h)**	0.272±0.008	0.208±0.006	0.167±0.005	0.133±0.004	0.112±0.005	0.091±0.005	0.066±0.003	0.052±0.003
T_{13} **(7t/10h)**	0.09±0.004	0.067±0.004	0.06±0.004	0.046±0.004	0.048±0.004	0.041±0.005	0.038±0.005	0.034±0.004
T_{13} **(8t/6h)**	0.054±0.004	0.047±0.003	0.053±0.003	0.047±0.003	0.049±0.004	0.045±0.003	0.038±0.004	0.04±0.003
T_{13} **(8t/7h)**	0.16±0.004	0.143±0.005	0.122±0.003	0.106±0.004	0.092±0.004	0.086±0.004	0.068±0.005	0.058±0.004
T_{13} **(8t/8h)**	0.194±0.004	0.15±0.004	0.119±0.004	0.102±0.004	0.086±0.004	0.072±0.004	0.061±0.004	0.052±0.003
T_{13} **(8t/9h)**	0.114±0.005	0.09±0.004	0.066±0.004	0.051±0.004	0.047±0.004	0.044±0.004	0.032±0.004	0.029±0.004
T_{13} **(8t/10h)**	0.043±0.004	0.037±0.003	0.037±0.003	0.035±0.004	0.03±0.004	0.027±0.005	0.028±0.004	0.036±0.005

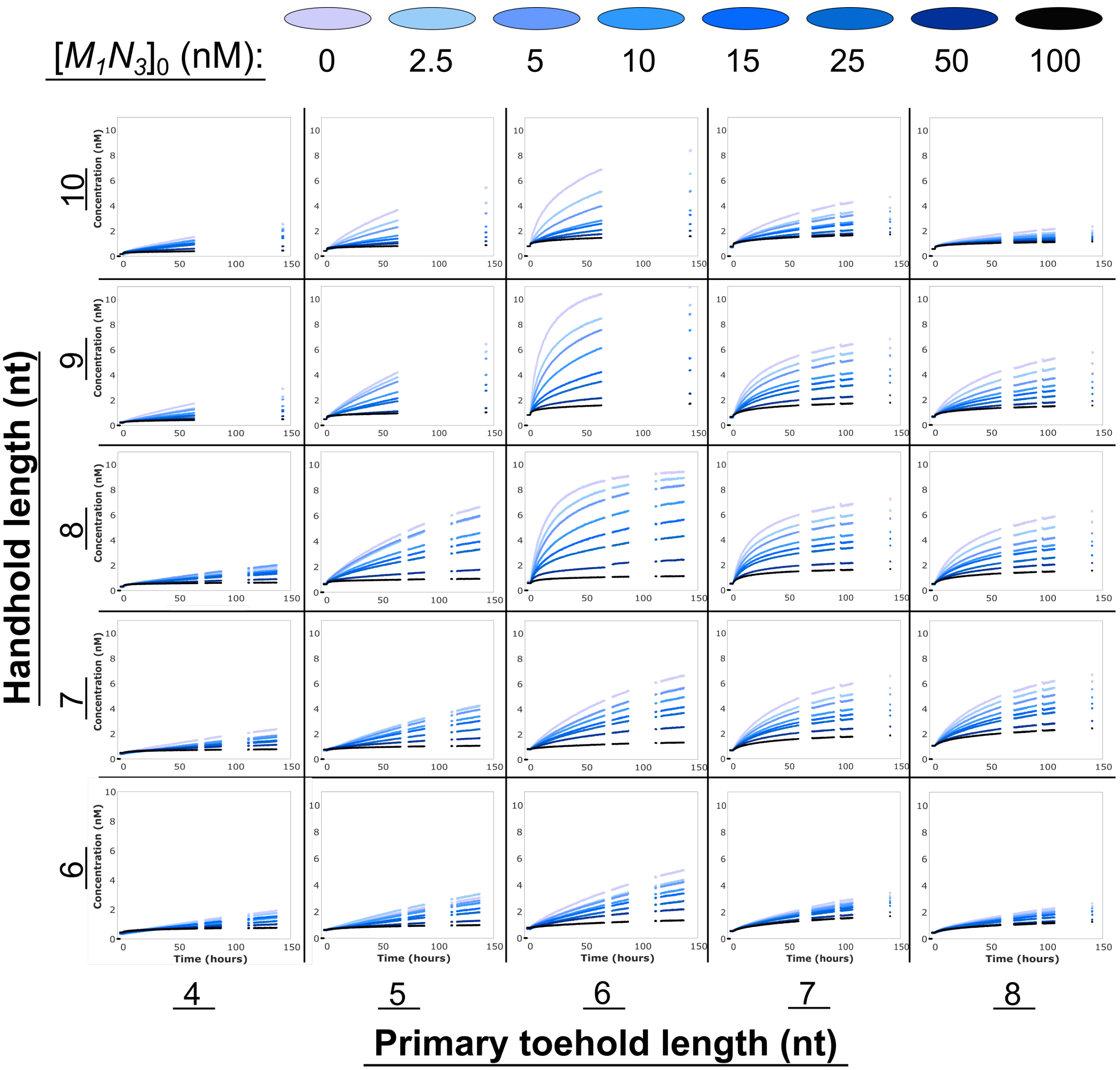

Supplementary Figure 15: Kinetics of 10 nm-scale inhibition tests. Reacted monomer concentration $[M_1L]$ in a system with 10 nM N_3, 10 nM M_1L, and 1 nM T_{13}, with variable lengths of t and h domains, and an initial non-fluorescent pool of products M_1N_3 at a range of concentrations $[M_1N_3]_0$.

7.2.1 Results for M_1L and N_3 dimerisation in the presence of M_1N_3, at high initial concentrations of substrate and product

To demonstrate the monomer-competitive nature of the observed product inhibition, inhibition testing was repeated, for different concentrations of T_{13} (6t/8h), and the monomers at an intended concentration of 100 nM. All fitted initial rates for inhibition experiments are collected in Supplementary Table 19. Complete trajectories are shown in Supplementary Figure 16. The obtained results demonstrate that, for the three $[T_{13}]_0$ tested (2.5, 5 and 10 nM), the addition of $[M_1N_3]_0$ produced a similar reduction in their TOF. Additionally, for this new concentration regime, the estimated $IC50$ for all template concentrations is $[M_1N_3]_0 \approx [M_1L]_0$.

Supplementary Table 19: Initial rates of monomer turnover by T_{13} in the presence of M_1N_3 (100 nM-scale). Data obtained using procedures described in Supplementary Table 18 and Supplementary Note 7. The reactant complexes used were M_1L (Supplementary Table 1) triggered by N_3 strands (Supplementary Table 3) in the presence of different concentrations of T_{13} (6t/8h) (Supplementary Table 1). Intended concentrations: $[M_1L]_0$ = 100 nM, $[N_3]_0$ 100 nM, $[T_{13}]_0$ = variable, $[M_1N_3]_0$ = variable. For easier comparison between different $[T_{13}]_0$ conditions, fitting results are reported as TOF, in hour^{-1}, with a 95% confidence interval.

**Template Concentration**	$[M_1N_3]_0$ **0 nM**	$[M_1N_3]_0$ **50 nM**	$[M_1N_3]_0$ **100 nM**	$[M_1N_3]_0$ **200 nM**	$[M_1N_3]_0$ **300 nM**
2.5 nM	4.5 ± 0.2	2.62 ± 0.12	1.42 ± 0.08	1.18 ± 0.07	0.94 ± 0.07
5 nM	4.5 ± 0.2	2.61 ± 0.09	2.07 ± 0.06	1 ± 0.03	0.77 ± 0.05
10 nM	3.4 ± 1.2	2 ± 1	1.3 ± 0.7	0.8 ± 0.6	0.7 ± 0.6

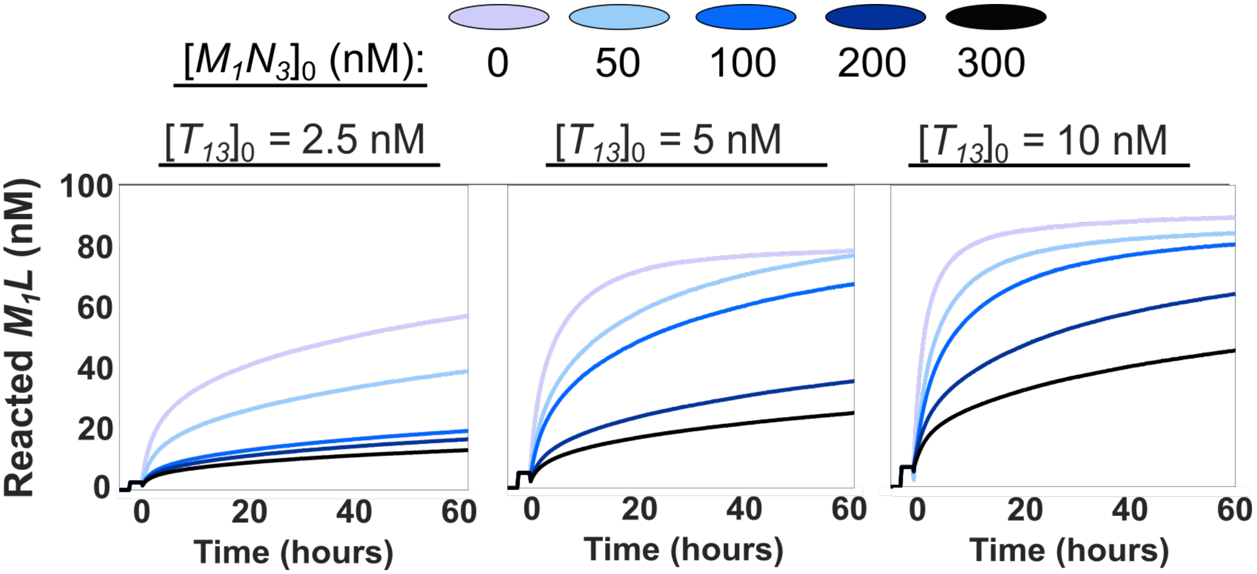

Supplementary Figure 16: Kinetics of 100 nM-scale inhibition tests. Reacted monomer concentration $[M_1L]$ in a system with 100 nM N_3, 100 nM M_1L, variable T_{13} (6t/8h), and an initial non-fluorescent pool of products M_1N_3 at a range of concentrations $[M_1N_3]_0$. Results demonstrate that the proportion of TOF reduction is largely determined by the ratio of monomers/products, demonstrating the competitive nature of the product inhibition in the system.

7.2.2 Characterisation of specific dimerization templates

All the different templates present in the selective dimerization assays were tested individually to confirm that they catalyzed formation of their intended products with similar rates between templates. Fitted initial rates for each template at different concentrations are collected in Supplementary Table 20. Complete trajectories are shown in Supplementary Figure 17. The initial rate of catalytic templating by T_{31} was obtained by fitting for 180 minutes instead of 30 as usual, due to the low signal-to-noise ratio of the condition. Supplementary Figure 18 collects the fittings demonstrating the linear relation of initial rate and template concentration for all the different tested templates. Obtained results correlate with the k_t values obtained individually for each M_xL complex, with T_{1y} having an overall higher TOF. All templates, except T_{22}, were capable of producing a discernible signal during the kinetics. The catalytic acticvity of T_{22} was alternatively confirmed through PAGE electrophoresis (Figure 4b).

Supplementary Table 20: Initial rates for monomer turnover by T_{xy} used during specific dimerization assays. Data obtained using procedures described in Supplementary Table 11 and Supplementary Note 7. The reactant complexes used were M_xL (Supplementary Table 11) triggered by N_y strands (Supplementary Table 3) in the presence of their complementary T_{xy} strand (Supplementary Table 1). Intended concentrations: $[M_1L]_0$ = 100 nM, $[N_3]_0$= 100 nM, and $[T_{13}]_0$ = variable. Fitting results are reported in nM hour^{-1}, with a 95% confidence interval. For each T_{xy} the TOF was estimated by a linear regression represented in Supplementary Figure 18. The results of two replicas were used to calculate the T_{13} TOF (Figure 3d).

**Template Condition**	$[T_{xy}]_0$ **5 nM**	$[T_{xy}]_0$ **3.75 nM**	$[T_{xy}]_0$ **2.5 nM**	$[T_{xy}]_0$ **1.25 nM**	$[T_{xy}]_0$ **0.5 nM**	$[T_{xy}]_0$ **0.25 nM**	**Fitted TOF (hour^{-1})**
T_{11}	11.5 ± 0.6	8.8 ± 0.5	5.6 ± 0.4	3.2 ± 0.2	1.3 ± 0.4	0.9 ± 0.2	2,24 ± 0.11
T_{12}	6.6 ± 0.6	5.1 ± 0.5	3.2 ± 0.2	2.3 ± 0.5	1.3 ± 0.3	1.01 ± 0.19	1.17 ± 0.11
T_{13}	19.7 ± 0.9	14.9 ± 0.6	9.9 ± 0.5	6.4 ± 0.3	2.79 ± 0.18	1.17 ± 0.08	3.6 ± 0.3
T_{21}	12 ± 3	9 ± 2	4 ± 3	3.4 ± 0.7	2.1 ± 0.7	1.7 ± 1.3	2.1 ± 0.5
T_{22}	0	0	0	0	0	0	
T_{23}	18 ± 2	12.1 ± 1.4	8 ± 1.5	3.8 ± 0.6	3.4 ± 1.5	3.3 ± 1.2	3 ± 1
T_{31}	3.9 ± 0.3	3.1 ± 0.4	1.42 ± 0.08	0	0	0	1 ± 1
T_{32}	6.3 ± 0.9	4.4 ± 1.2	4.5 ± 0.8	2.1 ± 1.4	1.9 ± 1.2	1.3 ± 1	1 ± 0.4
T_{33}	11.5 ± 1.3	10.9 ± 1.4	7.5 ± 0.8	4.3 ± 0.8	2.2 ± 0.4	2.2 ± 0.5	2.2 ± 0.5
**Template Condition**	$[T_{xy}]_0$ **5 nM**	$[T_{xy}]_0$ **2.5 nM**	$[T_{xy}]_0$ **1 nM**	$[T_{xy}]_0$ **0.5 nM**			**Fitted TOF (hour^{-1})**
T_{13} (replica)	18.6 ± 0.3	10.03 ± 0.14	3.85 ± 0.08	2.12 ± 0.06			Values considered for T_{13} TOF calculation above

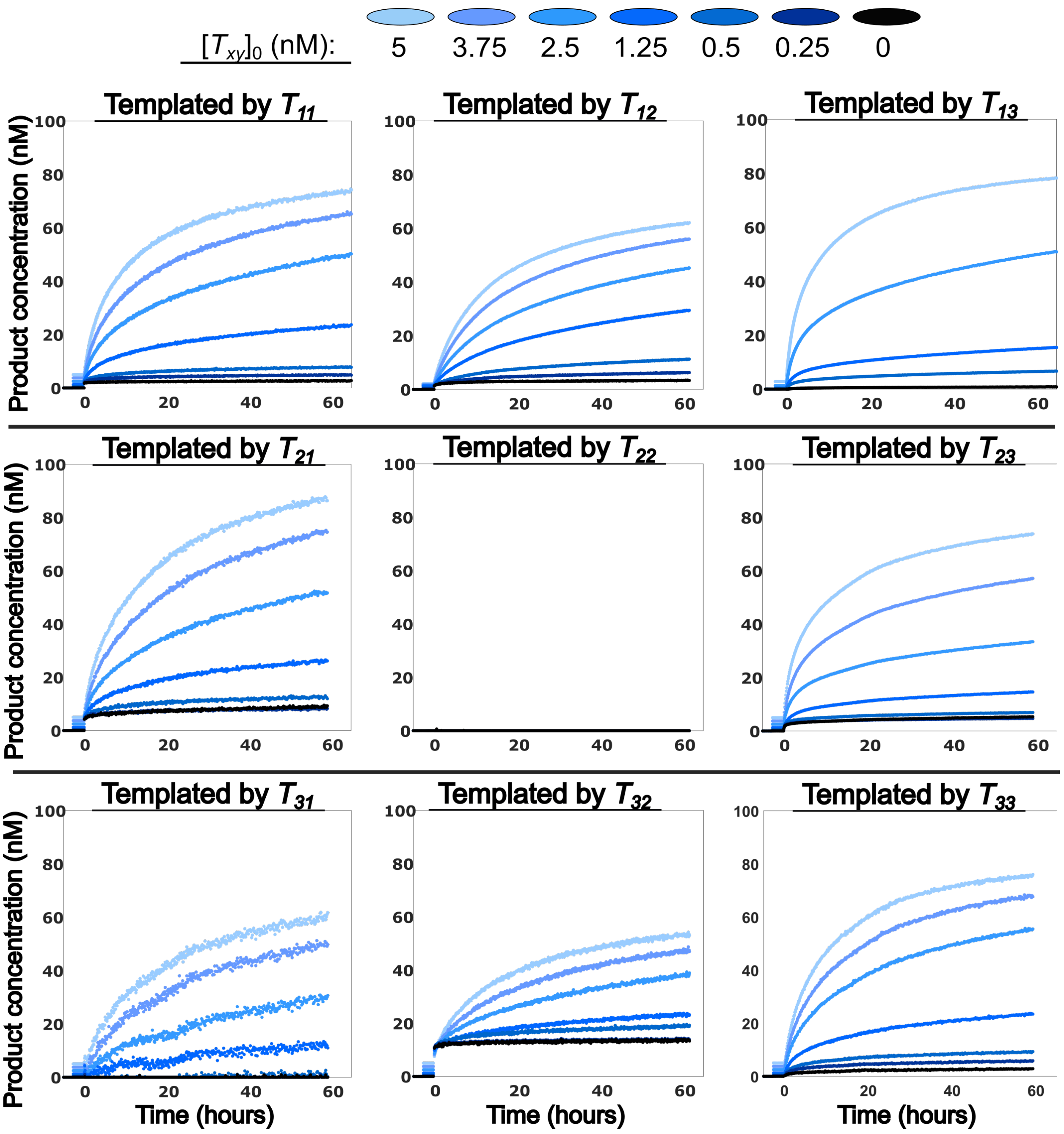

Supplementary Figure 17: Kinetics of the T_{xy}-mediated reaction in isolation. Formation of M_xN_y product in a system with 100 nM of a single N_y, 100 nM of a single M_xL, and variable $[T_{xy}]_0$ as indicated by the trace color (5 nM, 3.75 nM, 2.5nM, 1.25 nM, 0.5nM and 0.25 nM). The T_{13} panel contains only the kinetic traces not included in Figure 3c. The initial jump for conditions reading Green/Red FRET (T_{21} and T_{32}) may be the result of calibration errors for species in that fluorescence channel. T_{22} does not produe a detectable signal change upon dimerization.

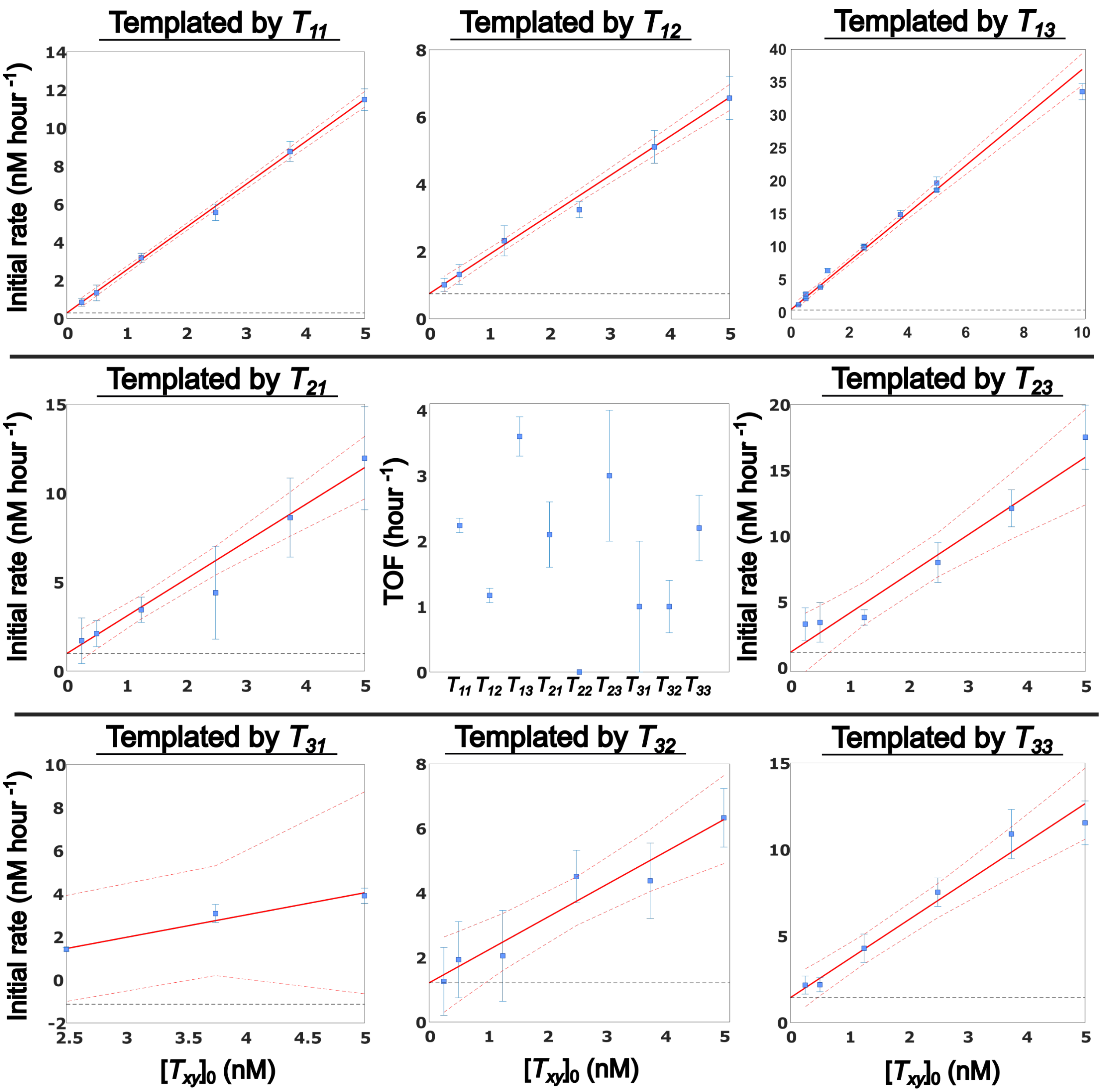

Supplementary Figure 18: Linear fits of sequence-specific dimerization templates' initial rates to obtain their TOF. Initial rates reported in Supplementary Table 20 were fitted to a linear model to calculate their TOFs. Exterior panels: Blue points show best fits to initial rates for single trajectories at each condition; error bars show a 95% confidence interval on that fit. The solid red line shows the best fit of a TOF through these independently estimated rates, with each data point inversely weighted by its confidence interval. The red dashed line gives that fit's 95% confidence interval, and the black dashed line the estimated initial rate in the absence of template. Central panel: Best fit TOFs (blue points) and 95% confidence intervals (error bars) for the systems shown in the exterior panels.

8 Supplementary Note 8: Putative design for longer templates

In Fig. 19, we illustrate how an HMSD-based system could – in principle – combat product inhibition for longer templates. Consider a template extended by adding more sites that look like the binding site of M_x in this work prior to a final truncated binding site analogous to the binding site for monomer N_y in this work. Monomers capable of recognizing these intermediate sites would have two polymerization domains, one in the forward direction and one in the backward direction, enabling them to form a long polymer.

In such a system, monomers binding to all but the final site of the template would bind stably until they have formed a polymerization bond in the forward's direction, just like M_x binds stably to the template until dimerized with N_y in the current work. Only the last monomer (the analog of N_y in this paper) would be truncated to allow spontaneous detachment of the whole product after it is incorporated. Thus, HMSD could ,in principle, be used to overcome the worst consequences of cooperative product inhibition whilst allowing partially-formed copies to remain stably attached to the template. Crucial to this functionality is the manner in which HMSD channels free energy from the dimerization reaction into destabilizing the binding of one of the target monomers to the template. Recent theoretical work has demonstrated that a system with these properties is, *a priori*, capable of producing copies of longer templates with surprising efficacy in wide regions of parameter space.[8]

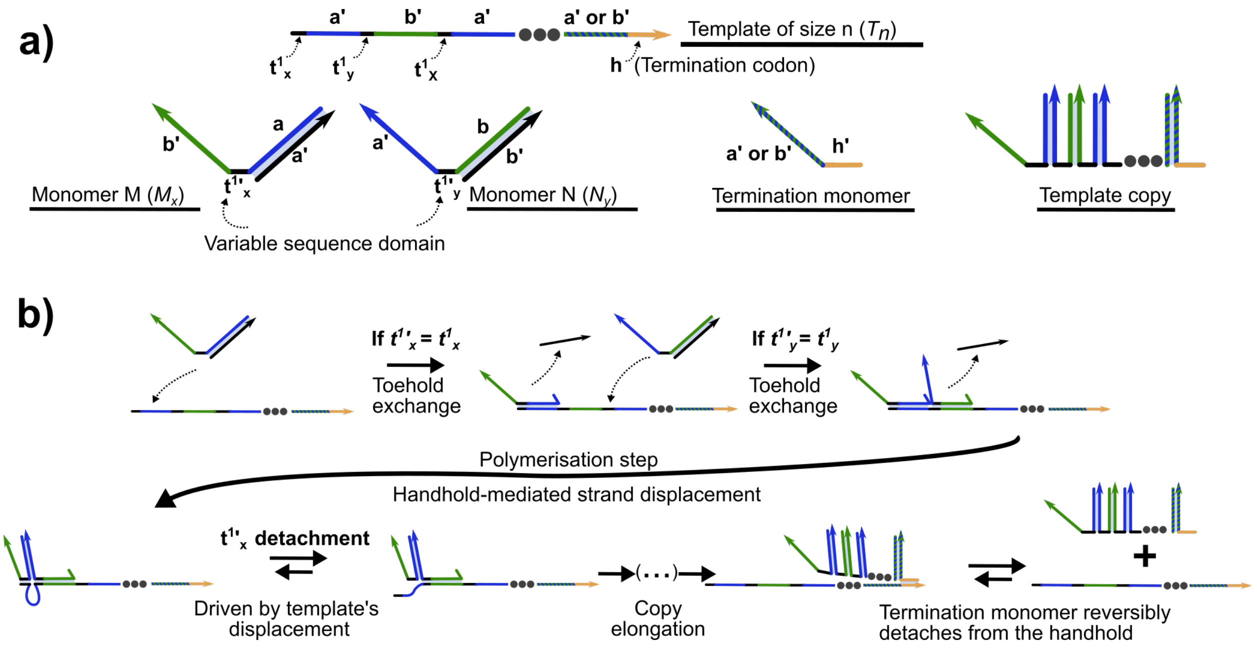

Supplementary Figure 19: Outline design of a system that exploits HMSD to copy a longer template. a) A long template consists of an alternating sequence of information-bearing toehold recognition domains and dimerization (or, in this case, polymerization) domains. Note that the dimerization domains themselves alternate (blue/green/blue...), to prevent self-interactions within the monomers. The final site of the template contains a truncated domain, which would play the role of a termination codon. The overall reaction would polymerize monomers of two different types (with interchanged polymerization domains) into a copy polymer with an information-bearing sequence of toehold recognition domains (shown in black here) and alternating polymerization duplexes. A truncated "termination" monomer caps the product. b) Detailed mechanism, showing iterated TMSD and HMSD reactions. Just as in the case of the dimer, HMSD would allow the product to bind only weakly to the template once the final termination monomer is incorporated. Note that, according to Juritz *et al.*,[8] such a system would actually operate best if multiple copies are bound to the template at the same time. We do not illustrate such a state for simplicity.

9 Supplementary Results 1: Results for alternate branch migration domain design

In this note we report a set of the experiments performed with a prototype of the dimerization mechanism, using the strands named 'v.1' (Supplementary Tables 1 to 3). All experiments described in this section followed the protocols described in Supplementary Note 4 for 'single fluorescence channel experiments'. Results were processed and fitted as described in Supplementary Sections 5.1 and 7.

The first tested version of the dimerization mechanism had strands with a branch migration domain of 28 nt and no clamps (Supplementary Figure 20a). Experiments with this version of the system proved successful as the dimerization reaction rate was completely dependent on template concentration for a variety of templates (results not included). The TOF of the template was also significantly higher than the final version of the system. In Supplementary Figure 20b we show one of the conditions tested for this prototype (T_{xy}(6t/8h)) which exhibits an initial TOF of 20 ± 4 hour^{-1}, calculated by fitting Supplementary Equation 25 to the first 10 minutes of the trajectories (Supplementary Figure 20c). This calculated initial TOF, for a monomer concentration of 60 nM, is faster than the 3.6± 0.3 hour^{-1} resulting from the final system with the same toehold and handhold domain lengths at a monomer concentration of 100 nM (a 5.6 ± 1.4-fold decrease) (Figure 3d). The difference in monomer concentrations mean that this ratio of TOF is a lower bound on the relative speed of the v.1 design. The higher TOF of the v.1 system is in turn explained by its higher k_t (k_t for M_1v.1 6t = 9.6 ± 0.6 x 10^5 M^{-1} s^{-1}, Supplementary Figure 21b).

However, this design was discarded as the monomers in solution suffered from a significant leak reaction, with a rate similar to that expected for a 1 nt toehold-mediated strand displacement (k_{leak} for M_1v.1 = 27.7 ± 0.2 M^{-1} s^{-1}, Supplementary Figure 21b). This leak reaction would have hindered the precision of the templated system in the specific dimerization regime, with a pool of six different monomers at high concentrations (Figure 5). The

final version of the system reduced k_{leak} for M_1L monomers to 0.29 ± 0.05 M^{-1} s^{-1} (a 96 ± 15-fold decrease, Supplementary Table 16). A sacrifice of reaction rate in exchange for leak suppression was considered a beneficial trade-off that significantly increased the precision of the specific dimerization templating.

We hypothesized that the relatively high k_{leak} arose from the partial complementarity of M_x's primary toehold domain with N_y's handhold. A leak reaction test with a handhold-less N_3 (N_3 v.1 (0h)) resulted, however, in only a reduction by half of k_{leak} (k_{leak} for strand Nv.1 (0h) = 11.4 ± 0.7 M^{-1} s^{-1}). That this k_{leak} was still high revealed that the main source of the leak was M_xL binding stability and a possible invasion from the fluorophore-labeled end of the complex. Aiming to reduce k_{leak}, the final version of the dimerization mechanism included 1 nt clamps at 3' and 5' of L strands. In addition, the branch migration domain length was increased to 41 nt to ensure binding stability between M_x and L, even in the presence of the two mismatches (Figure 1d).

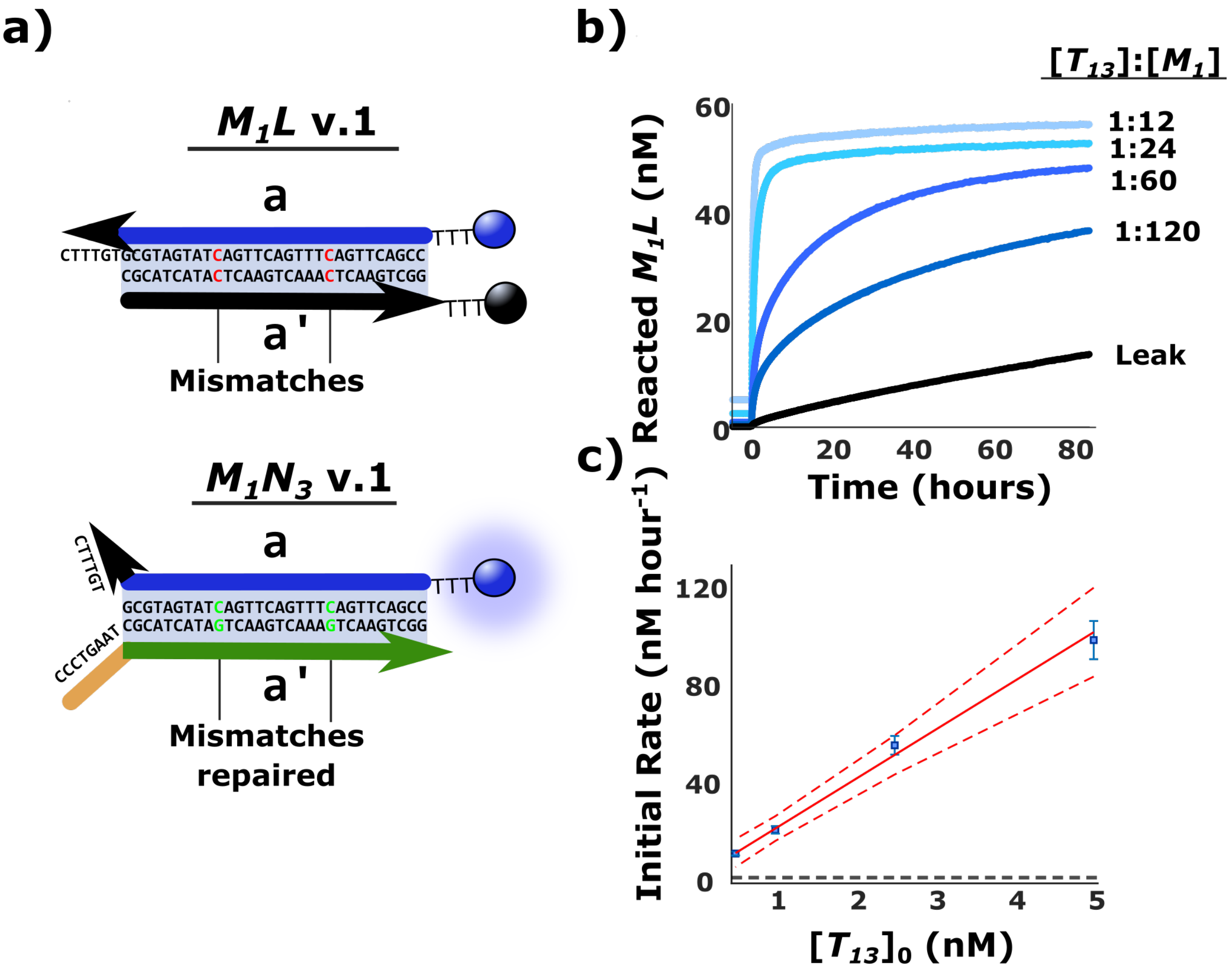

Supplementary Figure 20: Performance of the clamp-free version of the dimerization system. a) Detail of v.1 M_xL and M_xN_y complexes. These complexes had a shorter branch dimerization domain a of 24 nt and lacked 1 nt clamps in 3' and 5' extremes of the L strand. Two mismatched base pairs in M_xL's a were still included as a hidden thermodynamic drive, ensuring that dimerization is thermodynamically favored. b) Turnover of M_1L consumption as inferred from fluorescence data for v.1 strands. This simplified version of the dimerization system had a higher TOF but a high k_{leak}. Intended concentrations: $[M_1L \text{ v.1}]_0 = 60$ nM; $[N_3 \text{ v.1}]_0 = 66$ nM; $[T_{13} \text{ v.1}]_0 = [5, 2.5\ 1, 0.5$ and 0 nM$]$. c) TOF fitting from the initial rate of reaction from traces in the panel. Initial rates of reactions are plotted as a function of $[T_{13}]_0$. Blue symbols are centred on the best fit of the rate to a single trajectory, with error bars giving a 95% confidence interval on that fit. 'Red line': linear fit of the system TOF (3.6 $\pm$ 0.3 hour^{-1} to the 4 independent kinetic measurements; 'red dashed line': 95% confidence interval of that fit; 'black dashed line': untemplated rate for monomers at 100 nM = 0.012 $\pm$ 0.002 nM hour^{-1}.
Blue: experimental data; red dashed line: 95% confidence interval of the fit; red line: fit (TOF = 20 $\pm$ 4 hour^{-1}); black dashed line: estimated initial rate in the absence of template = 2 $\pm$ 2 nM hour^{-1}.

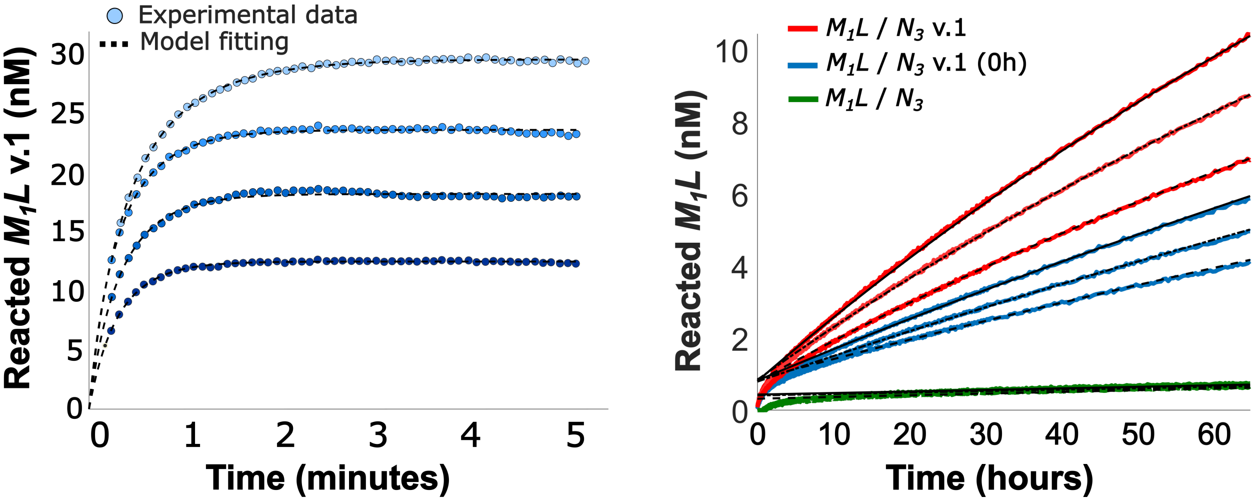

Supplementary Figure 21: Exploring reaction kinetics for the original version of the displacement domain. a) Monomer turnover inferred from fluorescence resulting from the binding reaction between M_1L v.1 complex and T_{13} v.1 (6t). The reaction proceeds with a higher rate (k_t = 9.6 ± 0.6 x 10^5 M^{-1} s^{-1}) than its final version counterpart (Supplementary Figure 11). Concentrations: $[M_1L \text{ v.1}]_0$ = 60 nM; $[T_{13} \text{ v.1}]_0$ = [30, 24, 18 and 12 nM]. b) Fluorescence resulting from the leak reaction between several designs of M_1L and N_3. The removal of handhold from N_3 v.1 resulted in a halving of k_{leak} (k_{leak} for M_1N_3v.1 = 27.7 ± 0.2 M^{-1} s^{-1}; k_{leak} for Nv.1 (0h) = 11.4 ± 0.7 M^{-1} s^{-1}). The addition of clamps and other design constraints in the DNA sequence of M_1L reduced k_{leak} by two orders of magnitude (M_1N_3). Conditions: The form of the model fitting line indicates $[N]_0$: – or 30 nM; -.- for 24 nM; and - - for 18 nM. For v.1 experiments: $[M_1L \text{ v.1}]_0$ = 60 nM. Conditions for final version: $[M_1L]_0$ = 100 nM.

10 Supplementary Results 2: Product yield during sequence-specific dimerization experiments

10.1 Iterations of the experiment

We performed three iterations of the sequence-specific dimerization, as described in Supplementary Table 14 with different proportions of M_xL, N_y and T_{xy} (results reported in Figures 4 and 5 and Supplementary Figures 22 to 28). Data was processed as described in Supplementary Section 5. For each iteration, the formation of the M_xN_y products was confirmed through PAGE. The results from the first and second iterations of the experiment, although demonstrating the specific formation of the template-specific products, exhibited appreciable formation of unintended products at long timescales for T_{13}. This formation of unintended products was apparently due to conversion of the M_1N_3 product onto alternatives M_1N_x. This interconvesion represents a slow relaxation towards equilibrium between the M_1-containing products.

We were able to largely eliminate this interconversion in iteration 3, after reducing both the concentration of T_{xy} and N_y monomers in the solution. In particular, we found that effective precision, given the possibility of interconversion, increased significantly when $M_x > N_y$. The nature of the mechanism for equilibrium relaxation of the products M_xN_y is further discussed in Supplementary Results 3.

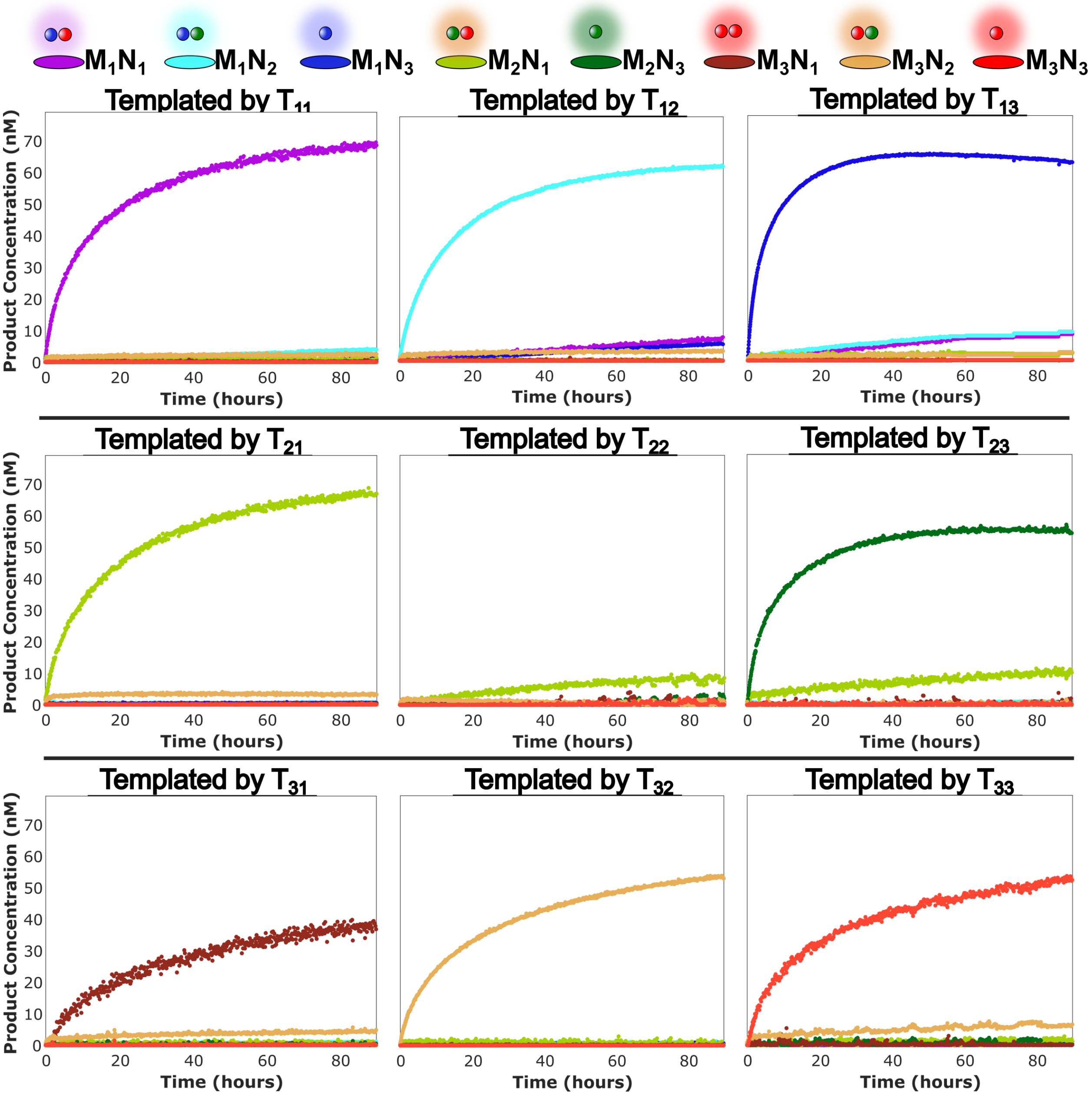

Supplementary Figure 22: Kinetics of first iteration of sequence-specific dimerization experiment. For each template, we report product concentrations for all species apart from M_2N_2, as inferred using the approach outlined in Section 5. This iteration showed moderate conversion of product M_1N_3 into M_1N1 and M_1N_2. Inferred concentrations of monomers (average of all wells): $[M_1L]_0 = 81$ nM; $[M_2L]_0 = 88$ nM; $[M_3L]_0 = 78$ nM; $[N_1]_0 = 93$ nM; $[N_2]_0 = 99$ nM; $[N_3]_0 = 96$ nM (average of $[N_1]_0$ and $[N_2]_0$). Intended $[T_{xy}]_0 = 5$ nM; intended $[M_3L]_0 = [N_y]_0 = 100$ nM

.

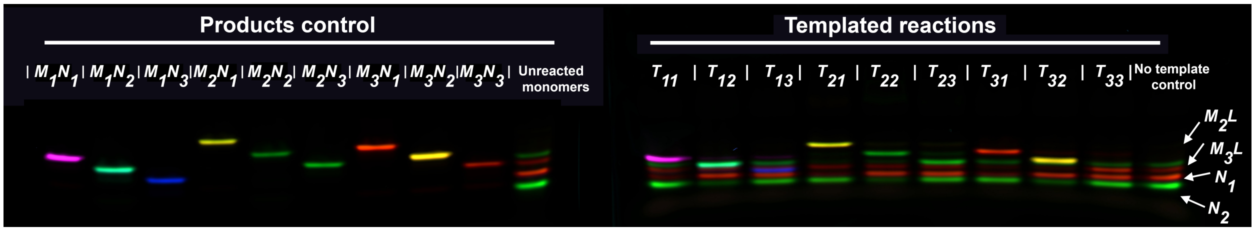

Supplementary Figure 23: PAGE of first iteration of sequence-specific dimerization experiment. Fluorescence emission gel electrophoresis of the products of the sequence-specific templating assay, in which a low intended concentration of a single T_{xy} (5 nM) is combined with an intended concentration of 100 nM of each M_xL monomer and 100 nM of each N_y monomer. The annealed controls have the same concentrations of M_x and N_y. The gel was run after a 90 hours of the experiment. Observed products are consistent with the intended M_xN_y, alongside bands corresponding to the unreacted monomers. False colors: blue, Alexa 488; green, Alexa 546; red, Alexa 647; cyan, FRET 488/546; yellow, FRET 546/648; purple, FRET 488/648)

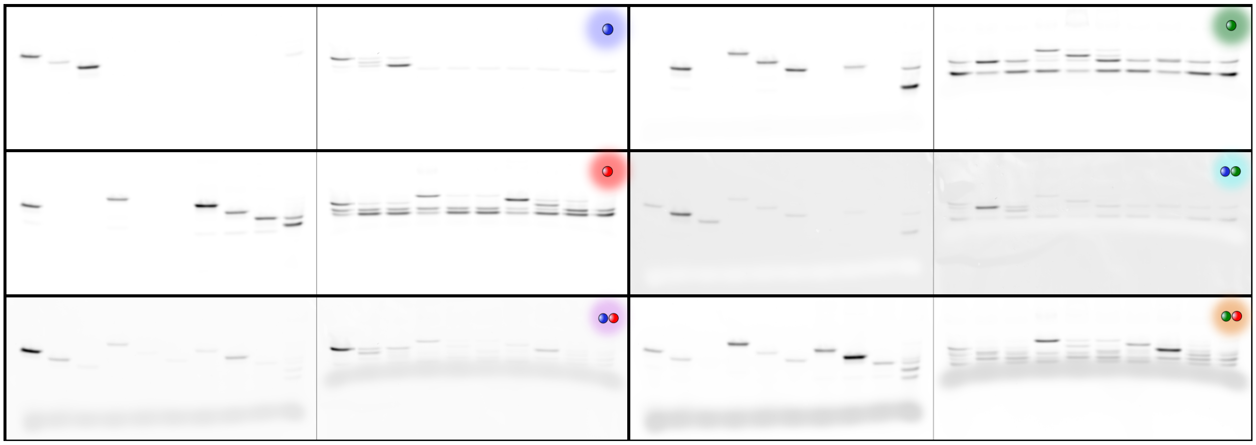

Supplementary Figure 24: Individual scans of the first iteration PAGE of sequence-specific dimerization experiment. Scans of the gel included in Supplementary Figure 23. Each channel shows in pairs the products control gel (left) and the templated reactions (right). For each gel, the six scans were merged to form the composite image. Legend: blue, Alexa 488; green, Alexa 546; red, Alexa 647; cyan, FRET 488/546; purple, FRET 488/648); ochre, FRET 546/648.

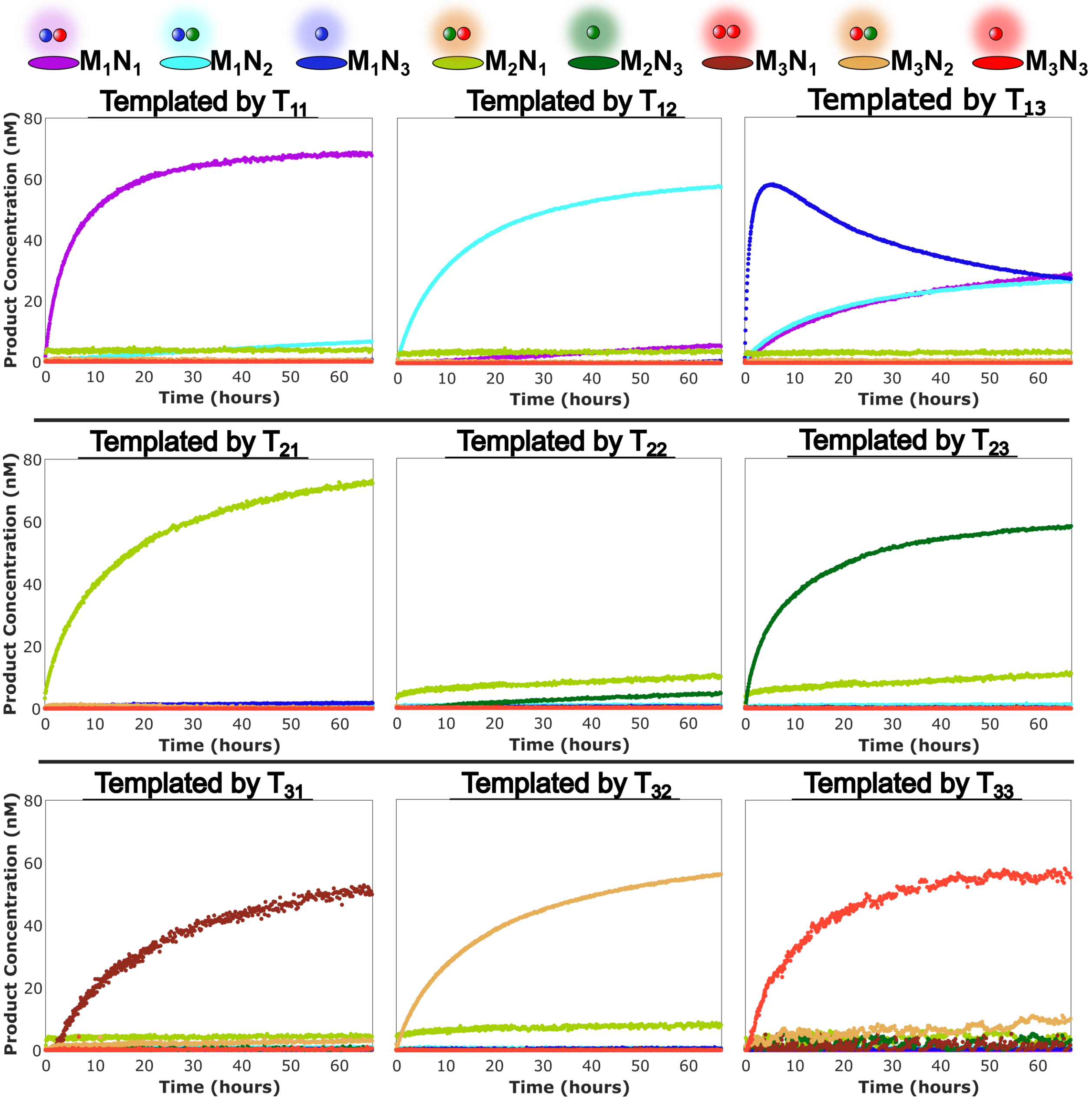

Supplementary Figure 25: Kinetics of second iteration of sequence-specific dimerization experiment. For each template, we report product concentrations for all species apart from M_2N_2, as inferred using the approach outlined in Section 5. This iteration, with a higher concentration of template, showed a clear tendency towards interconversion of the three M_1-containing products in the presence of T_{13}. Inferred concentrations of monomers (average of all wells): $[M_1L]_0 = 85$ nM; $[M_2L]_0 = 106$ nM; $[M_3L]_0 = 83$ nM; $[N_1]_0 = 95$ nM; $[N_2]_0 = 95$ nM; $[N_3]_0 = 95$ nM (average of $[N_1]_0$ and $[N_2]_0$); Intended $[T_{xy}]_0 = 10$ nM; intended $[M_3L]_0 = [N_y]_0 = 100$ nM

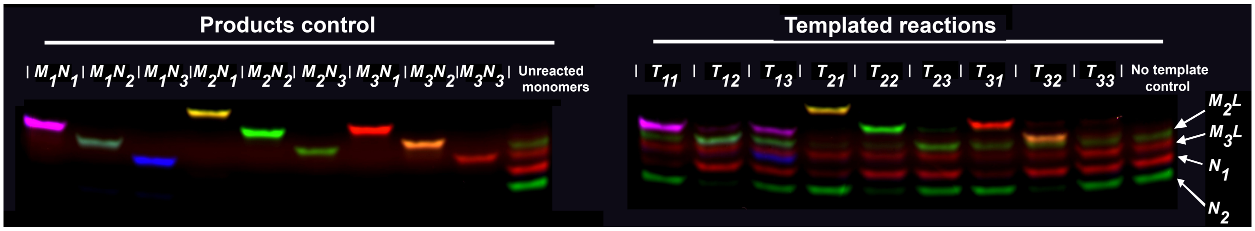

Supplementary Figure 26: PAGE of second iteration of sequence-specific dimerization experiment. Fluorescence emission gel electrophoresis of the products of the sequence-specific templating assay, in which a low concentration of a single T_{xy} (intended concentration 10 nM) is combined with an intended concentration of 100 nM of each M_xL monomer and 100 nM of each N_y monomer. Annealed control has the same concentrations of M_x and N_y. The gel was run after a 70 hours of the experiment. The condition templated by T_{13} produced similar concentrations of all M_1-containing products. Some formation of M_1N_2 is also detected for T_{11}, M_2N_2 for T_{23} and M_3N_2 for T_{33}. False colors: blue, Alexa 488; green, Alexa 546; red, Alexa 647; cyan, FRET 488/546; yellow, FRET 546/648; purple, FRET 488/648)

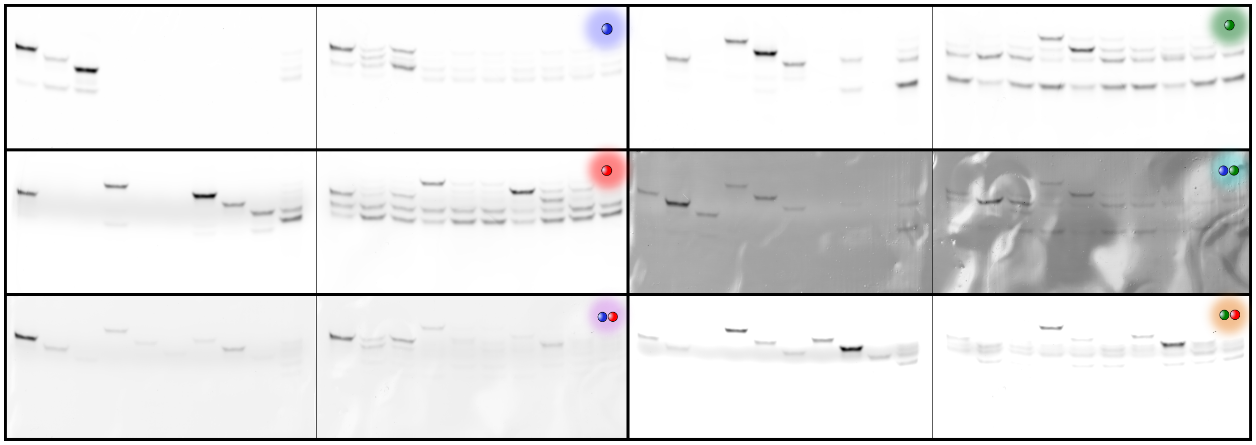

Supplementary Figure 27: Individual scans of the second iteration PAGE of sequence-specific dimerization experiment. Scans of the gels included in Supplementary Figure 26. Each channel shows in pairs the products control gel (left) and the templated reactions (right). For each gel, the six scans were merged to form the composite image. Legend: blue, Alexa 488; green, Alexa 546; red, Alexa 647; cyan, FRET 488/546; purple, FRET 488/648); ocre, FRET 546/648.

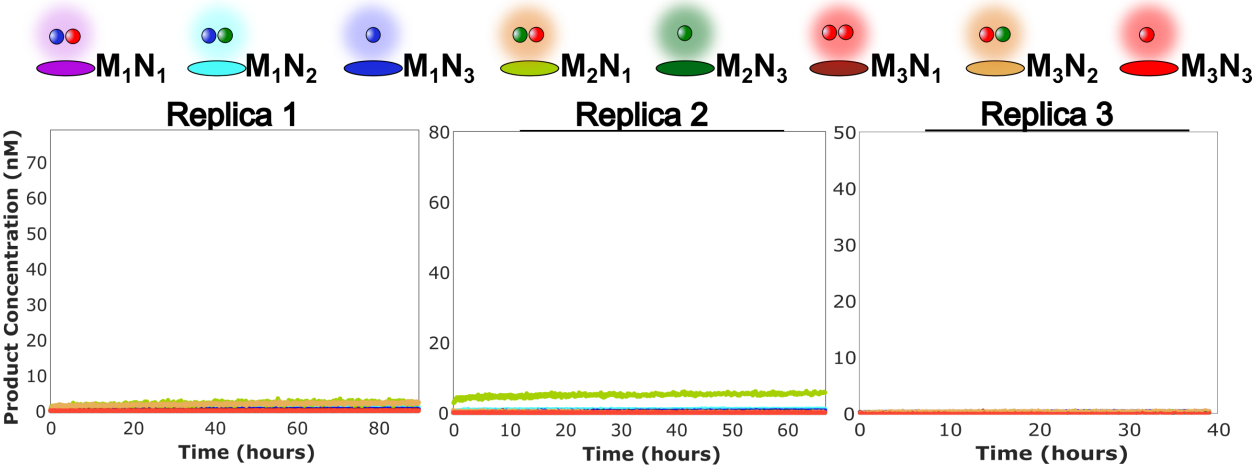

Supplementary Figure 28: Negative controls for specific dimerization experiments. Wells containing the same monomer concentrations as the experimental kinetics in their respective iteration in the absence of T_{xy}. The results demonstrate that the reaction of the monomers in the absence of T_{xy} is negligible. It also suggests that the M_2N_1 baseline in the second iteration may be due to a quantification error, rather than an avctual reaction.

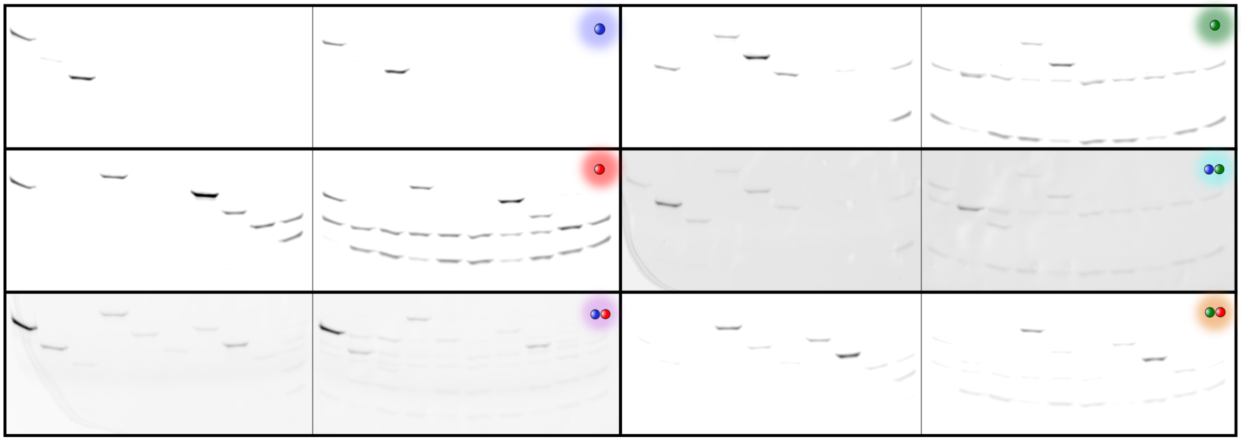

Supplementary Figure 29: Individual scans of the third iteration PAGE of sequence-specific dimerization experiment. Scans of the gels included in Figure 4 in the main text. These gels ran for slightly longer to remove the shadow of the loading dye. Each channel shows in pairs the products control gel (left) and the templated reactions (right). For each gel, the six scans were merged to form the composite image. Legend: blue, Alexa 488; green, Alexa 546; red, Alexa 647; cyan, FRET 488/546; purple, FRET 488/648); ochre, FRET 546/648.

10.2 Quantification of information propagation by templating

In order to estimate information propagation, we calculated the relative yield of each product in the three iterations of the sequence-specific copying experiments (Supplementary Figures 22, 25 and Figure 5 of the main text) after 24 hours of the experiment. Concentrations were estimated from the average of 8 timepoints, around 24 hours. Since the M_2N_2 signal could not be detected, for each template other than T_{2y}, $[M_2N_2]_{24h}$ was considered as the average of $[M_2N_1]_{24h}$ and $[M_2N_3]_{24h}$. For conditions using a T_{2y} template, $[M_2N_2]_{24h}$ was assumed to be equal to the production of the untemplated M_2N_y. In the case of T_{22}, $[M_2N_2]_{24h}$ was assumed to be the average of $[M_2N_1]_{24h}$ and $[M_2N_3]_{24h}$ when templated by their respective templates. We justify this approach by noting that the gels did not show M_2N_2 as behaving significantly differently from M_2N_1 or M_2N_3.

To interpret these yields in terms of information, we first calculate the probability of selecting each product dimer from the pool of dimers produced by a template, $P(M_xN_y|T_{vw})$, based on the relative concentrations. These conditional probabilities are reported in Supplementary Tables 21 to 23. We then calculate the mutual information between product and template (in bits) as

$$I = \sum_{xy,vw} \left(P(M_xN_y, T_{vw}) \log_2 \left[\frac{P(M_xN_y, T_{vw})}{P(M_xN_y)P(T_{vw})} \right] \right), \quad (29)$$

where $P(M_xN_y, T_{vw}) = P(T_{vw})P(M_xN_y|T_{vw})$, $P(M_xN_y) = \sum_{vw} P(M_xN_y, T_{vw})$ and assuming that templates are selected from a uniform distribution $P(T_{vw}) = 1/9$. Values for the mutual information are given in the captions to Supplementary Tables 21 to 23.

Supplementary Table 21: Templating precision of first specific-dimerization iteration. Product concentrations are expressed as the percentage of product per template, based on the experiment in Supplementary Figure 22. **Bold**: intended product for used template; *italics*: numbers relying on estimation of $[M_2N_2]_{24h}$. Information content: 2.31 bits out of a possible maximum of 3.17 bits.

	M_1N_1	M_1N_2	M_1N_3	M_2N_1	M_2N_2	M_2N_3	M_3N_1	M_3N_2	M_3N_3
T_{11}	**91.6**	2.67	0.06	1.12	*0.62*	0.11	0.2	3.47	0.14
T_{12}	3.54	**88.36**	2.51	0.24	*0.15*	0.05	0.13	4.96	0.06
T_{13}	4.58	5.92	**83.35**	2.35	*1.22*	0.09	0.08	2.39	0.01
T_{21}	0.02	0.78	0.55	**91.73**	*0.05*	0.05	0.21	6.51	0.1
T_{22}	*0.16*	*0.98*	*0.67*	*11.7*	***83.23***	*0.42*	*1.27*	*0.7*	*0.88*
T_{23}	0.01	0.73	0.32	9.57	*9.57*	**79.18**	0.22	0.24	0.16
T_{31}	0.29	2.16	1.09	2.8	*1.56*	0.32	**80.87**	10.58	0.33
T_{32}	0.34	1.43	0.93	2.89	*1.46*	0.04	0.23	**92.68**	0
T_{33}	0.06	1.5	0.65	1.99	*1.92*	1.86	1.04	9.55	**81.43**

Supplementary Table 22: Templating precision of second specific-dimerization iteration. Product concentrations are expressed as the percentage of product per template, based on the experiment in Supplementary Figure 25. **Bold**: intended product for used template; *italics*: numbers relying on estimation of $[M_2N_2]_{24h}$. Information content: 2.00 bits out of a possible maximum of 3.17 bits.

	M_1N_1	M_1N_2	M_1N_3	M_2N_1	M_2N_2	M_2N_3	M_3N_1	M_3N_2	M_3N_3
T_{11}	**86.65**	3.98	0.02	5.31	*2.75*	0.19	0.16	0.89	0.05
T_{12}	3.54	**85.29**	0.05	6.61	*3.4*	0.19	0.28	0.6	0.04
T_{13}	21.68	22.56	**48.93**	3.81	*1.93*	0.06	0.16	0.81	0.05
T_{21}	0.12	1.47	2.11	**94.42**	*0.18*	0.18	0.26	1.25	0.02
T_{22}	*0.11*	*1.4*	*0.54*	*12.49*	***82.05***	*3.2*	*0.15*	*0.04*	*0.02*
T_{23}	0.002	1.56	0.22	12.04	*12.04*	**73.84**	0.17	0.04	0.09
T_{31}	0	1.78	0.72	8.88	*4.82*	0.75	**77.76**	4.77	0.5
T_{32}	0.05	0.98	0.51	13.68	*6.89*	0.1	0.21	**77.52**	0.07
T_{33}	0	1.21	0.42	4.57	*4.72*	4.86	1.61	9.86	**72.76**

Supplementary Table 23: Templating precision of third specific-dimerization iteration. Product concentrations are expressed as the percentage of product per template, based on the experiment in Figure 5 of the main text. **Bold**: intended product for used template; *italics*: numbers relying on estimation of $[M_2N_2]_{24h}$. Information content: 2.46 bits out of a possible maximum of 3.17 bits.

	M_1N_1	M_1N_2	M_1N_3	M_2N_1	M_2N_2	M_2N_3	M_3N_1	M_3N_2	M_3N_3
T_{11}	**94.5**	1.64	0.03	0.36	*0.32*	0.27	0.32	2.4	0.16
T_{12}	4.29	**89.16**	0.03	0.91	*0.69*	0.47	0.45	3.92	0.08
T_{13}	4.66	3.69	**87.49**	0.48	*0.26*	0.04	0.22	3.07	0.08
T_{21}	0.41	0.75	1.03	**94.12**	*0.1*	0.1	0.11	3.31	0.07
T_{22}	*0.68*	*0.34*	*1.28*	*8.6*	***87.45***	*0.19*	*0.25*	*1.14*	*0.06*
T_{23}	0.37	0.33	1.27	9.46	*9.46*	**78.54**	0.4	0.05	0.12
T_{31}	0.33	0.62	1.67	0.25	*0.22*	0.19	**86.94**	8.68	1.11
T_{32}	0.71	0.87	1.54	2.79	*1.45*	0.11	0.37	**92.14**	0.02
T_{33}	0.32	0.3	0.8	0.11	*0.14*	0.17	0.96	9.8	**87.39**

11 Supplementary Results 3: Mechanism for product interconversion

To explain the conversion of M_{13} into M_{12} and M_{11} observed in Supplementary Figures 22 and 25, we hypothesized that T_{xy}, as well as templating the formation of M_xN_y, can also increase the rate of relaxation of the dimer distribution towards equilibrium by favoring the interconversion between products that share the same M_x.

It is possible for product M_xN_y to rebind to T_{xy}, in the process releasing N_y (Supplementary Figure 30a,c). Particularly for reaction regimes where non-template-complementary $N_{y'}$ are at a higher concentration than the template-complementary N_y, e.g, after several days of reaction, $T_{xy}M_x$ can then react through a slow TMSD with non-templated $N_{y'}$ (Supplementary Figure 30c). This process would result in the T_{xy}-mediated destruction of the initially templated product, and its conversion into $M_xN_{y'}$. The addition of an excess of M_xL over N_y can prevent this destruction mechanism by having the templates preferentially blocked by occupied by M_x when the supply of new monomers runs out. Since there is no intended thermodynamic bias towards one product or another, regardless of which template is present, we would expect all systems to exhibit this relaxation towards equilibrium on sufficiently long-time scales.

We now explore this hypothesis for the cause of product interconversion.

11.1 t-complementarity between T_{xy} and M_xN_y is necessary for product destruction

We tested the kinetics of product destruction in several experiments where an initial concentration of M_1N_3 (Measurement 1) is mixed with T_{xy} (Measurement 2) and N_1 (Measurement 3 - kinetics). Product concentrations were inferred using fluorescence matrix 7 and processed as described in Supplementary Note 5.1. Supplementary Figure 30b shows the results of a set of experiments with intended concentrations of $[M_1N_3]_0 = 50$ nM, $[N_1]_0 = 150$ nM and

different T_{xy} with variable concentrations. The results demonstrate both that interconversion requires a template and that this interconversion is T_{xy}-dependent. In particular, it mainly relies on complementarity with the primary toehold.

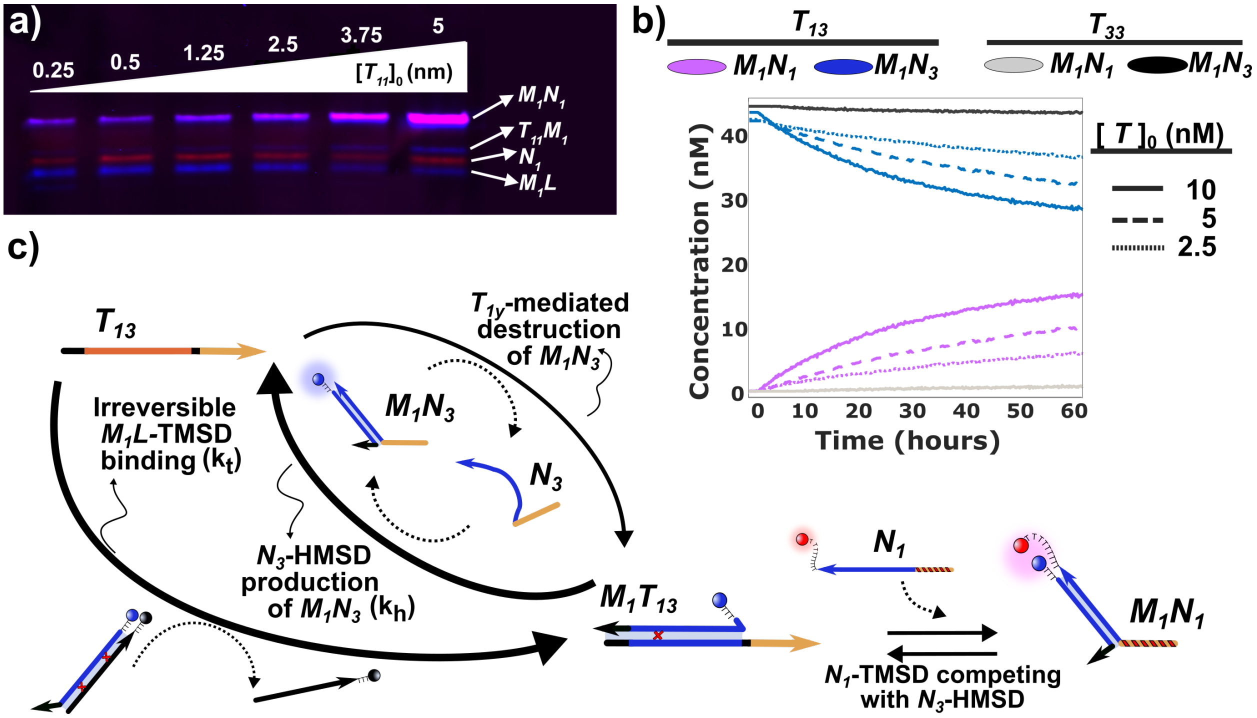

Supplementary Figure 30: Template-mediated interconversion of products. a) PAGE of the catalytic production of M_1N_1 after over 100 hours of reaction (T_{11} kinetics as shown in Supplementary Figure 17). Even after several days of reaction, the consumption of monomers was not complete. However, the gel demonstrates that the inhibition mechanism is not just the binding of M_xN_y to T_{xy}. Rather than being inhibited by the expected three-stranded complex (M_1N_1/T_{11}), T_{11} only had M_1 attached, hinting at the ability of T_{11} to break apart, rather than just bind to, M_1N_1. Intended concentrations: $[M_1 - L]_0 =$ 100 nM, $[N_1]_0 = 100$ nM, $[T_{11}]_0 = [0.25, 5]$ nM. b) The template-mediated destruction of M_xN_y needs a t-complementary T_{xy}. A mixture of M_1N_3 and N_1 slowly interconverts into M_1N_1 in the presence of the product-complementary template T_{13}. The rate of interconversion is proportional to the amount of template in the mixture. However, removing t complementarity (T_{33}) makes the template inert; interconversion is slow, without observable differences in the rate of interconversion for 2.5 or 10 nM of the template. Intended concentrations: $[M_1N_3]_0 = 50$ nM, $[N_1]_0 = 150$, $T_{xy} =$ variable. c) Proposed mechanism for M_1N_3 destruction given the experimental evidence. During the dimerization reaction, the produced M_1N_3 is in competition with M_1L to reattach to the template. This reattachment is mediated by a t-dependent TMSD. An excess of M_1L over the product would prevent the binding of M_1N_3 back to the template. While the template is occupied ($T_{13}M_1$), unspecific dimers can form slowly through a secondary toehold-mediated TMSD. The formation of unspecific products through this method becomes relatively more prevalent as the handhold-complementary N_y gets consumed.

11.2 Secondary toehold-mediated TMSD in the absence of handhold-complementarity is M_xN_y-destruction limiting factor

To further understand the dynamics of non-templated M_xN_y formation we tested the rate of reaction between the $T_{xy}M_x$ complex and non-handhold complementary N_y (with rate constant k_{t2}). An initial concentration of M_xL (Measurement 1) is mixed with T_{xy} (Measurement 2) and a non-handhold complementary N_y (Measurement 3 - kinetics). Product concentrations were inferred as described in Supplementary Section 5.1. An approximate value of k_{t2} was estimated by assuming that the complex $T_{xy}M_x$ is in a steady state during the linear regime of the kinetics fitted (Supplementary Figure 31):

$$k_{t2} = \frac{1}{[N_y]_0[T_{xy}]_0}\left(\frac{1}{\frac{d[M_xN_y]}{dt}} - \frac{1}{[T_{xy}]_0[M_x]_0k_t}\right), \tag{30}$$

with k_{t2} being the rate of the secondary toehold-mediated TMSD; $\frac{d[M_xN_y]}{dt}$ the slope of the linear fit to the kinetics; $[X]_0$ the intended (or estimated in the case of Supplementary Figure 31b) initial concentrations of the reagents; and k_t assumed to be the average k_t for the M_xL used (Supplementary Note 7). The obtained results demonstrate that the TMSD reaction mediated by the secondary toehold is over three orders of magnitude slower than the templated HMSD of the correct monomer. For $T_{x2}M_x$ reacting with N_3, k_{t2} with $M_1 = 1.21 \pm 0.06$ M^{-1} s^{-1}, with $M_2 = 8.78 \pm 0.04$ M^{-1} s^{-1} and with $M_3 = 2.12 \pm 0.02$ M^{-1} s^{-1}(Supplementary Figure 31a). For $T_{12}M_1$ reacting with N_1, $k_{t2} = 53$ M^{-1} s^{-1} (Supplementary Figure 31b). We note in passing that the faster invasion of N_1 and N_2 than N_3 is consistent with interactions between the fluorophore labels – absent in N_3 – stabilizing both the products M_xN_y and enhancing the toehold-mediated reaction.

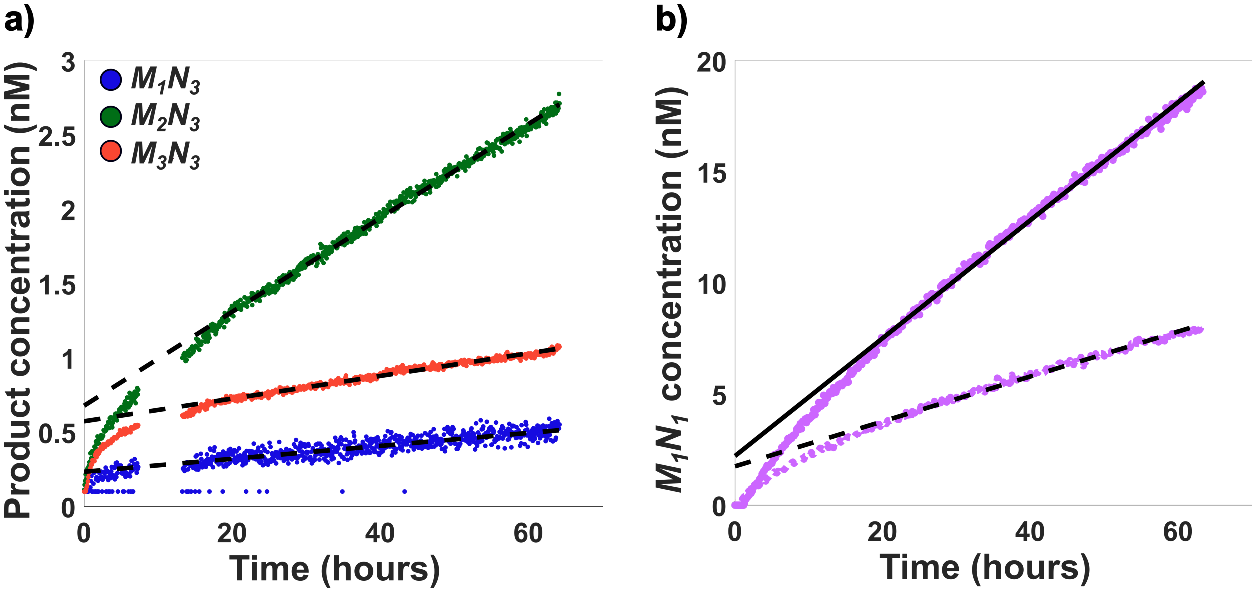

Supplementary Figure 31: Secondary toehold mediated TMSD reaction without handhold complementarity. a) TMSD reaction of $T_{x2}M_x$ with N_3 in the absence of handhold complementarity. In the absence of handhold interaction, the 2 nt secondary toehold can still mediate a slow displacement reaction. Intended concentrations: $M_{1-3}L = 100$ nM; $N_3 = 100$ nM, $T_{x2} = 10$ nM. b) TMSD reaction of $T_{12}M_1$ with N_1 in the absence of handhold complementarity. The presence of a fluorophore nearby the secondary toehold domain of N_y increases the rate of untemplated TMSD by one order of magnitude relative to N_3. This fluorophore-induced slightly increased stability explains the prevalence of double-labeled incorrect products. Intended concentrations: $T_{12} = 10$ nM, $M_1L = 150$ nM and $N_1 = 100$ nM (solid fit line) or 50 nM (dashed fit line).

12 Supplementary Results 4: Displacement of $\boldsymbol{L}$ from $\boldsymbol{M_1L}$ by $\boldsymbol{T_{13}}$ is effectively irreversible

To confirm whether M-type monomers bind irreversibly to the template, we ran the dimerization reaction (Supplementary Table 11) with varying amounts of additional lock (Supplementary Figure 32). Experiments consisted of a solution with intended concentrations of $[M_1L]_0 = 50$ nM, $[N_3]_0 = 40$ nM, $[T_{13}\ (6t/8h)]_0 = 10\ \text{nmol dm}^{-3}$ and a variable $[L]_0$ of either 60, 120, 300 or 1000 nM. The control demonstrates that the accumulation of L strand does not produce significant differences between the traces, even for a ratio $[L]_0$:$[T_{xy}]_0 = 100$. The results confirms that L is effectively inert after it has been displaced. Is therefore concluded that premature release of M_1 from T_{xy} by L is negligible.

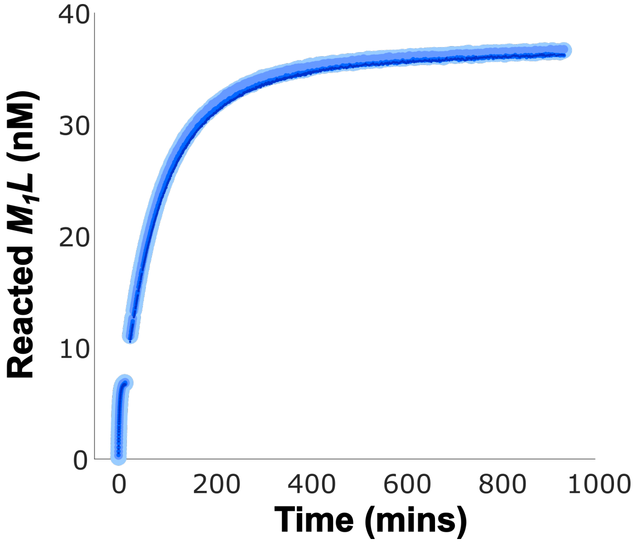

Supplementary Figure 32: Evidence of the effective irreversibility of the displacement of $\boldsymbol{L}$ from $\boldsymbol{M_1L}$ by $\boldsymbol{T_{13}}$. Displacement of L from M_1L by T_{13}, carried out with background lock concentrations of 60, 120, 300 or 1000 nM. According to the experiment, the lock strand is effectively inert, given that the four replicas exhibit the same kinetics during both sets of kinetics recorded: 1. the initial displacement of M_1L by T_{13}, and 2. the addition of N_3 to start the catalytic dimerization. To help identify the four near-identical traces, increasing $[L]_0$ conditions are represented with gradually smaller and darker data points. Intended initial conditions: $[M_1L]_0 = 50$ nM, $[T_{13}\ (6t/8h)]_0 = 10\ \text{nmol dm}^{-3}$, $[N_3]_0 = 40$ nM.

13 Supplementary Results 5: Identification of bands in the trimerization experiment

In Supplementary Figure 33 and Supplementary Figure 34, we propose identities for the most visible bands in the gels reported in Figure 6b of the main text. These figures show the same gels as in 6b, but include separate (false colour) images for red, green and blue channels alongside the composite, and we have enhanced the contrast to allow even fairly faint fluorescence to be detected, at the cost of increasing background. Note that the adjustment of the brightness, which is different in each image, means that the apparent colour in the composite is somewhat different from the main text.

We deduce the identity of the bands by comparing them to controls in Supplementary Figure 35, as well as the intended trimer controls in 33. We base this comparison on migration speed relative to easy to identify bands such as the monomers and $A_1B_1C_1$, $A_2B_2C_2$, $A_1B_1L_B$ and $A_2B_2L_B$, and which combination of red, blue and green fluorescence are visible in the band (A_1, B_1, and C_1 have red, green, and blue fluorophores, respectively; A_2, B_2, and C_2 have blue, red, and green fluorophores, respectively). Note that the brightness of these fluorophores changes according to the complex of which they are a part, due to FRET; we do not attempt to use this additional information. The known controls were prepared by annealing the strands in a thermocycler, and then subjected to PAGE and visualized in the gel scanner. We confirmed that the dominant bands had fluorescence in the expected channels; these channels are identified via "RGB" annotations in Supplementary Figure 35. Note that, in order to identify complexes of products and templates, these controls were performed at a much higher relative concentration of templates than the original experiments.

In general, identification of the strongest bands is reasonably straightforward. Note that in some cases the colour in the composite image looks somewhat different between controls and trimerization data, due to the contrast used. For example, the band corresponding to complex B_2C_2 in Figure 6b of the main text appears red when only the second set

of monomers and $T_{BC_{22}}$ are present, and orange in the experiments where both sets of monomers and second set of templates were present, whereas it is yellow in the controls (Supplementary Figure 35). This difference reflects the fact that the contrast of the green pseudo-colour was reduced in Figure 6b of the main text, and particularly for the gels of the experiments with separate monomer types, because of the presence of a green smear on the imaging stage. Nonetheless, the presence of both green and red fluorescence in this band is evident in Supplementary Figure 33.

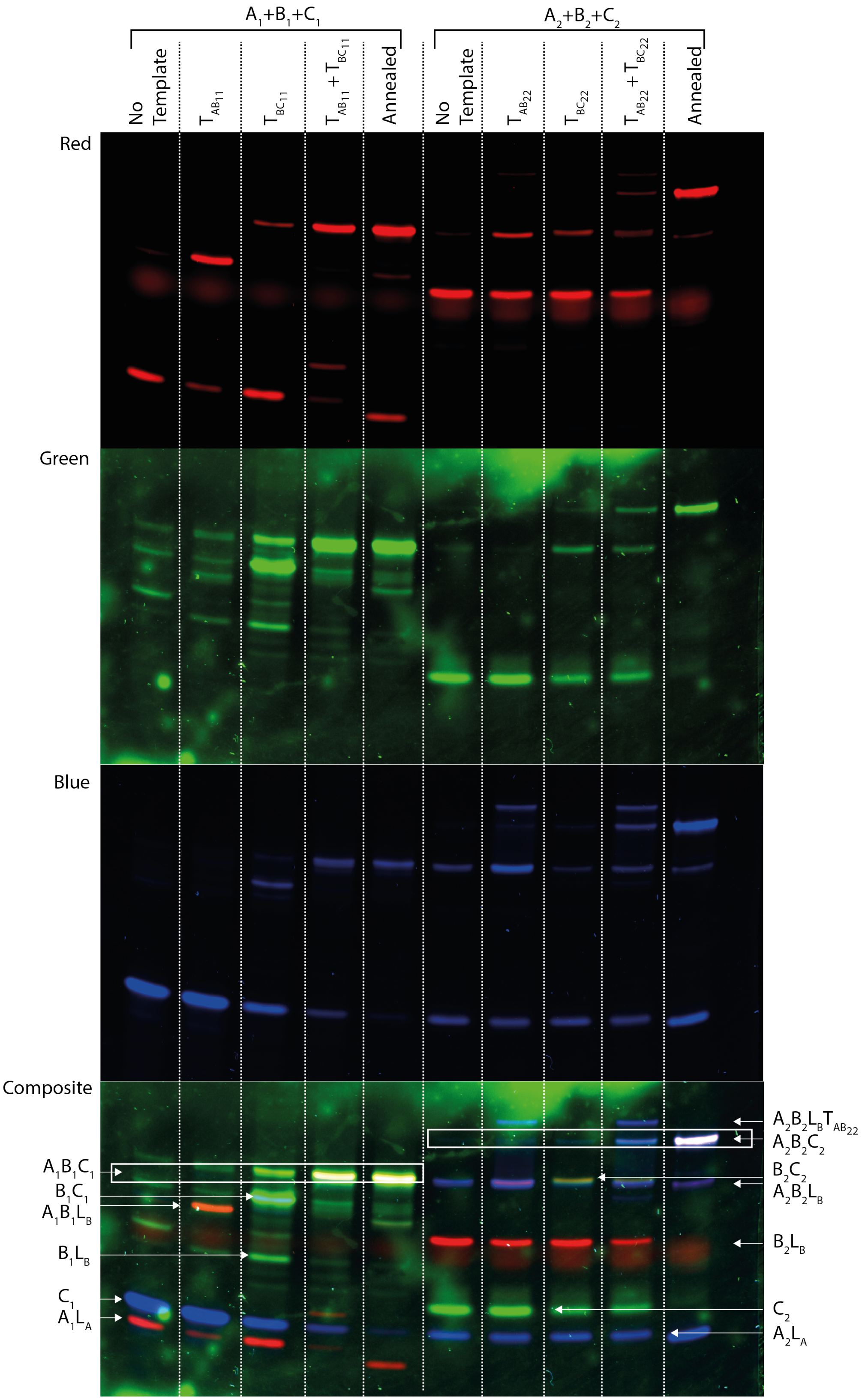

Supplementary Figure 33: Fluorescence image of the polyacrylamide gel of Figure 6b for the separate experiments on (A_1, B_1, C_1) and (A_2, B_2, C_2), visualising the separate fluorescence channels and a composite image with labelled bands. Blue: 488 nm laser excitation, 525BP20 emission filter, Green: 532 nm laser excitation, 570BP20 emission filter, Red: 635 nm laser excitation, 670BP20 emission filter.

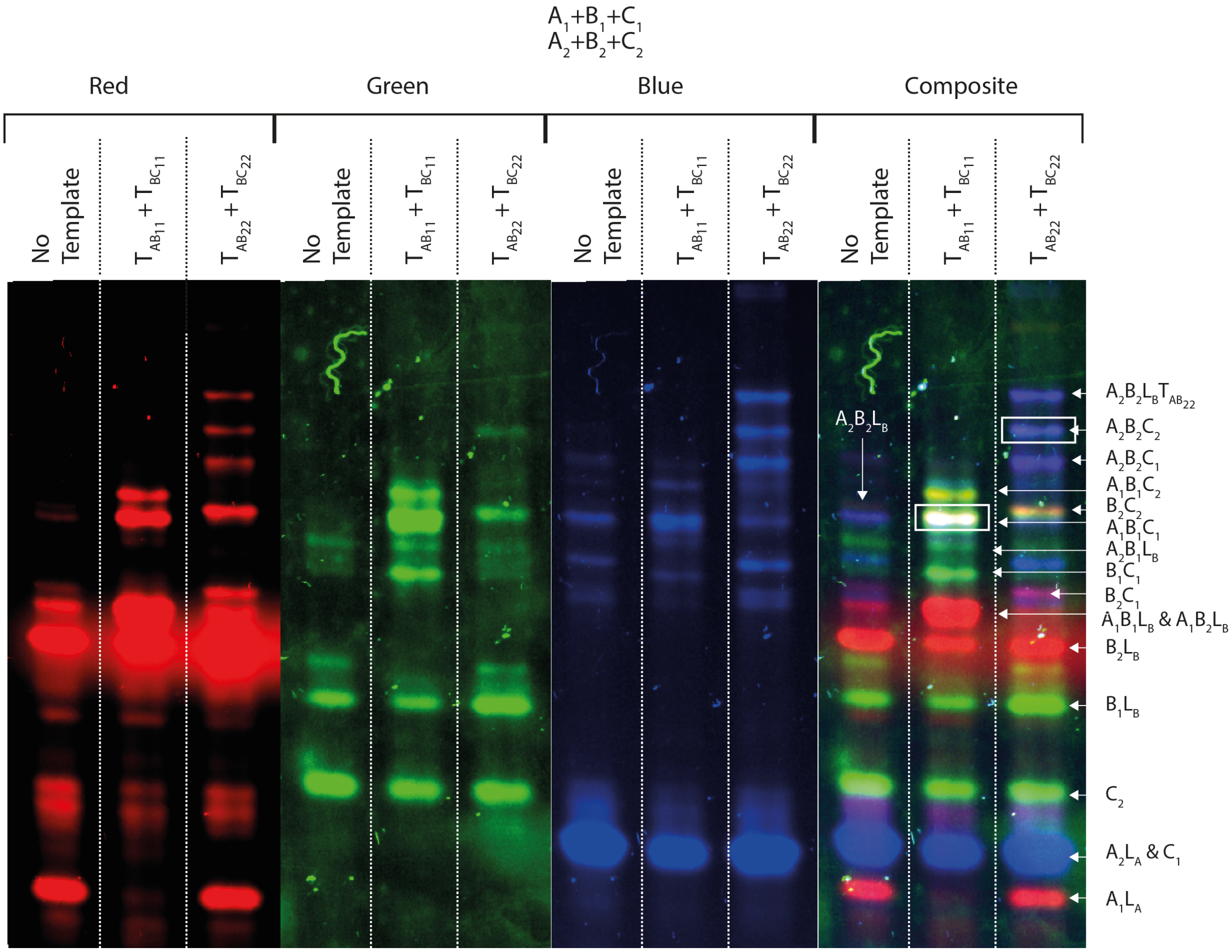

Supplementary Figure 34: Fluorescence image of the polyacrylamide gel of Figure 6b for the experiments on combined monomer pools ($A_1, B_1, C_1, A_2, B_2, C_2$), visualising the separate fluorescence channels and a composite image with labelled bands. Blue: 488 nm laser excitation, 525BP20 emission filter, Green: 532 nm laser excitation, 570BP20 emission filter, Red: 635 nm laser excitation, 670BP20 emission filter.

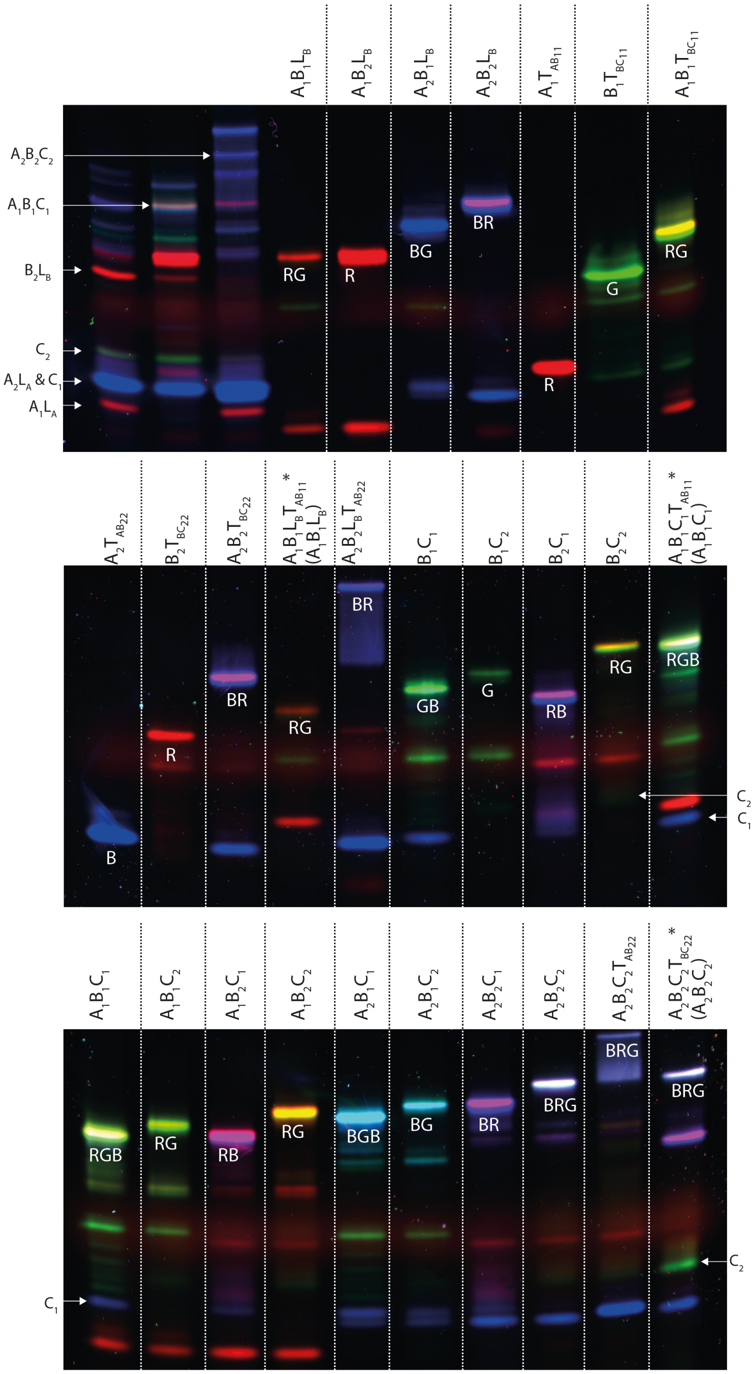

Supplementary Figure 35: Composite fluorescence image of gel electrophoresis of several control complexes including possible permutations of trimers, dimers, and other template-bound intermediates. The intended pre-annealed complexes are mentioned above their corresponding lanes on the gel images. Three of the lanes have "Intended complex* (actual complex)" notation, where the tubes were annealed with the strands to assemble the "intended complex*" but due to their unstable nature, "(actual complex)" was obtained. The intended complex bands, except in three aforementioned lanes, are annotated underneath to indicate which fluorescence channels they appeared in, where B: 488 nm laser excitation, 525BP20 emission filter, G: 532 nm laser excitation, 570BP20 emission filter, R: 635 nm laser excitation, 670BP20 emission filter. In those three "Intended complex* (actual complex)" lanes, the "(actual complex)" bands are annotated. For comparison, the top image also contains a trimerisation experiment, with the visible monomer bands and the intended products $A_1B_1C_1$ and $A_2B_2C_2$, which are easy to identify by comparison with Figure 6 of the main text, labelled.

References

(1) Soloveichik, D.; Seelig, G.; Winfree, E. DNA as a universal substrate for chemical kinetics. *Proc. Nat. Acad Sci. USA* **2010**, *107*, 5393–5398.

(2) Chen, Y.-J.; Dalchau, N.; Srinivas, N.; Phillips, A.; Cardelli, L.; Soloveichik, D.; Seelig, G. Programmable chemical controllers made from DNA. *Nat. Nanotechnol.* **2013**, *8*, 755–762.

(3) Lankinen, A.; Mullor Ruiz, I.; Ouldridge, T. E. Implementing Non-Equilibrium Networks with Active Circuits of Duplex Catalysts. 26th International Conference on DNA Computing and Molecular Programming (DNA 26). Dagstuhl, Germany, 2020; pp 7:1–7:25.

(4) SP&A Application Laboratory, T. F. S. Microplate-Based Pathlength Technical Notes Correction Method for Photometric DNA Quantification. 2015.

(5) The MathWorks, I. Curve Fitting Toolbox. 2020.

(6) Zhang, D. Y.; Winfree, E. Control of DNA strand displacement kinetics using toehold exchange. *J. Am. Chem. Soc.* **2009**, *131*, 17303–17314.

(7) Cabello-Garcia, J.; Bae, W.; Stan V, G.-B.; Ouldridge, T. E. Handhold-Mediated Strand Displacement: A Nucleic Acid Based Mechanism for Generating Far-from-Equilibrium Assemblies through Templated Reactions. *ACS Nano* **2021**, *15*, 3272–3283.

(8) Juritz, J.; Poulton, J. M.; Ouldridge, T. E. Minimal mechanism for cyclic templating of length-controlled copolymers under isothermal conditions. *J. Chem. Phys.* **2022**, *156*, 074103.
